# Allelic variation for broad‐spectrum resistance and susceptibility to bacterial pathogens identified in a rice MAGIC population

**DOI:** 10.1111/pbi.12895

**Published:** 2018-03-08

**Authors:** Ana M. Bossa‐Castro, Cheick Tekete, Chitra Raghavan, Emily E. Delorean, Alexis Dereeper, Karim Dagno, Ousmane Koita, Gloria Mosquera, Hei Leung, Valérie Verdier, Jan E. Leach

**Affiliations:** ^1^ Department of Bioagricultural Sciences and Pest Management Colorado State University Fort Collins CO USA; ^2^ IRD Cirad IPME Univ Montpellier Montpellier France; ^3^ Faculté des Sciences et Techniques LBMA Université des Sciences Techniques et Technologiques Bamako Mali; ^4^ Division of Plant Breeding, Genetics and Biotechnology International Rice Research Institute Manila Philippines; ^5^ Plant Protection Institute of Rural Economy Sotuba Mali; ^6^ Centro Internacional de Agricultura Tropical Cali Colombia; ^7^ Present address: Horticulture and Forestry Sciences Queensland Department of Agriculture and Fisheries Cairns QLD Australia; ^8^ Present address: Department of Plant Pathology Kansas State University Manhattan KS USA

**Keywords:** broad‐spectrum resistance, multiparent advanced generation intercross, *Xanthomonas oryzae*, Africa, bacterial leaf streak, bacterial blight

## Abstract

Quantitative trait loci (QTL) that confer broad‐spectrum resistance (BSR), or resistance that is effective against multiple and diverse plant pathogens, have been elusive targets of crop breeding programmes. Multiparent advanced generation intercross (MAGIC) populations, with their diverse genetic composition and high levels of recombination, are potential resources for the identification of QTL for BSR. In this study, a rice MAGIC population was used to map QTL conferring BSR to two major rice diseases, bacterial leaf streak (BLS) and bacterial blight (BB), caused by *Xanthomonas oryzae* pathovars (pv.) *oryzicola* (*Xoc*) and *oryzae* (*Xoo*), respectively. Controlling these diseases is particularly important in sub‐Saharan Africa, where no sources of BSR are currently available in deployed varieties. The MAGIC founders and lines were genotyped by sequencing and phenotyped in the greenhouse and field by inoculation with multiple strains of *Xoc* and *Xoo*. A combination of genomewide association studies (GWAS) and interval mapping analyses revealed 11 BSR QTL, effective against both diseases, and three pathovar‐specific QTL. The most promising BSR QTL (qXO‐2‐1, qXO‐4‐1 and qXO‐11‐2) conferred resistance to more than nine *Xoc* and *Xoo* strains. GWAS detected 369 significant SNP markers with distinguishable phenotypic effects, allowing the identification of alleles conferring disease resistance and susceptibility. The BSR and susceptibility QTL will improve our understanding of the mechanisms of both resistance and susceptibility in the long term and will be immediately useful resources for rice breeding programmes.

## Introduction

Disease resistance in rice (*Oryza sativa* L.) is classified into two main categories, qualitative and quantitative. Qualitative resistance is controlled by a single resistance (*R*) gene and is usually effective against only certain strains of a pathogen species. As it imposes a greater selection pressure on pathogen evolution, this type of resistance can be more readily overcome. Conversely, quantitative resistance is conferred by quantitative trait loci (QTL), encompassing multiple genes, and is frequently associated with partial but durable resistance to an entire pathogen species or even different pathogen genera (Boyd *et al*., 2013; Wisser *et al*., 2005). Quantitative resistance is frequently referred to as broad‐spectrum resistance (BSR), because of the breadth of pathogen groups it affects (Wisser *et al*., 2005).

The deployment of disease resistance QTL in rice has been limited due to its complex genetic control and the lack of knowledge on the function of genes underlying the QTL (Ramalingam *et al*., 2003). Recently, novel resources for association and interval mapping studies in rice, the multiparent advanced generation intercross (MAGIC) populations, were developed and demonstrated to expedite identification of QTL (Bandillo *et al*., 2013; Cavanagh *et al*., 2008; Raghavan *et al*., 2017). Each rice MAGIC population was generated from multiple founder lines, selected for their different agronomic traits and genetic background. The founders were crossed in a design that ensured an equal representation of each founder in the population. Benefits of MAGIC populations to QTL mapping are increased recombination, transgressive segregation events revealing novel phenotypes and capacity for fine mapping, which expedite the discovery of new resistance sources against pathogens (Bandillo *et al*., 2013; Raghavan *et al*., 2017).

Bacterial leaf streak (BLS) and bacterial blight (BB) are devastating diseases of rice that cause considerable losses and yield reductions, especially in Asia and Africa (Cernadas *et al*., 2014; Mew *et al*., 1992). In Africa, the expansion and intensification of crop cultivation, coupled with the absence of appropriate phytosanitary controls, have contributed to the increased incidence of BLS and BB and the emergence of more virulent pathogen strains (Verdier *et al*., 2012; Wonni *et al*., 2014). BB has been an important threat in Africa since 1980, causing up to 50% yield loss, while BLS is a more recently observed disease, causing up to 20% yield loss (Kang *et al*., 2008; Reddy *et al*., 1979; Wonni *et al*., 2015). In Asia, the use of resistant varieties is considered the most effective and sustainable way to control these diseases (Tang *et al*., 2000; Verdier *et al*., 2012), but sources of resistance for BLS and BB in Africa are lacking (Wonni *et al*., 2015). BLS and BB are caused by two closely related pathogens, *Xanthomonas oryzae* pv. *oryzicola* (*Xoc*) and *Xanthomonas oryzae* pv. *oryzae* (*Xoo*), respectively. *Xoc* is a nonvascular pathogen that enters the plant through stomata or wounds and colonizes intercellular spaces of mesophyll parenchyma (Ou, 1985), while *Xoo* is a vascular pathogen that enters through hydathodes or wounds, causing a systemic infection (Tabei, 1977). Although African *Xoc* and *Xoo* strains elicit similar symptoms to their Asian counterparts, they display several genetic differences and are grouped into distinct phylogenetic clades (Gonzalez *et al*., 2007; Wonni *et al*., 2014).

Over 40 *R* genes against Asian *Xoo* strains have been mapped (Hutin *et al*., 2015; Kim *et al*., 2015); however, most of these *R* genes are not effective in controlling the distinct strains of *Xoc* and *Xoo* present in sub‐Saharan Africa (Gonzalez *et al*., 2007). Djedatin *et al*. recently mapped disease resistance QTL effective against African *Xoo* strains, located on rice chromosomes 1, 7, 9, 10 and 11 (Djedatin *et al*., 2016). Interestingly, the QTL on chromosome 11 confers resistance to both Asian and African *Xoo*. QTL for Asian *Xoc* have also been identified (Tang *et al*., 2000; Xie *et al*., 2014), but no QTL to African *Xoc* have yet been detected. Despite numerous efforts, only two resistance loci to *Xoc* are currently known in rice. These include the recessive resistance gene *bls1* effective against Asian *Xoc* (He *et al*., 2012) and the locus *Xo1* effective against multiple African *Xoc* and *Xoo* strains (Triplett *et al*., 2016). *Xo1* colocalizes with the *R* gene *Xa1*, which confers resistance to Asian *Xoo*, and work is in progress to determine whether they correspond to the same gene (Triplett *et al*., 2016). Additionally, *Rxo1*, a gene cloned from maize, confers nonhost resistance in rice plants against *Xoc* (Zhao *et al*., 2005). Previous studies argued that resistance to BLS is mostly quantitative (Tang *et al*., 2000), possibly explaining why few single *R* loci have been detected so far (Makino *et al*., 2006). As related pathogens, *Xoc* and *Xoo* use transcription activator‐like (TAL) effectors as their primary virulence factors to manipulate rice gene expression and invade the host (Bogdanove and Voytas, 2011). Rice genes that promote infection, such as some genes activated by TAL effectors, are considered susceptibility (*S*) genes (Boch *et al*., 2014). A disease resistance‐associated region can have either the alleles that contribute to a resistant phenotype or the susceptible alleles that lead to increased disease. Because *S* gene activation is essential for *X. oryzae* virulence, the mutation of an *S* gene can decrease the ability to invade the host. The resulting resistance is therefore achieved by the loss of susceptibility (Boch *et al*., 2014).

For this study, we selected a MAGIC population developed from eight indica founders (Table S1). Indica is one of the eco‐geographical subspecies of rice grown widely cultivated in the tropics and the subtropics, including African countries (Garris *et al*., 2005). Our goal was to use the indica MAGIC population to identify loci (QTL/genes) associated with resistance and susceptibility to BLS and BB of rice and, specifically, to identify loci that confer BSR to both diseases. Our primary focus was on strains of *Xoc* and *Xoo* from sub‐Saharan Africa, due to the lack of effective BLS or BB resistance in currently cultivated germplasm in this region (Verdier *et al*., 2012). MAGIC indica founders had not been previously screened for resistance to African *Xoc* and *Xoo* strains. In general, BSR effective against multiple bacterial pathogens is a powerful resource for enhancement of local elite varieties or advanced lines from regional breeding programmes. Importantly, because highly useful new prebreeding materials can be extracted directly from MAGIC populations, this approach can significantly expedite the deployment of durable *X. oryzae*‐resistant varieties.

## Results

### MAGIC indica population reveals sources of resistance to *Xoc* and *Xoo* strains

Two subsets of the MAGIC population were chosen at early (fourth, called S4) and advanced (sixth‐eighth, called S8) selfed generations, consisting of 200 and 340 advanced intercross lines (AILs), respectively. Both subsets were used to map QTL/genes associated with resistance and susceptibility, and to evaluate the suitability of MAGIC populations for the discovery of disease resistance‐associated regions. In addition, the resolution of QTL detection was compared for each generation (early and advanced).

The MAGIC S4 subset was screened with two *Xoc* and two *Xoo* African strains (Table S2). The responses to *Xoc* BAI5 and MAI3, and *Xoo* BAI3 and MAI1, measured as lesion length (LL), exhibited a large range of phenotypic variation when compared to the phenotypes of the founders (Figure S1). The phenotypic variation was expected, due to the high degree of recombination in this population. A high number of resistant AILs (0 < LL ≤ 0.1 cm for *Xoc* and 0 < LL < 5 cm for *Xoo*) were observed for strains *Xoc* BA5 and MAI3, and *Xoo* MAI1, whereas *Xoo* BAI3 showed a more normal distribution of phenotypes. Overall, these results demonstrated potential sources of resistance for BLS and BB in the MAGIC indica population.

**Figure 1:**
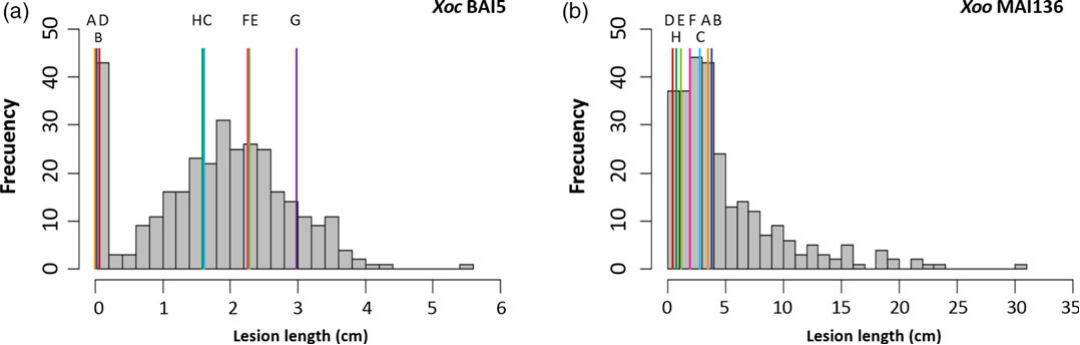
Distribution of lesion length (cm) of MAGIC indica founders and S8 subset. (a) Screening of 323 AILs in the greenhouse with *Xoc* BAI5. (b) Screening of 276 AILs in the field with *Xoo* MAI136. Histograms represent mean values of MAGIC lines. Mean lesion lengths of founders are indicated with vertical lines (A: IR4630‐22‐2‐5‐1‐3; B: Fedearroz 50; C: IR77298‐14‐1‐2‐10; D: Shan‐Huang Zhan‐2; E: PSBRc82; F: Sambha Mahsuri + Sub1; G: PSBRc158; H: IR45427‐2B‐2‐2B‐1‐1). For *Xoo* MAI136, phenotypic value of founder PSBRc158 is missing due to lack of germination.

The MAGIC S8 subset was screened using a larger set of strains with diverse genetic backgrounds and isolated from different geographical regions. A total of 20 *X. oryzae* strains, nine *Xoc* and 11 *Xoo*, were used for screening the S8 AILs and founders in the greenhouse and under field conditions in Mali (Figures 1, S2 and S3; Table S2). Two common strains were used to phenotype the S4 and the S8 (*Xoc* BA5 and *Xoo* BAI3). Phenotypic responses to most strains in both the greenhouse and the field displayed a right‐skewed distribution, while some showed an approximately normal distribution (Figures 1, S2 and S3). As observed in the S4 screenings, founders responded differently to each strain, indicating different resistance sources. For strains *Xoc* BLS256 and *Xoo* MAI133, transgressive segregation for resistance was observed in the S8, showing AILs with more resistant phenotypes than any of the founders. Interestingly, for all the strains tested, transgressive segregation for susceptibility was observed in the MAGIC AILs (Figures 1, S2 and S3), thus positioning the indica MAGIC as a valuable population for studying susceptibility‐associated loci.

### Multiple disease resistance QTL identified in the MAGIC indica population

Founders and the S4 subset were genotyped using the genotyping‐by‐sequencing (GBS) method (Bandillo *et al*., 2013; Elshire *et al*., 2011), as were 1,316 MAGIC AILs of the S6:S8 generation (Raghavan *et al*., 2017). For this study, monomorphic SNP markers were excluded, and therefore, the final data sets contained SNPs for which at least one of the parents was polymorphic. Genomewide association studies (GWAS) and interval mapping (IM) adapted to multiparent populations were conducted to detect genomewide associations and QTL, using the SNP marker data sets for each bacterial strain and MAGIC population subset (Table S3).

**Figure 2:**
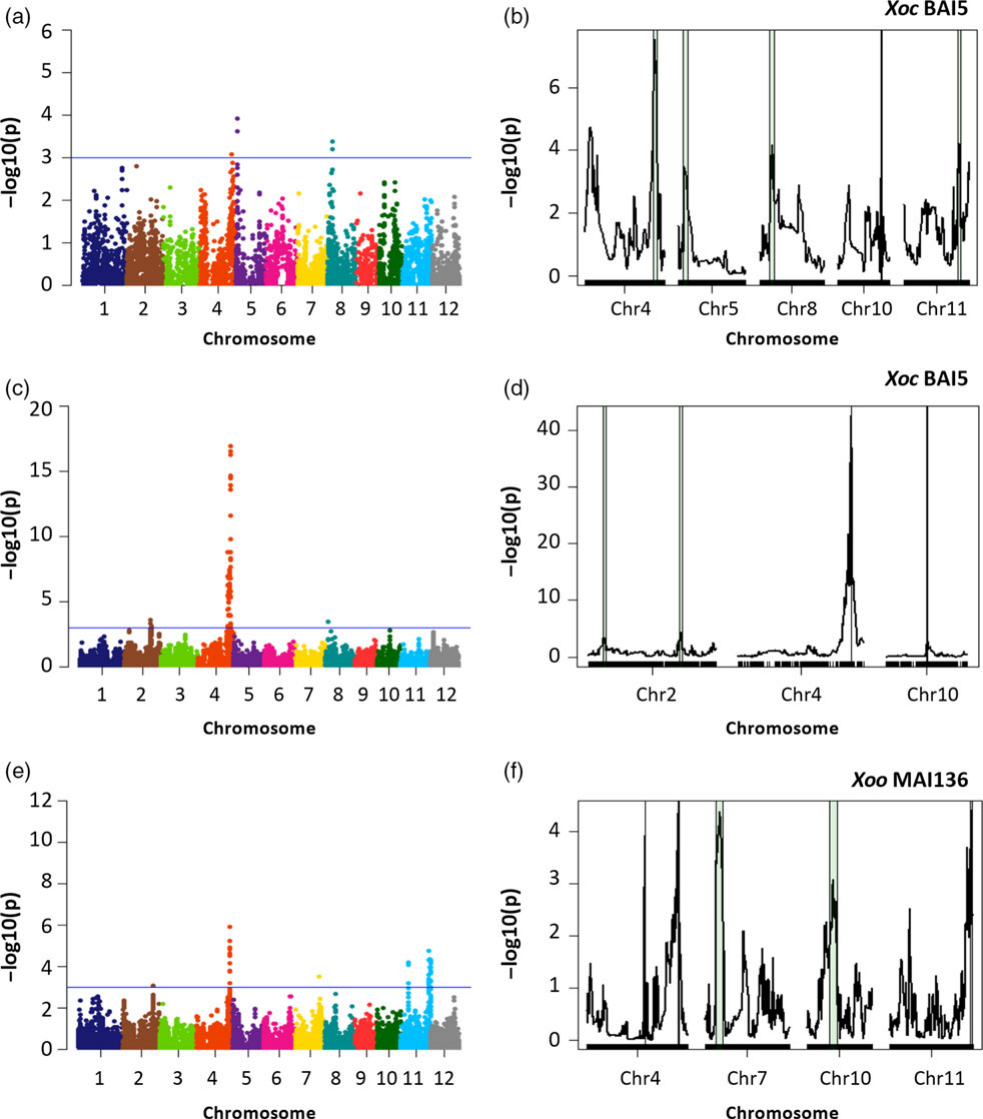
Quantitative trait loci (QTL) detection for resistance to *Xoc* BAI5 and *Xoo* MAI136 in MAGIC indica S4 and S8 subsets. (a,b) *Xoc* BAI5 (markers = 7258), S4 subset. (c,d) *Xoc* BAI5 (markers = 14 475), S8 subset. (e,f) *Xoo* MAI36 (markers  = 14 475), S8 subset. (a,c,e) Manhattan plots show the negative logarithm of the *P*‐values for the mixed linear model, by chromosome. Solid blue line indicates significance threshold (*P*‐value < 0.001). (b,d,f) Simple interval mapping showing chromosomes with significant QTL (*P*‐value < 0.001). Green regions indicate 1‐LOD support intervals.

For GWAS, the founders were excluded, as this methodology considers individuals to be unrelated. The MAGIC indica population has a negligible population structure (Bandillo *et al*., 2013; Mackay and Powell, 2007), allowing these studies. A kinship matrix was generated for each subset‐bacterial strain data set, and then, analyses were performed using the mixed linear model (MLM) for S4 and S8 subsets. Regions associated with resistance to *Xoc* or *Xoo* were detected for all bacterial strains screened in the S4, with 42 significant SNPs (*P*‐value < 0.001), on chromosomes 4, 5, 8, 9 and 11 (Figures 2a, and S4a,c,e; Table S4). GWAS on the S8 yielded 369 SNP significant SNPs (*P*‐value < 0.001) and identified additional regions for resistance to *Xoc* or *Xoo* in all 12 chromosomes, with the most significant associations found on chromosomes 2, 4, 5, 7, 10 and 11 (Figures 2c,e, S5 and S6; Table S5). The GWAS approach detected two loci on chromosome 11, at 6–8 Mpb and 26–29 Mbp, for resistance to four *Xoo* strains (Figures 2e and S6a,e,k). The majority of the 369 significant SNPs were located in or near gene promoters (40), coding sequences (185), introns (43) and untranslated (28) regions (Table S5). The significance of the associations was notably higher in the S8 compared to the S4 for the same *X. oryzae* strain for three main reasons. The S8 is an advanced generation where alleles are fixed, as opposed to the S4 where alleles are still segregating, the number of the SNP markers used in the S8 was almost double that of the S4, and because a larger number of MAGIC AILs were screened in the S8 (Figures 2a,c, S4c and S6a).

For IM, genotypic and pedigree information from the AILs was used to generate a linkage map for each subset. QTL were determined using simple interval mapping, computing the founder effects between each pair of markers. A Wald test for the significance of all founder effects at each putative QTL position was performed. QTL were called when *P*‐value < 0.001, and supporting intervals were calculated as the 95% confidence interval (1‐LOD). A total of 19 disease resistance QTL were detected in the S4, located in almost all chromosomes, except 6 and 12 (Figures 2b, and S4b,d,f; Table S6). As expected, the two mapping approaches identified identical genetic regions; that is, IM supporting intervals contained markers significantly associated with GWAS in the S4 (Table S4). Overlapping genetic regions associated with disease resistance for both *X. oryzae* pathovars were mapped on chromosomes 4 for *Xoc* BAI5, MAI3 and *Xoo* BAI3, and on 11 for *Xoc* MAI3, and *Xoo* BAI3, at 30–32 Mbp and 27–29 Mbp, respectively (Table S6). The region on chromosome 4 colocalizes with a recently detected locus *Xo1* that is effective against multiple *Xoc* and *Xoo* strains (Triplett *et al*., 2016), and the region on chromosome 11 was reported to contain resistance for *Xoo* but not for *Xoc* (Djedatin *et al*., 2016).

Interval mapping analyses on the S8, performed independently for each strain and using 14 475 SNP markers, detected significant associations in all chromosomes except chromosome 3 (Figures 2b,d,f, S5 and S6; Table S7) and revealed novel genetic regions associated with resistance to BLS and BB. More associated regions were mapped using IM than GWAS for each strain, with up to three QTL within a single chromosome (Table S7). As observed in the S4, some identified loci were corroborated by the two approaches. A total of 37 strain‐specific QTL were detected in the S8, with an average of five QTL per bacterial strain.

Overall, we identified multiple QTL associated with resistance to BLS and BB in both S4 and S8 subsets of the indica MAGIC population, many of which were confirmed by both GWAS and IM. While the earlier generation (S4) was sufficient to map regions associated with disease resistance, the S8 results yielded SNPs associated at lower *P*‐values by GWAS and provided more precision for determining the location of the QTL by IM. In fact, the size of the supporting intervals detected in the S4 subset was notably reduced in the S8, for QTL on chromosomes 4, 5, 10 and 11 (Table S8).

### MAGIC indica population uncovers multiple BSR QTL and allelic contributions to resistance and susceptibility

**Figure 3:**
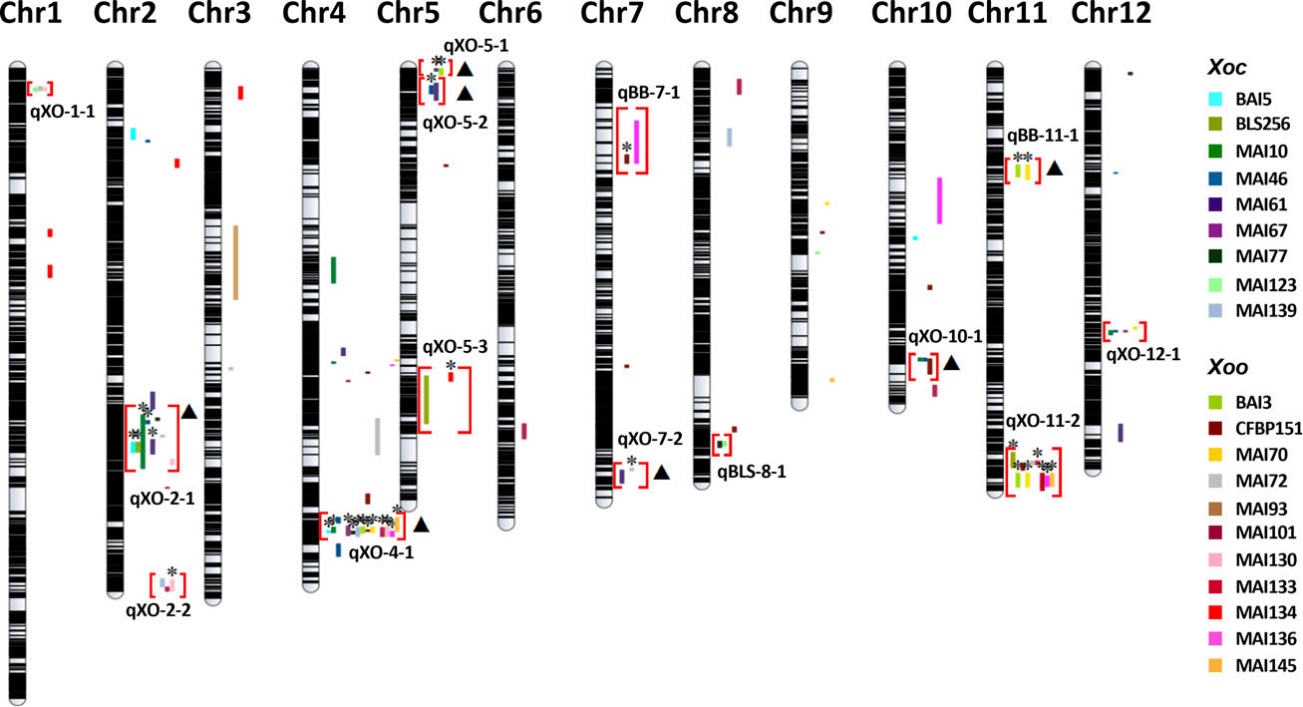
Integrative map showing resistance QTL to African and Asian *Xoc* and *Xoo* found in this study. Horizontal black lines represent the physical map using 14 475 SNP markers. Solid coloured lines next to chromosomes indicate supporting interval sizes for each strain‐specific QTL detected (Table S7). *Xoc* and *Xoo* strains are differentiated by colours, as listed on the right. Red brackets highlight the 14 QTL that confer resistance to multiple *X. oryzae* strains. QTL names indicate whether it confers BSR, named qXO, or pathovar‐specific resistance, named qBLS and qBB, for *Xoc* and *Xoo*, respectively. Stars (*) above supporting intervals indicate QTL corroboration by GWAS; triangles (▲) indicate GWAS corroboration of the QTL by strains other than those in the IM.

Integrating the results of GWAS and IM from the 20 *X. oryzae* strains in the S8, we identified 14 regions associated with resistance to multiple bacterial strains (Figure 3). Of these loci, 11 QTL, named qXO, are effective against both *Xoc* and *Xoo*, and three QTL, named qBLS and qBB, are specific to one pathovar. To define the limits of the pathovar‐specific and BSR QTL, we selected the widest overlapping genetic region from IM analyses as the common QTL (Table 1 and Table S9). Most QTL were identified by both mapping approaches with the same pathogen strains, and some of these same QTL were detected by additional strains in GWAS. Four QTL were detected only by IM. Of the 369 significant SNPs yielded by GWAS across all strains, 103 SNPs were effective to multiple strains and located on QTL qXO‐2‐1, qXO‐4‐1, qXO‐5‐1, qBB‐11‐1 and qXO‐11‐2. Overall, we detected 51 QTL in the S8 (11 BSR QTL, three pathovar‐specific and 37 strain‐specific). From the 19 QTL detected in the S4, five overlapped with the BSR QTL qXO‐2‐2, qXO‐4‐1, qXO‐5‐2, qXO‐10‐1 and qXO‐11‐2 identified in the S8 (Table S6). The locations of QTL common to the S4 and S8 as well as the QTL sizes were refined in the S8, largely due to an increased number of SNPs, a larger sample size and the fact that the S8 is a more advanced generation where alleles are fixed (Table S8).

**Table 1:** Quantitative trait loci (QTL) effective to multiple *X. oryzae* strains identified in this study

QTL	Chr	QTL position			Pathovar	No. of strains	Known resistance loci to	
		(cM)	Left Mrk	Right Mrk			African *X. oryzae*	Asian *X. oryzae*
qXO‐1‐1	1	5.2–5.9	S1_1335951	S1_1500023	both	3		
qXO‐2‐1	2	96.3–107.8	S2_24122049	S2_26993900	both	12		Qbr2a
qXO‐2‐2	2	141.1–142.9	S2_35289602	S2_35781025	both	3		AQBT001, qBbr2b, *Xa24(t)*
qXO‐4‐1	4	125.8–127.7	S4_31553264	S4_32064419	both	16	*Xo1*	AQBT008, *Xa1, Xa2, Xa31(t), Xa38*
qXO‐5‐1	5	0.1–1.3	S5_69530	S5_353165	both	3		qBLSr5a, qBbr5*, xa5*
qXO‐5‐2	5	5.9–8.0	S5_1494420	S5_2046183	both	4		
qXO‐5‐3	5	84.9–90.7	S5_21255253	S5_22750867	both	2		qBB‐5‐2, qBB5, qBB‐5‐2, AQW004, qBBR5
qBB‐7‐1	7	20.1–23.6	S7_5097414	S7_5993972	*Xoo*	2	qABB‐7	*xa8*
qXO‐7‐2	7	109.9–112.3	S7_27578266	S7_28179129	both	3		
qBLS‐8‐1	8	102.6–102.7	S8_25638183	S8_25729831	*Xoc*	2		
qXO‐10‐1	10	79.6–80.8	S10_19975243	S10_20983368	both	5	qABB‐10	
qBB‐11‐1	11	28.0–28.9	S11_7012013	S11_7244498	*Xoo*	5		
qXO‐11‐2	11	107.5–114.6	S11_26879946	S11_28697227	both	9	qABB‐11	QBbr11, AQBT023, *Xa3/Xa26, Xa4, Xa22, Xa32(t), Xa35(t), Xa36(t), Xa40*
qXO‐12‐1	12	70.7–71.8	S12_17786177	S12_18084420	both	4		AQBT029

Individual IM results (Table S7) were combined using the widest supporting interval shared by the different *X. oryzae* strains in overlapping genetic regions. Detailed information is shown in Table S9. Number of strains combines the total number of strains associated with each QTL by GWAS and IM analyses. Known resistance loci to African or Asian *X. oryzae* refer to previously identified QTL and single resistance genes (denoted by the prefix *Xa* or *Xo*).

**Figure 4:**
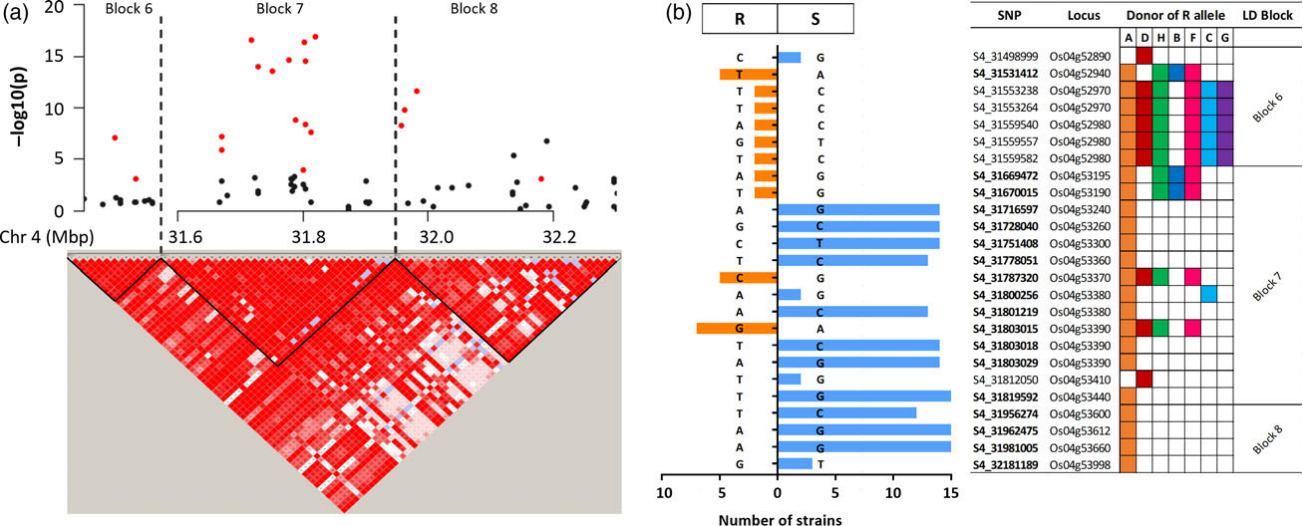
Haplotype block analyses and SNP effects in a hot spot region for *X. oryzae* resistance within qXO‐4‐1. (a) Local Manhattan plot of *Xoc* BAI5 (top) and linkage disequilibrium (LD) with haplotype block analysis (bottom) of the 31.49–32.29 Mbp region on chromosome 4. Red filled circles indicate SNPs that are significant to multiple *X. oryzae* strains. LD heatmap shows the standard Haploview colour scheme to display LD with bright red for strong LD (LOD = 2 D’ = 1), pink (LOD = 2 D’ < 1) and blue (LOD < 2 D’ = 1) for intermediate LD, and white for no LD (LOD < 2, D’ < 1). (b) Summary of SNP effects to *X. oryzae* strains within haplotype blocks 6, 7 and 8 on chromosome 4. Size of the bars on the left denotes the number of strains for which a SNP was significant (*P*‐value < 0.001); colour and direction of the bars indicate the estimated effect, either negative (left, orange) or positive (right, blue). A negative effect is associated with a more resistant phenotype (R) and a positive effect with a more susceptible phenotype (S). For each SNP, the correspondent allele for the estimated effect is shown. SNPs in bold denote the SNP was significant in both pathovars (*Xoc* and *Xoo*), and otherwise, the SNP was significant only for *Xoc* strains. The locus ID for each SNP (prefix ‘LOC_’ is omitted) was predicted from the MSU7 rice reference annotation. Founders are indicated with letters (A: IR4630‐22‐2‐5‐1‐3; B: Fedearroz 50; C: IR77298‐14‐1‐2‐10; D: Shan‐Huang Zhan‐2; F: Sambha Mahsuri + Sub1; G: PSBRc158; H: IR45427‐2B‐2‐2B‐1‐1). A coloured box on the donor column indicates that the founder carries the R allele.

One advantage of performing both GWAS and IM, beyond confirmation of the identified QTL, is that clusters of significant SNPs within the QTL can be identified to fine map the causal genes. The most promising QTL in our study were qXO‐2‐1, qXO‐4‐1 and qXO‐11‐2, as they confer resistance to 12, 16 and nine *X. oryzae* strains, respectively. These QTL explained 6.3%–13.2%, 7.0%–39.5% and 6.9%–12.3% of the phenotypic variance to *X. oryzae* strains, respectively. Furthermore, for these three QTL, several SNPs were significantly associated with multiple strains (Table S5). We selected the overlapping genetic region for each QTL where common SNPs were detected by different strains, 24.5–27.2 Mbp, 29.5–32.6 Mbp and 25.1–28.9 Mbp in chromosomes 2, 4 and 11, respectively. Linkage disequilibrium (LD) analyses performed within these regions identified haplotype blocks (Figures S7, S8 and S9). More than five SNPs significantly associated with resistance to multiple *X. oryzae* strains were clustered in a few haplotype blocks in each chromosome (Figures S7, S8 and S9; Tables S10, S11 and S12). These results provide useful information to narrow the genetic region associated with resistance for each one of these QTL. For instance, on chromosome 4, 26 of the 39 common significant SNPs were located in haplotype blocks 6, 7 and 8. SNPs on blocks 7 and 8 were associated with resistance to more than 15 strains (Figures 4a and S8; Table S11), and the putative donor of the resistant alleles in this region was the founder IR4630‐22‐2‐5‐1‐3 (Table S11).

Integration of GWAS and IM in multiparent populations can help in assessing the phenotypic effects of single allelic variants at each SNPs. All the SNP in the S4 and S8 subset are dimorphic; that is, there are only two possible alleles for each marker. GWAS estimate the effect of each allele at a SNP; in this case, a negative effect indicates the ‘effect allele’ is associated with shorter lesion lengths (thus associated with a more resistant phenotype) and a positive effect indicates the opposite. For the majority of the identified QTL, the effect (causal) alleles were predicted to have negative effects (Table S5). Interestingly, in qXO‐2‐1 and qXO‐4‐1, several alleles had positive effects, indicating their association with an increase in susceptibility. In qXO‐4‐1, 11 SNPs located in blocks 7 and 8 are associated with increased susceptibility to 12–15 *X. oryzae* strains (Figure 4b).

In summary, multiple BSR sources to *Xoc* and *Xoo* of particular importance to rice producing areas of sub‐Saharan Africa were identified. Moreover, our integration of GWAS and IM results enabled refinement of the mapped QTL. By detecting phenotypic effects of causal alleles, we have identified resources that will facilitate a better understanding of how the affected genes contribute to resistance or susceptibility.

## Discussion

### MAGIC populations are powerful resources for discovery of novel, broad‐spectrum disease resistance

In this study, we used an indica MAGIC population to identify QTL exhibiting broad‐spectrum resistance to two bacterial diseases of rice, BLS and BB. Our results are particularly relevant for sub‐Saharan Africa, given the increasing incidence of these diseases and the lack of resistance in currently used rice germplasm. In total, we identified 14 disease resistance QTL effective against multiple *X. oryzae* strains; of these, 11 are BSR QTL (Figure 3 and Table 1). In addition, we identified 37 strain‐specific QTL (Table S7). Of the total 51 QTL, 49 were confirmed under field screening conditions, supporting their value in the development of improved varieties.

Three types of novel resistance QTL for BLS and BB disease resistance were identified in this study, including 25 strain‐specific QTL (Table S7), two pathovar‐specific QTL (qBLS‐8‐1 for BLS and qBB‐11‐1 for BB) and three BSR QTL (qXO‐1‐1, qXO‐5‐2, XO‐7‐2). Although some of the identified BSR QTL colocalize with formerly described loci or QTL for Asian *X. oryzae* strains (e.g. qXO‐2‐1, qXO‐2‐2, qXO‐5‐1, qXO‐5‐3 and qXO‐12‐1), these regions were not shown previously to confer resistance to African *X. oryzae* strains. We used a larger and more diverse set of strains, including different *Xoo* races, and demonstrated a broader effectiveness for the previously identified loci qXO‐4‐1, qBB‐7‐1, qXO‐10‐1 and qXO‐11‐2 (Djedatin *et al*., 2016; Triplett *et al*., 2016). Two QTL, qXO‐4‐1 and qXO‐11‐2, coincide with clusters of *R* genes previously identified for Asian *Xoo* resistance on chromosome 4 (*Xa1*,* Xa2*,* Xa12*,* Xa14*,* Xa31* and *Xa38*) and chromosome 11 (*Xa3*/*26*,* Xa4*,* Xa10*,* Xa21*,* Xa22* and *Xa23*) (Kim *et al*., 2015). *Xa4* and *xa5* are present in some of the MAGIC founders (Table S1). However, these *R* genes are only reported to confer resistance to *Xoo,* and thus, they do not confer BSR. The BSR QTL qXO‐4‐1 and qXO‐11‐2, together with qXO‐2‐1, confer resistance to a large number of *X. oryzae* strains and are excellent targets for further validation and use in crop improvement programs.

MAGIC populations include multiple founders, which endows greater genetic diversity, and their construction involves several crossing events, which increases shuffling of the progeny's genomes. For these reasons, a single MAGIC population can be used to study multiple traits and, as we observed in this study, can reveal more QTL than a single biparental population (Bohra, 2013). Multiparent populations also allow the possibility of using smaller sample sizes and earlier generations to identify traits of agricultural interest. We conducted QTL mapping in early and advanced generations, the S4 and S6:S8, respectively, and found that the former is suitable for coarse mapping while the latter allows for fine mapping (Cavanagh *et al*., 2008), with reduced intervals, and confirmation of the associations found in the early generation (Table S8). Moreover, by combining GWAS and IM approaches, we identified alleles associated with resistance and susceptibility, which will help assess the mechanisms of resistance in these QTL.

### Understanding the resistance and susceptibility mechanisms to *X. oryzae* in rice

Despite advances in understanding how Asian *X. oryzae* elicit resistance or susceptibility in the host, the knowledge of resistance and susceptibility mechanisms to African *X. oryzae* is limited (Djedatin *et al*., 2016; Triplett *et al*., 2016). Both *Xoc* and *Xoo* contain and use TAL effectors for virulence, but their TAL repertoires are distinct (Cernadas *et al*., 2014). Moreover, rice gene expression patterns during infection with *Xoc* and *Xoo* are very different (Cernadas *et al*., 2014), suggesting that, in addition to differences in their tissue specificity, these pathogens differ in their virulence mechanisms. Rice *S* genes targeted by *Xoo* TAL effectors are different than those targeted by *Xoc* (Cai *et al*., 2017; Verdier *et al*., 2012). For instance, several *Xoo* TAL effectors target host sucrose transporter genes (Chen *et al*., 2010), while some *Xoc* TAL effectors target sulphur transporter genes (Cernadas *et al*., 2014).

Mutation of target *S* genes results in one type of host resistance, that is the loss of susceptibility (Boch *et al*., 2014). These mutations can occur in promoters, blocking the ability of the TAL to bind and activate, or in coding regions, changing how effectors interact with the susceptibility target (Huang *et al*., 2016). In our study, we found variants in promoters (40/369) and in coding regions (185/369) of potential *S* genes (Table S5). In the near‐term, these allelic variants can inform marker‐assisted selection strategies for germplasm improvement. These specific alleles pinpoint gene candidates for functional analysis, the next step to revealing how the allelic variants contribute to resistance and susceptibility.

Remaining questions are (i) whether the same or different genes within each QTL are involved in the defence response to each of the *X. oryzae* pathovars, (ii) whether there are QTL interactions that favour the defence response and (iii) how the QTL are transcriptionally modulated.

Few resistance sources effective against diverse pathogens (broad‐spectrum resistance, BSR) are available for crop improvement programs. BSR providing protection to two important rice diseases, bacterial blight and bacterial leaf streak, is particularly needed for African rice producing areas, where the resistance to both diseases is lacking. In this study, we identified 11 BSR QTL to BLS and BB diseases using a rice MAGIC indica population. The QTL were confirmed in greenhouse and field studies. Our work shows that the implementation of multiparent populations for the study of disease resistance, in combination with genomewide association and interval mapping analyses, can facilitate the discovery of BSR QTL. Moreover, the integration of these approaches is used to refine QTL and to improve the understanding of their contributions to resistance or susceptibility. Because the MAGIC founders are elite varieties, the disease resistance QTL identified here can be rapidly incorporated into breeding programmes to achieve more durable resistance to BLS and BB.

## Experimental procedures

### Plant materials

The crossing strategy and development of the MAGIC indica mapping population used in this study were previously described (Bandillo *et al*., 2013). Briefly, the eight founders were intermated for a total of 28 biparental crosses. To derive 4‐way crosses, the 28 F1s were then intercrossed, but only 70 crosses (of the possible 210 crosses) were performed. The 8‐way crosses were derived from intercrossing the 70 4‐way crosses, but only 35 (of the possible 105) 8‐way crosses were performed. From each of the 35 8‐way crosses, approximately 60 seeds were advanced by selfing, achieving a population size of approximately 2100 advanced intercross lines (AILs). At the fourth selfed generation, a subset of 200 AILs (S4 subset) was randomly selected for screening (Bandillo *et al*., 2013). At the sixth–eighth selfed generation (S6:S8), 340 AILs were selected (approximately 10 random lines from 33 8‐way crosses) for screening (called the S8 subset).

### Genotyping and filtering of S4 and S8 subsets

Founders and the S4 subset were genotyped using a 96‐plex *Ape*KI genotyping by sequencing (GBS) protocol (Bandillo *et al*., 2013; Elshire *et al*., 2011). The Nipponbare reference genome was used for SNP calling by the TASSEL GBS analysis pipeline v. 3.0.147 (Glaubitz *et al*., 2014). The same procedure was repeated for the eight founders and 1316 MAGIC AILs of the S6:S8 generation (Raghavan *et al*., 2017); SNP calling was carried out using TASSEL GBS analysis pipeline v. 3.0.169 (Glaubitz *et al*., 2014).

Initial data sets of 634 103 and 396 361 SNP markers were obtained for the S4 and S8 subsets, respectively. Markers with missing calls, heterozygous and monomorphic markers in the founders were removed from both data sets. Missing calls were not imputed for any analysis. For the S4 subset, SNP markers for each AILs‐bacterial strain data set were filtered so that 80% of the AILs had a call and a minimum frequency of 0.05 for the minor allele, using TASSEL v 4.3.4 (Bradbury *et al*., 2007). Resulting SNP markers for each strain are listed in Table S3. For the S8 subset, SNP markers were filtered so that 80% of the AILs had a call and a minimum frequency of 0.05 for the minor allele, resulting in a set of 14 475 SNP markers, using TASSEL v 5.0.2 (Bradbury *et al*., 2007). The same SNP data sets for each bacterial strain in S4 and S8 subsets were used for both GWAS and IM (Table S3).

### Greenhouse and field conditions

Plants screened in growth chamber and greenhouse were grown individually in pots containing 1 : 1 : 0.25 mixture of Pro‐mix potting mix, peat moss and sand. Fertilizer (Peters Excel 15‐5‐15 Cal‐Mag (Scotts) 300 mg/L) was applied twice per week, beginning at 2 weeks after germination.

The growth chamber conditions throughout the experiment were 16‐h light/8‐h dark at 28 °C day/24 °C night with an average relative humidity of 85% (used only for S4 screening of *Xoc* strains BAI5 and MAI3). The greenhouse conditions throughout the experiment were 16‐h light/8‐h dark at 30 °C day/25 °C night, with an average relative humidity of 75%.

The screening of MAGIC AILs in field conditions was conducted at the agronomic research station IER Sotuba (Bamako, Mali) from June to September 2015. Seeds were sown in pots filled with local compost. About 5 g of DAP (18‐46‐0) was added to each pot on the 15th, 25th and 35th days after sowing. The average temperature was 28 °C with a minimum of 23 °C and maximum of 33 °C. The total precipitation during this period was 680 mm with an average relative humidity of 74% (61% minimum to 88% maximum).

### Inoculations and phenotyping

Cultures of *Xoc* and *Xoo* were incubated for 24 h on peptone‐sucrose agar (PSA) medium (Karganilla *et al*., 1973) at 28 °C. For inoculum, the bacteria were suspended in sterile water at an optical density (OD 600) of 0.2 (10^8^ CFU/mL).

For *Xoc* screenings, 4‐week‐old plants were inoculated by leaf infiltration, using a needleless syringe to introduce the bacterial suspension into the intercellular spaces of leaves (Reimers and Leach, 1991). One inoculation per leaf was performed in three leaves of the central tiller. Lesion lengths (in millimetres) were measured 12 days postinoculation (dpi), and 4 mm (the size of the infiltration site) was subtracted for each single measurement.

For *Xoo* screenings, 6‐week‐old plants were inoculated by the leaf clipping method (Kauffman *et al*., 1973), cutting approximately 4 cm from the tip of the two fully expanded leaves of the central tiller with scissors dipped in bacterial suspensions. Lesion lengths (in centimetres) were measured 14 dpi.

### Experimental design

For the S4 subset, four technical replicates of each MAGIC line were independently screened with each bacterial strain. The averages of the lesion lengths were calculated for each line and used for GWAS and IM.

For greenhouse studies of the S8 subset, separate experiments were conducted for each bacterial strain (treatment; *Xoc* BAI5, BLS256 and *Xoo* BAI3), using a randomized block design with three replications (blocked over time). For each experiment, the S8 subset MAGIC AILs (340 lines), founders and controls (Nipponbare and WAB 56‐125) were randomly planted. Least‐squares means (LS means) were calculated for each MAGIC line, using Proc Mixed (SAS Institute 2008). A random model (with ddfm = kr for degrees of freedom) was used, where lesion length was the response variable, and line and rep were the independent and blocking variables, respectively. The LS means were used for GWAS and IM.

For the AILs screened in the field, six technical replicates of each MAGIC line were independently screened with each bacterial strain. The averages of the lesion lengths were calculated and for each MAGIC line and used for GWAS and IM.

### Genomewide association studies

GWAS were performed using TASSEL (Bradbury *et al*., 2007). A kinship matrix (K) was generated for each SNP data set, and then, analyses were performed using the (Q + K) mixed linear model (MLM). To account for the false discovery rate, *q*‐values were calculated using the *q*‐value R package (Storey and Tibshirani, 2003). Manhattan plots were constructed using the qqman R package (Turner, 2014). For the S4 subset, TASSEL v 4.3.4 was used, and *P*‐values < 0.001 were considered significant (Tables S3 and S4); for the S8 subset, TASSEL v 5.0.2 was used, and three levels of significance were considered (Tables S3 and S5): *P‐value < 0.001 and *q*‐value > 0.05; *^*^ *P*‐value < 0.001 and *q*‐value < 0.05; *^**^ *P*‐value < 0.0001 and *q*‐value < 0.05.

### Interval mapping analyses

The R package mpMap (Huang and George, 2011), a platform that allows QTL mapping for multiparent populations, was used as described previously (Huang and George, 2011). SNP data for each subset of AILs and founders, as well as pedigree information of the AILs, were used to generate a linkage map (Raghavan *et al*., 2017). QTL were determined using simple interval mapping from the *mpIM* function, computing the founder effects between each pair of markers. For the S4 subset, mpMap v 1.14 was used (Figure S3), whereas for the S8 subset, mpMap v 2.0.2 was used (Figures 3 *,* S6 and S7). mpMap v 2.0.2 allowed the estimation of the percentage of phenotypic variation explained by each QTL.

### Prediction of SNP location and annotation

Functional annotation of variants was performed using SnpEff software (Cingolani *et al*., 2012) using the MSU7 rice reference annotation (Kawahara *et al*., 2013). Intergenic regions were defined as regions without predicted genes or located more than 1 kb upstream of genes.

### Linkage disequilibrium and haplotype analyses

Linkage disequilibrium (LD) and haplotype analyses were performed using Gevalt software (Davidovich *et al*., 2007), through the SNiPlay3 web application (Dereeper *et al*., 2015). Regions where multiple SNPs were significantly associated with resistance to several *X. oryzae* strains were defined on chrosomosomes 2, 4 and 11. The corresponding genotype data of the MAGIC population were then extracted and sent to Gevalt, which includes the visualization capabilities of Haploview (Barrett *et al*., 2005), for the construction of the LD patterns and haplotype blocks.

## Author contributions

A.M.B.‐C., C.R., O.K., G.M., H.L., V.V. and J.E.L. designed the research; A.M.B.‐C., C.T., E.E.D., K.D. and V.V. performed the research; A.M.B.‐C., C.R., A.D. and J.E.L. analysed the data; A.M.B.‐C. and J.E.L. wrote the manuscript.

## Supporting information


**Figure S1** Distribution of lesion length (cm) of MAGIC indica founders and S4 subset.
**Figure S2** Distribution of lesion length (cm) of MAGIC indica founders and S8 subset screened with multiple *Xoc* strains.
**Figure S3** Distribution of lesion length (cm) of MAGIC indica founders and S8 subset screened with multiple *Xoo* strains.
**Figure S4** QTL detection for resistance to *Xoc* MAI3 and *Xoo* BAI3 and MAI1 in MAGIC indica S4 subset.
**Figure S5** QTL detection for resistance to *Xoc* strains in MAGIC indica S8 subset.
**Figure S6** QTL detection for resistance to *Xoo* strains in MAGIC indica S8 subset.
**Figure S7** Complete haplotype block analyses and SNP effects in a hotspot region for *X. oryzae* resistance on chromosome 2.
**Figure S8** Complete haplotype block analyses and SNP effects in a hotspot region for *X. oryzae* resistance on chromosome 4.
**Figure S9** Complete haplotype block analyses and SNP effects in a hotspot region for *X. oryzae* resistance on chromosome 11.
**Table S1** Agronomic traits of MAGIC indica founders (modified from (Bandillo *et al*., 2013))
**Table S2** *Xanthomonas oryzae* strains used for inoculations.
**Table S3** SNP markers and MAGIC AILs used for GWAS and IM analyses in S4 and S8 subsets.
**Table S4** Significant SNPs in MAGIC indica S4 subset associated with disease resistance to African *Xoc* and *Xoo*, using MLM (*P*‐value < 0.001).
**Table S5** Significant SNPs in MAGIC indica S8 subset associated with disease resistance to African and Asian *Xoc* and *Xoo*, using MLM (*P*‐value < 0.001).
**Table S6** QTL detection for resistance to African *Xoc* and *Xoo* in MAGIC indica S4 subset (*P*‐value < 0.001), organized by chromosome.
**Table S7** QTL detection for resistance to African and Asian *Xoc* and *Xoo* in MAGIC indica S8 subset (*P*‐value < 0.001), organized by chromosome.
**Table S8** Comparison of QTL estimates for MAGIC indica S4 and S8 subsets for *Xoc* BAI5 and *Xoo* BAI3.
**Table S9** QTL effective to multiple *X. oryzae* strains found in this study.
**Table S10** Significantly associated SNP for resistance to multiple *X. oryzae* strains on chromosome 2, detected by GWAS in the MAGIC indica S8 subset.
**Table S11** Significantly associated SNP for resistance to multiple *X. oryzae* strains on chromosome 4, detected by GWAS in the MAGIC indica S8 subset.
**Table S12** Significantly associated SNP for resistance to multiple X. oryzae strains on chromosome 11, detected by GWAS in the MAGIC indica S8 subset.
