# Supplementary material for: Allelic variation for broad‐spectrum resistance and susceptibility to bacterial pathogens identified in a rice MAGIC population
Source: Plant Biotechnol J. 2018 Mar 8;16(9):1559–68. doi: 10.1111/pbi.12895 (PMC6097120; doi:10.1111/pbi.12895)
Supplement: Supplementary file 1 — Figure S1 Distribution of lesion length (cm) of MAGIC indica founders and S4 subset. Figure S2 Distribution of lesion length (cm) of MAGIC indica founders and S8 subset screened with multiple Xoc strains. Figure S3 Distribution of lesion length (cm) of MAGIC indica founders and S8 subset screened with multiple Xoo strains. Figure S4 QTL detection for resistance to Xoc MAI3 and Xoo BAI3 and MAI1 in MAGIC indica S4 subset. Figure S5 QTL detection for resistance to Xoc strains in MAGIC indica S8 subset. Figure S6 QTL detection for resistance to Xoo strains in MAGIC indica S8 subset. Figure S7 Complete haplotype block analyses and SNP effects in a hotspot region for X. oryzae resistance on chromosome 2. Figure S8 Complete haplotype block analyses and SNP effects in a hotspot region for X. oryzae resistance on chromosome 4. Figure S9 Complete haplotype block analyses and SNP effects in a hotspot region for X. oryzae resistance on chromosome 11. Table S1 Agronomic traits of MAGIC indica founders (modified from (Bandillo et al., 2013)) Table S2 Xanthomonas oryzae strains used for inoculations. Table S3 SNP markers and MAGIC AILs used for GWAS and IM analyses in S4 and S8 subsets. Table S4 Significant SNPs in MAGIC indica S4 subset associated with disease resistance to African Xoc and Xoo, using MLM (P‐value < 0.001). Table S5 Significant SNPs in MAGIC indica S8 subset associated with disease resistance to African and Asian Xoc and Xoo, using MLM (P‐value < 0.001). Table S6 QTL detection for resistance to African Xoc and Xoo in MAGIC indica S4 subset (P‐value < 0.001), organized by chromosome. Table S7 QTL detection for resistance to African and Asian Xoc and Xoo in MAGIC indica S8 subset (P‐value < 0.001), organized by chromosome. Table S8 Comparison of QTL estimates for MAGIC indica S4 and S8 subsets for Xoc BAI5 and Xoo BAI3. Table S9 QTL effective to multiple X. oryzae strains found in this study. Table S10 Significantly associated SNP for resistance to multiple X. oryz [file PBI-16-1559-s001.docx]

**Supporting Information**

**Allelic variation for broad-spectrum resistance and susceptibility to bacterial pathogens identified in a rice MAGIC population**

Ana M. Bossa-Castro ^a^, Cheick Tekete ^b,c^, Chitra Raghavan ^d,1^, Emily E. Delorean ^a,2^, Alexis Dereeper ^b^, Karim Dagno ^e^, Ousmane Koita ^c^, Gloria Mosquera ^f^, Hei Leung ^d^, Valérie Verdier ^b^, Jan E. Leach ^a^

**This file includes:**

Supporting information, Figures S1 to S9

Supporting information, Tables S1 to S12

**Supporting information, Figures**

**Figure S1.** Distribution of lesion length (cm) of MAGIC indica founders and S4 subset. (a) Screening of 171 AILs in growth chamber with *Xoc* BAI5. (b) Screening of 174 AILs in growth chamber with *Xoc* MAI3. (c) Screening of 152 AILs in the greenhouse with *Xoo* BAI3. (d) Screening of 137 AILs in the greenhouse with *Xoo* MAI1. Histograms represent mean values of MAGIC lines. Mean lesion lengths of founders are indicated with vertical lines (A: IR4630-22-2-5-1-3; B: Fedearroz 50; C: IR77298-14-1-2-10; D: Shan-Huang Zhan-2; E: PSBRc82; F: Sambha Mahsuri + Sub1; G: PSBRc158; H: IR45427-2B-2-2B-1-1).

**
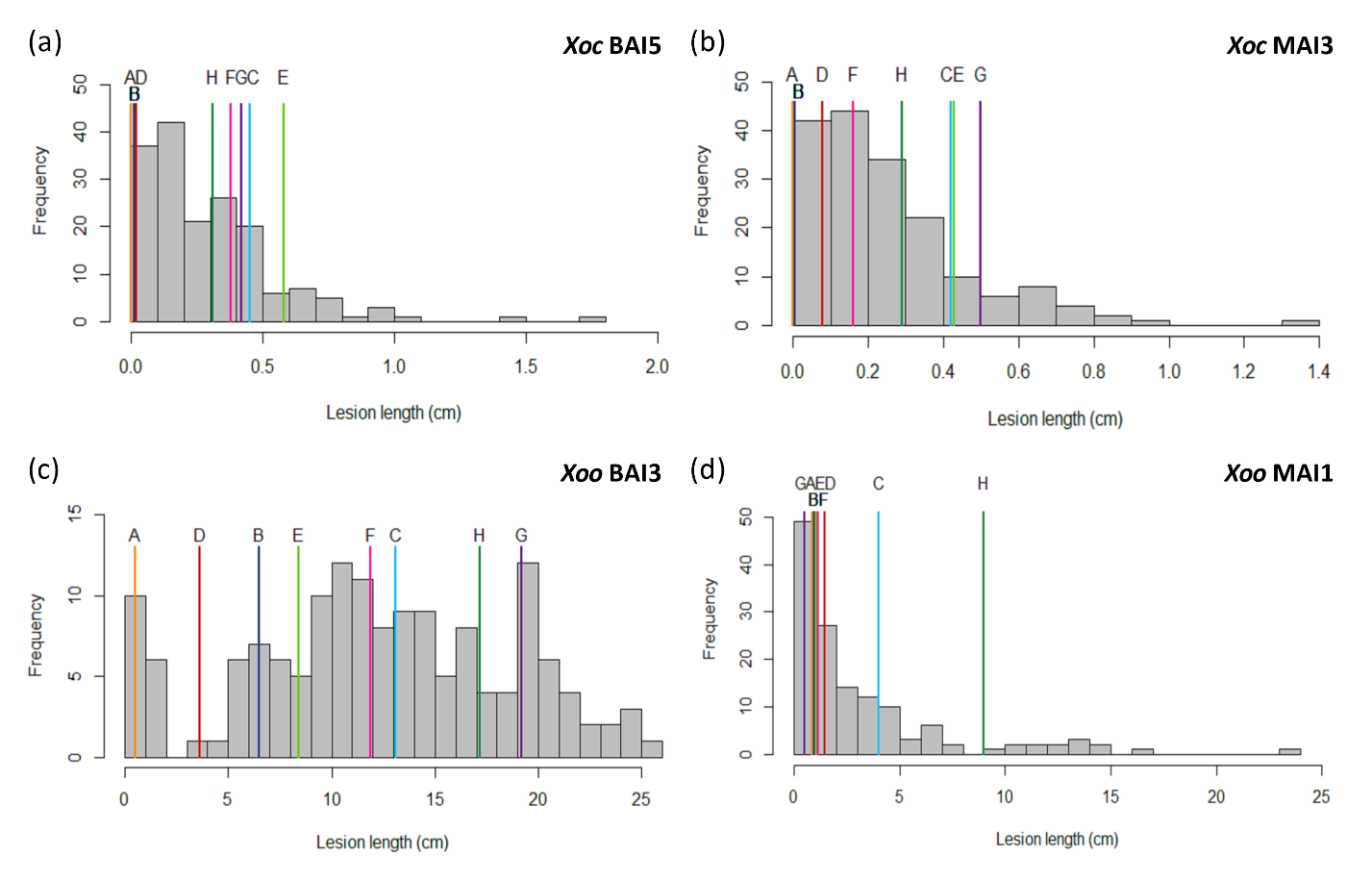
**

**Figure S2.** Distribution of lesion length (cm) of MAGIC indica founders and S8 subset screened with multiple *Xoc* strains. (a) Screening of 268 AILs with *Xoc* BLS256. (b) Screening of 290 AILs with *Xoc* MAI10. (c) Screening of 288 AILs with *Xoc* MAI46. (d) Screening of 270 AILs with *Xoc* MAI61. (e) Screening of 281 AILs with *Xoc* MAI67. (f) Screening of 280 AILs with *Xoc* MAI77. (g) Screening of 265 AILs with *Xoc* MAI123. (h) Screening of 252 AILs with *Xoc* MAI139. *Xoc* BLS256 was screened in the greenhouse, the rest of strains screened in the field. Histograms represent mean values of MAGIC lines. Mean lesion lengths of founders are indicated with vertical lines (A: IR4630-22-2-5-1-3; B: Fedearroz 50; C: IR77298-14-1-2-10; D: Shan-Huang Zhan-2; E: PSBRc82; F: Sambha Mahsuri + Sub1; G: PSBRc158; H: IR45427-2B-2-2B-1-1). For strains BLS256, MAI46, MAI61, MAI67 and MAI139 some phenotypic values of founders are missing due to lack of germination.

**
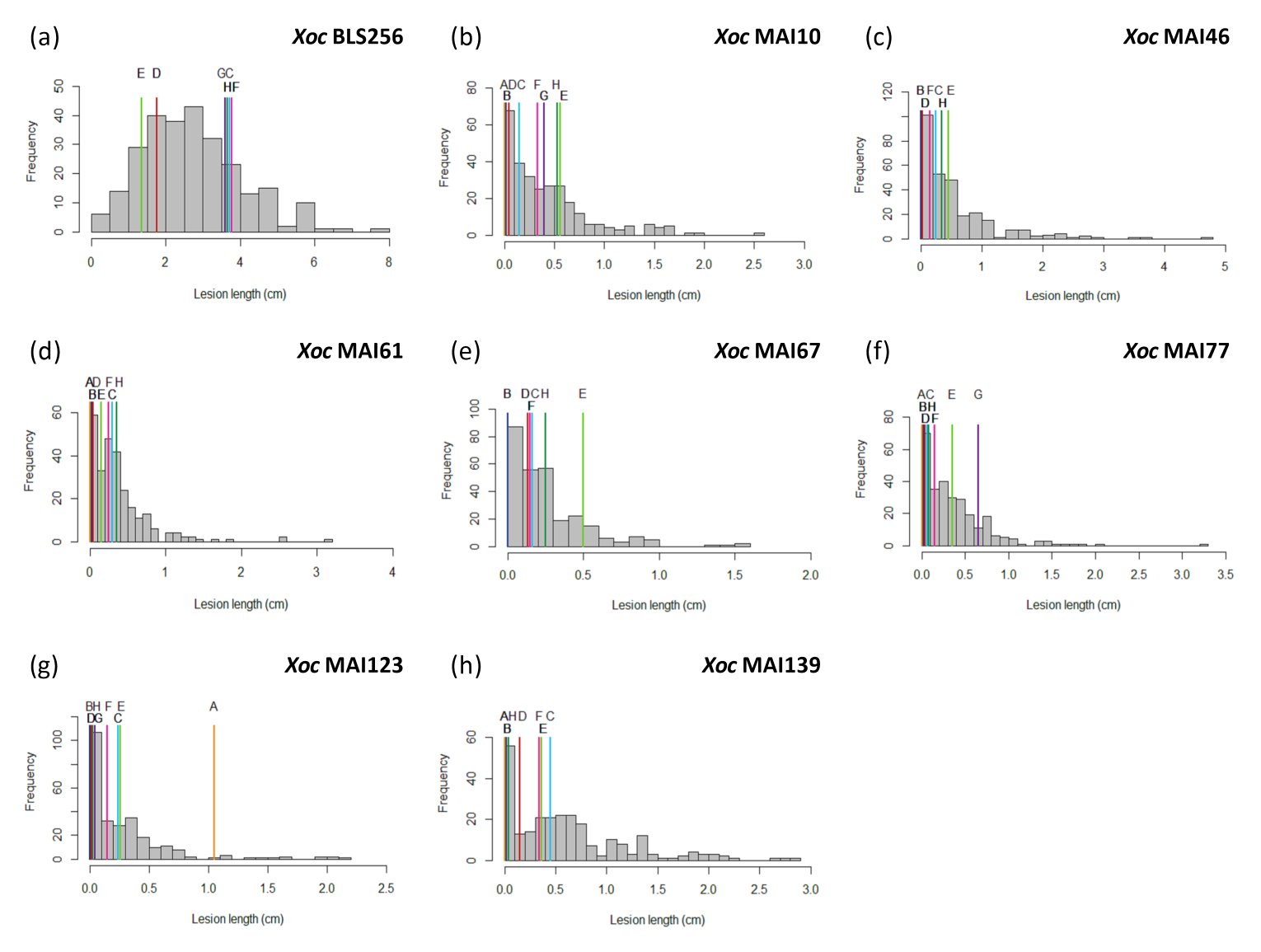
**

**Figure S3.** Distribution of lesion length (cm) of MAGIC indica founders and S8 subset screened with multiple *Xoo* strains. (a) Screening of 325 AILs with *Xoo* BAI3. (b) Screening of 281 AILs with *Xoo* CFBP1951. (c) Screening of 309 AILs with *Xoo* MAI70. (d) Screening of 296 AILs with *Xoo* MAI72. (e) Screening of 295 AILs with *Xoo* MAI93. (f) Screening of 270 AILs with *Xoo* MAI101. (g) Screening of 296 AILs with *Xoo* MAI130. (h) Screening of 276 AILs with *Xoo* MAI133. (i) Screening of 295 AILs with *Xoo* MAI134. (j) Screening of 286 AILs with *Xoo* MAI145. *Xoo* BAI3 was screened in the greenhouse, the rest of strains screened in the field. Histograms represent mean values of MAGIC lines. Mean lesion lengths of founders are indicated with vertical lines (A: IR4630-22-2-5-1-3; B: Fedearroz 50; C: IR77298-14-1-2-10; D: Shan-Huang Zhan-2; E: PSBRc82; F: Sambha Mahsuri + Sub1; G: PSBRc158; H: IR45427-2B-2-2B-1-1). For strains CFBP1951, MAI72, MAI93, MAI130, MAI133 and MAI134 some phenotypic values of founders are missing due to lack of germination.

**
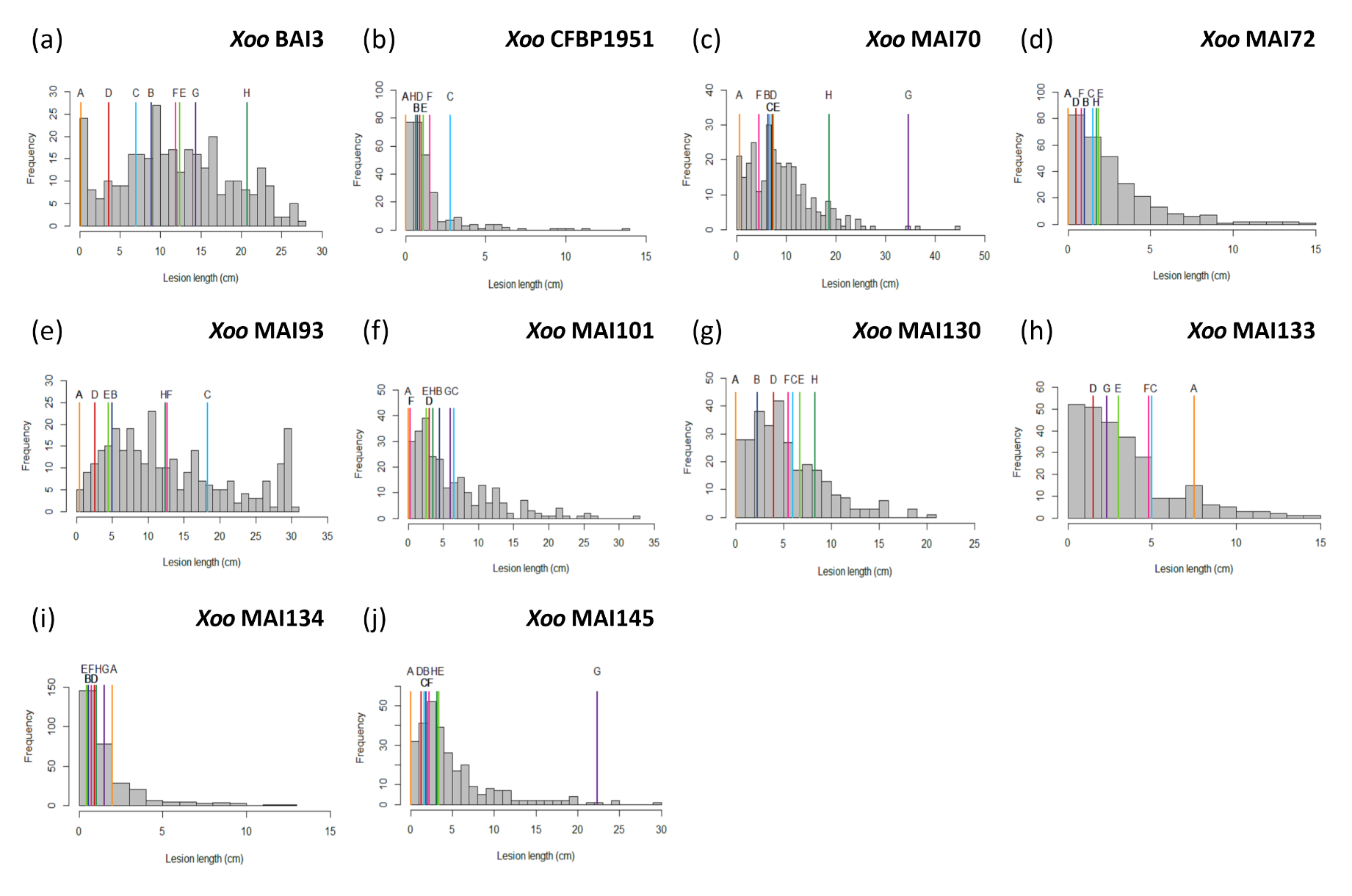
**

**Figure S4.** QTL detection for resistance to *Xoc* MAI3 and *Xoo* BAI3 and MAI1 in MAGIC indica S4 subset. (a, b) *Xoc* MAI3 (Markers=6,894). (c, d) *Xoo* BAI3 (Markers =7,390). (e, f) *Xoo* MAI1 (Markers =3,669). (a, c, e) Manhattan plots show the negative logarithm of the *P*-values for the mixed linear model, by chromosome. Solid blue line indicates significance threshold (*P*-value < 0.001). (b, d, f) Simple interval mapping showing chromosomes with significant QTL (p-value < 0.001). Green regions indicate 1-LOD support intervals.


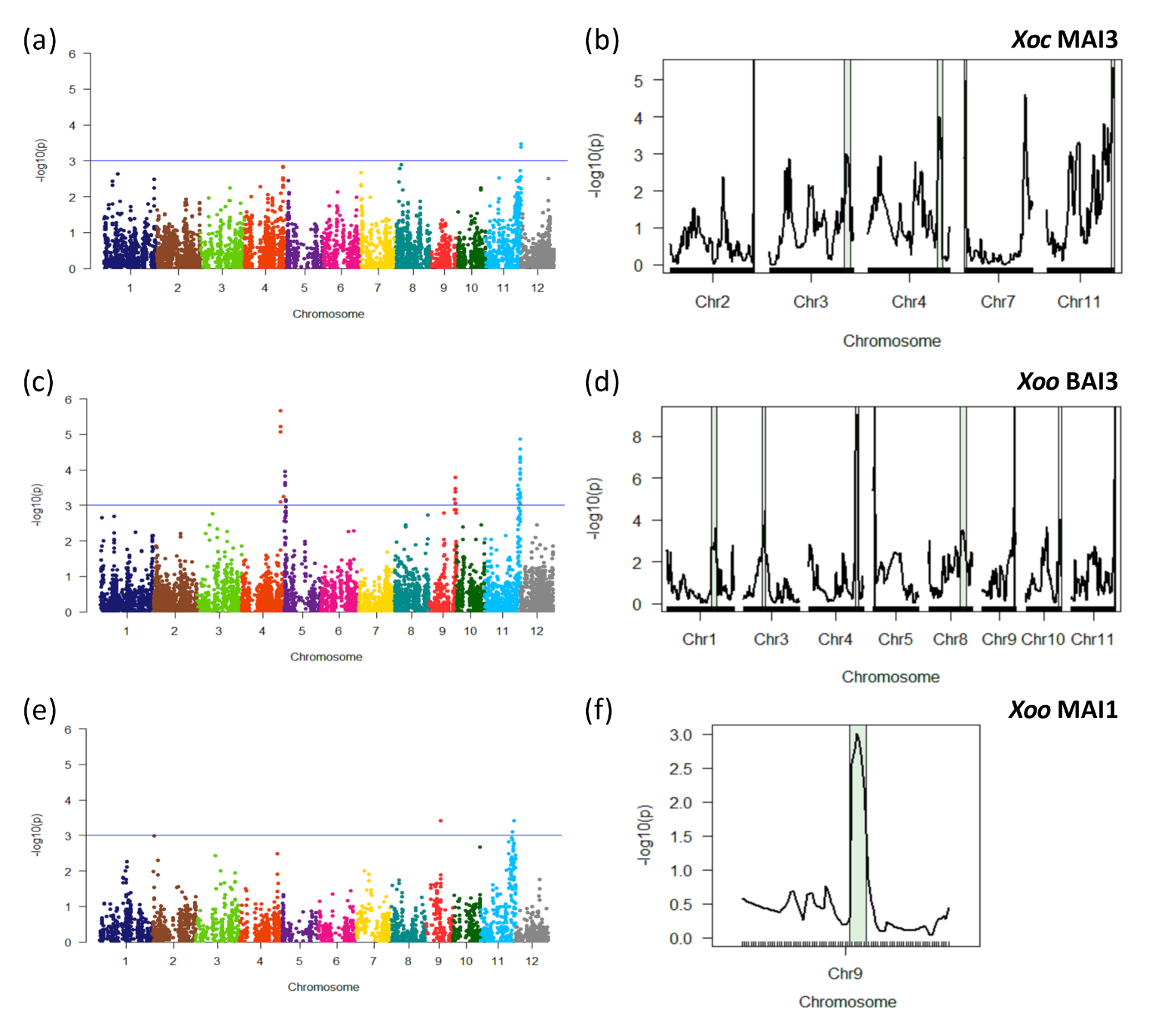


**Figure S5.** QTL detection for resistance to *Xoc* strains in MAGIC indica S8 subset. (a, b) *Xoc* BLS256; (c, d) *Xoc* MAI10; (e, f) *Xoc* MAI46; (g, h) *Xoc* MAI61; (i, j) *Xoc* MAI67; (k, l) *Xoc* MAI77; (m, n) *Xoc* MAI123; (o, p) *Xoc* MAI139. SNP markers used for all strains =14,475. (a, c, e, g, i, k, m, o). Manhattan plots show the negative logarithm of the *P*-values for the mixed linear model, by chromosome. Solid blue line indicates significance threshold (*P*-value < 0.001). (b, d, f, h, j, l, n, p) Simple interval mapping showing chromosomes with significant QTL (*P*-value < 0.001). Green regions indicate 1-LOD support intervals.

**
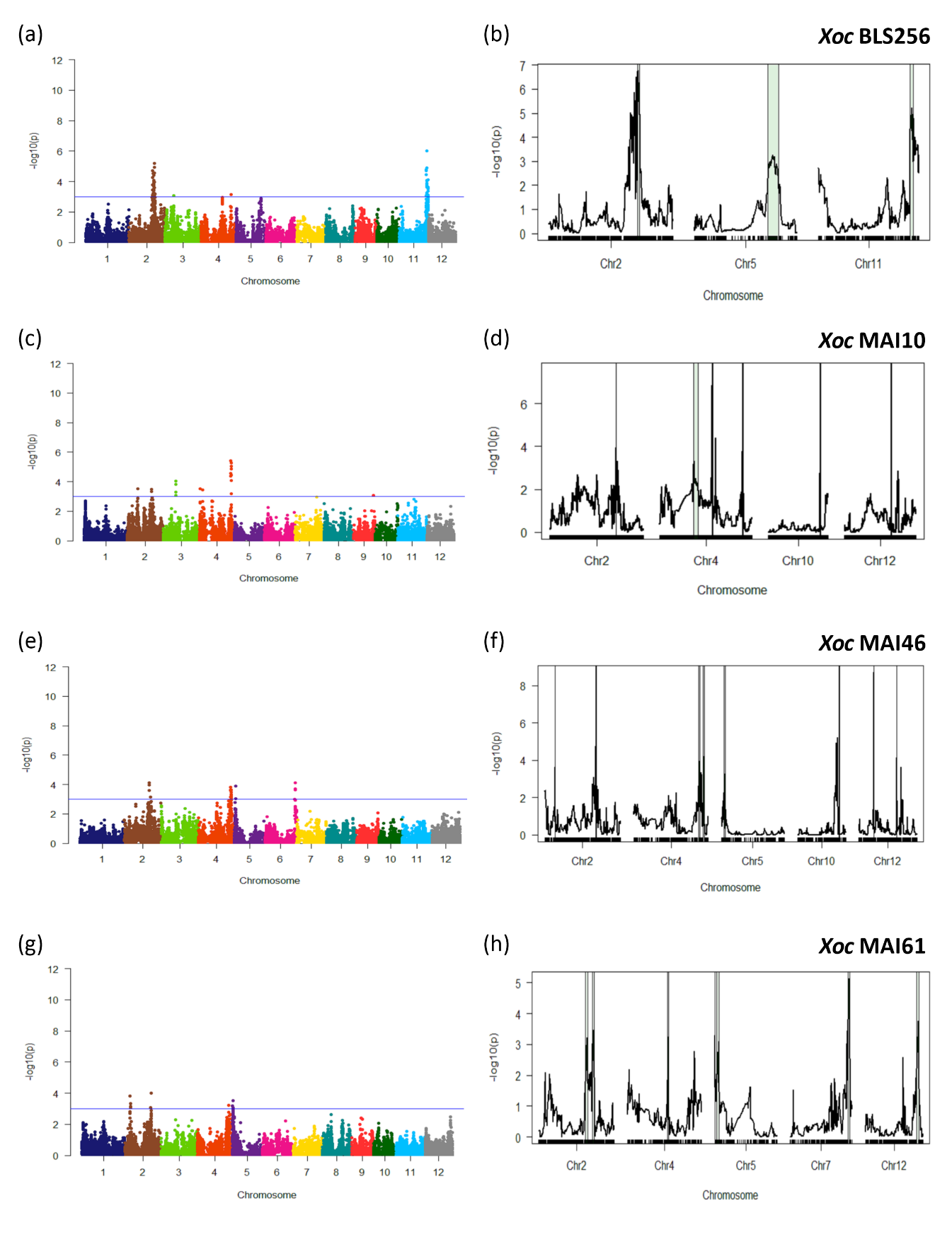
**

**Continuation Figure S5.**


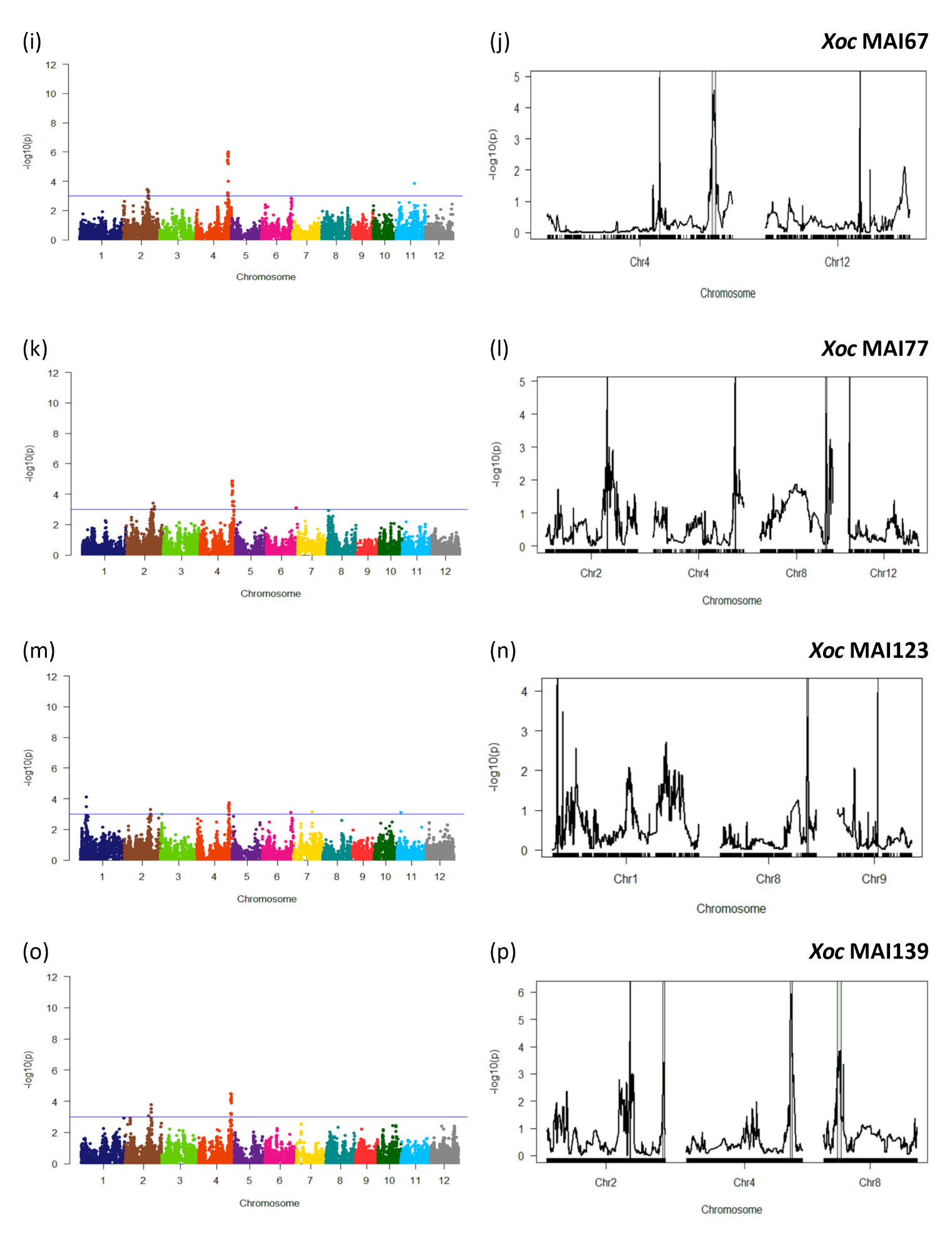


**Figure S6.** QTL detection for resistance to *Xoo* strains in MAGIC indica S8 subset. (a, b) *Xoo* BAI3; (c, d) *Xoo* CFBP1951; (e, f) *Xoo* MAI70, Note: first QTL on Chr 4 discarded (*P*-value > 0.001); (g, h) *Xoo* MAI72; (I, J) *Xoo* MAI93; (k, l) *Xoo* MAI101; (m, n) *Xoo* MAI130; (o, p) *Xoo* MAI133; (q, r) *Xoo* MAI134; (s, t) *Xoo* MAI145, Note: first QTL on Chr 9 discarded (*P*-value > 0.001). SNP markers used for all strains =14,475. (a, c, e, g, i, k, m, o, q, s). Manhattan plots show the negative logarithm of the *P*-values for the mixed linear model, by chromosome. Solid blue line indicates significance threshold (*P*-value < 0.001). (b, d, f, h, j, l, n, p, r, t) Simple interval mapping showing chromosomes with significant QTL (*P*-value < 0.001). Green regions indicate 1-LOD support intervals.
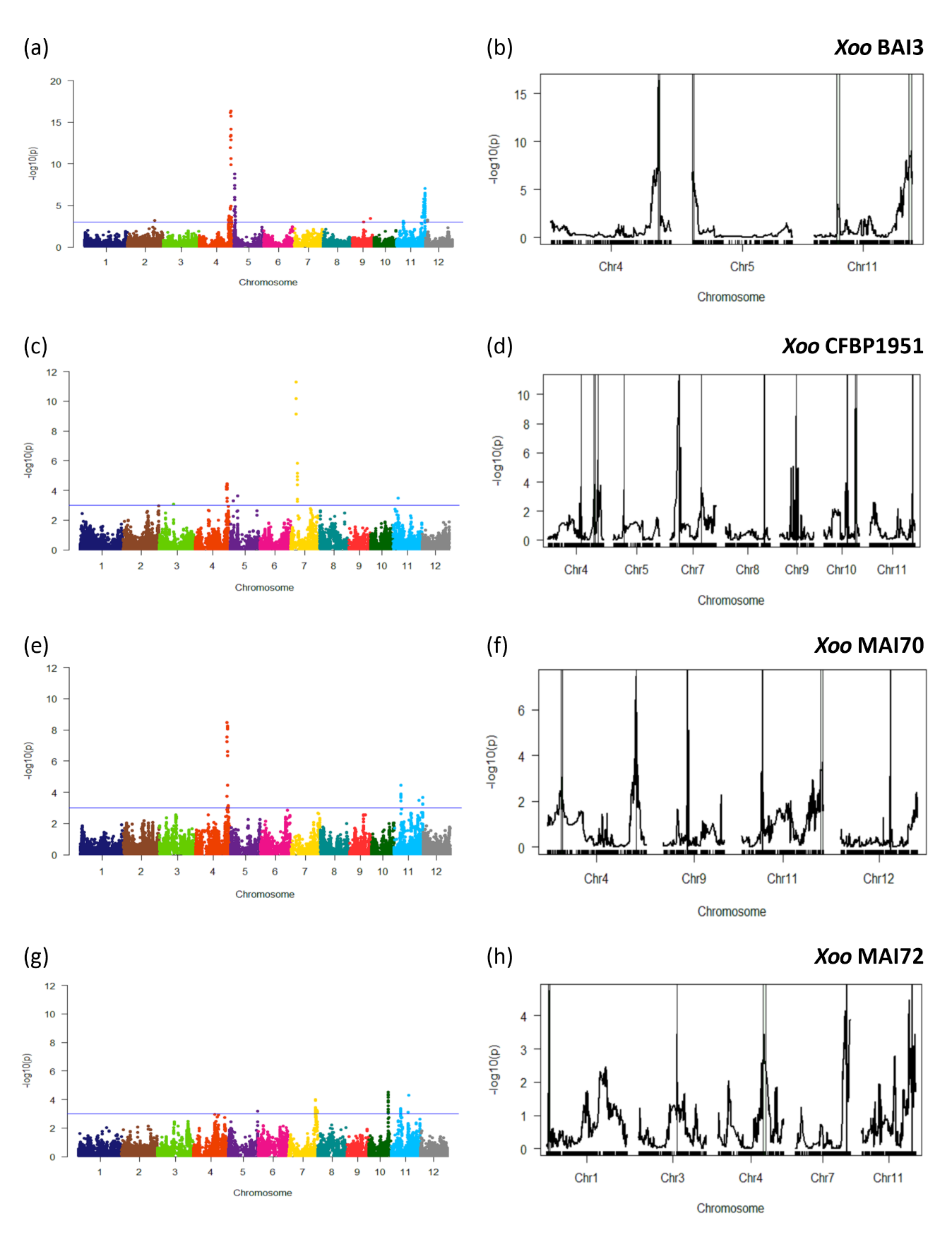


**Continuation Figure S6.**

**
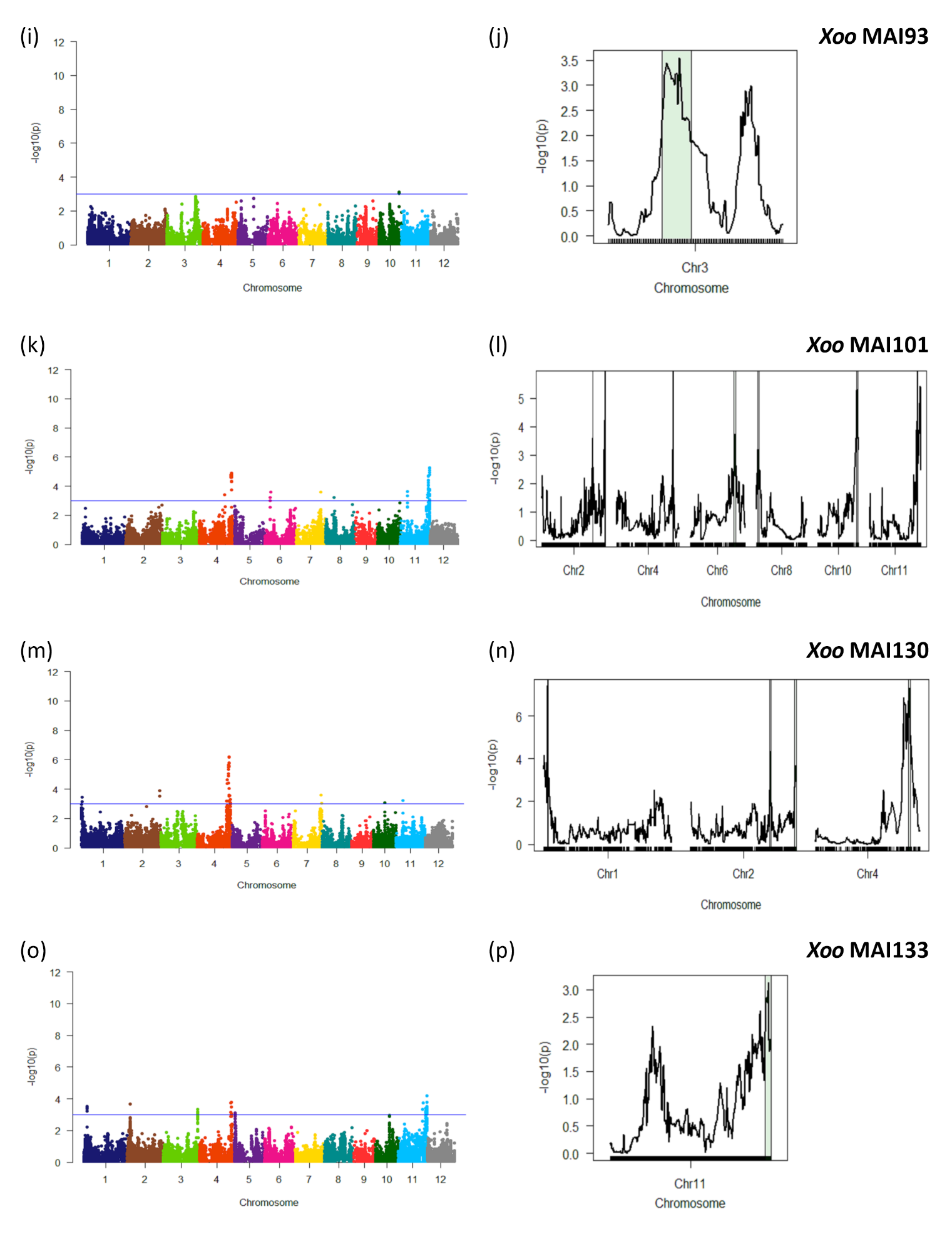
**

**Continuation Figure S6.**

**
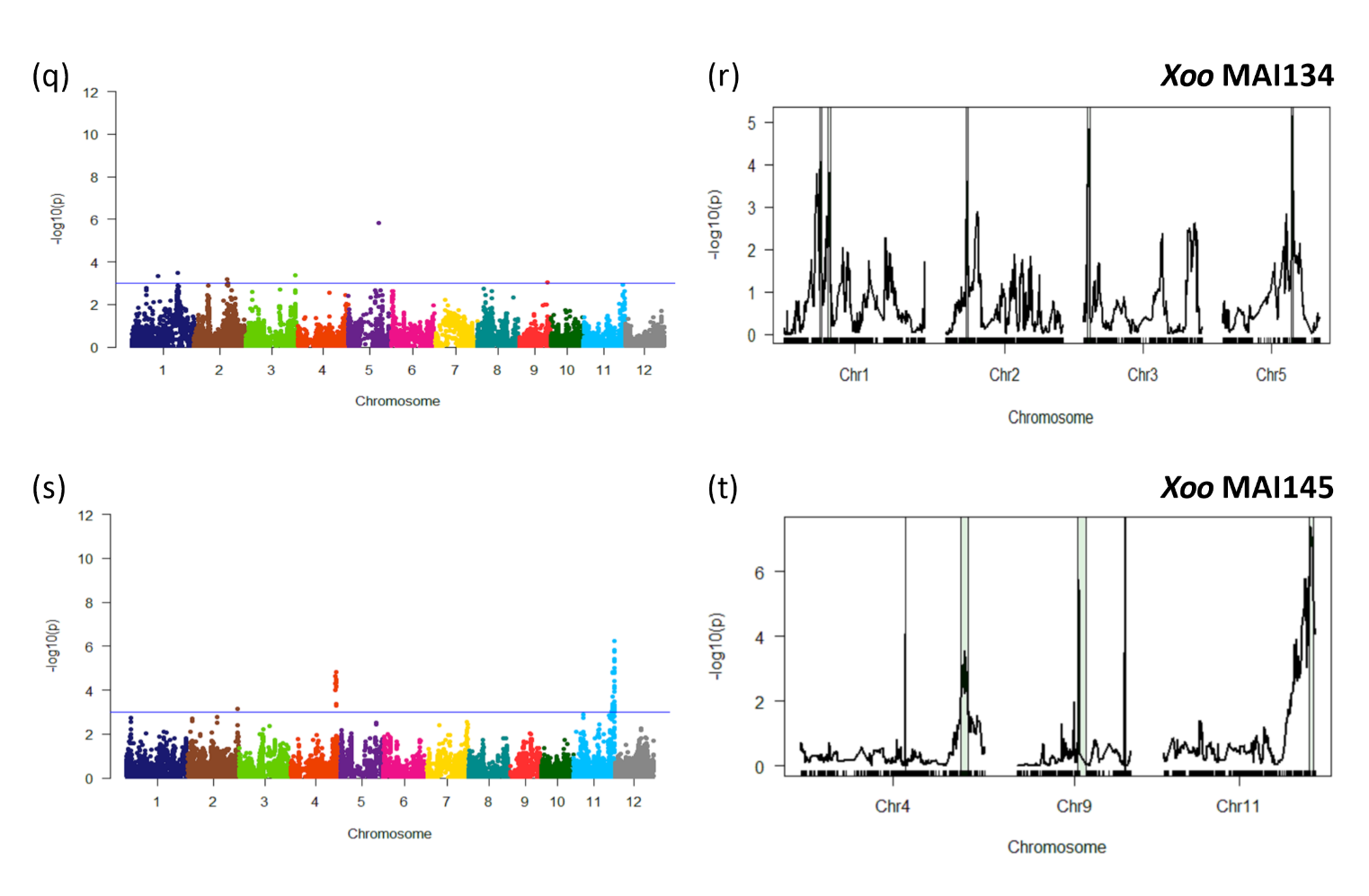
**

**Figure S7**. Complete haplotype block analyses and SNP effects in a hotspot region for *X. oryzae* resistance on chromosome 2. (a) Local Manhattan plot of *Xoc* BLS256 (top) and linkage disequilibrium (LD) with haplotype block analysis (bottom) of 24.5 – 27.2 Mbp region on chromosome 2. Red filled circles indicate SNPs that are significant to multiple for *X. oryzae* strains. LD heatmap shows the standard Haploview color scheme to display LD with bright red for strong LD (LOD = 2 D' = 1), pink (LOD = 2 D'<1), and blue (LOD <2 D' = 1) for intermediate LD, and white for no LD (LOD<2, D'<1). (b) Summary of SNP effects to *X. oryzae* strains on chromosome 2. Size of the bars denote the number of strains for which a SNP was significant (*P*-value < 0.001), color and direction of the bars indicate the sign of the estimated effect, either negative (left, orange) or positive (right, blue). A negative effect is associated with a more resistant phenotype (R), and a positive effect with a more susceptible phenotype (S). For each SNP the correspondent allele for the estimated effect is shown. SNP in bold denote the SNP was significant in both pathovars (*Xoc* and *Xoo*), otherwise the SNP was significant only for *Xoc* strains. The locus ID for each SNP (prefix “LOC_” is omitted), was predicted from the MSU7 rice reference annotation.

**
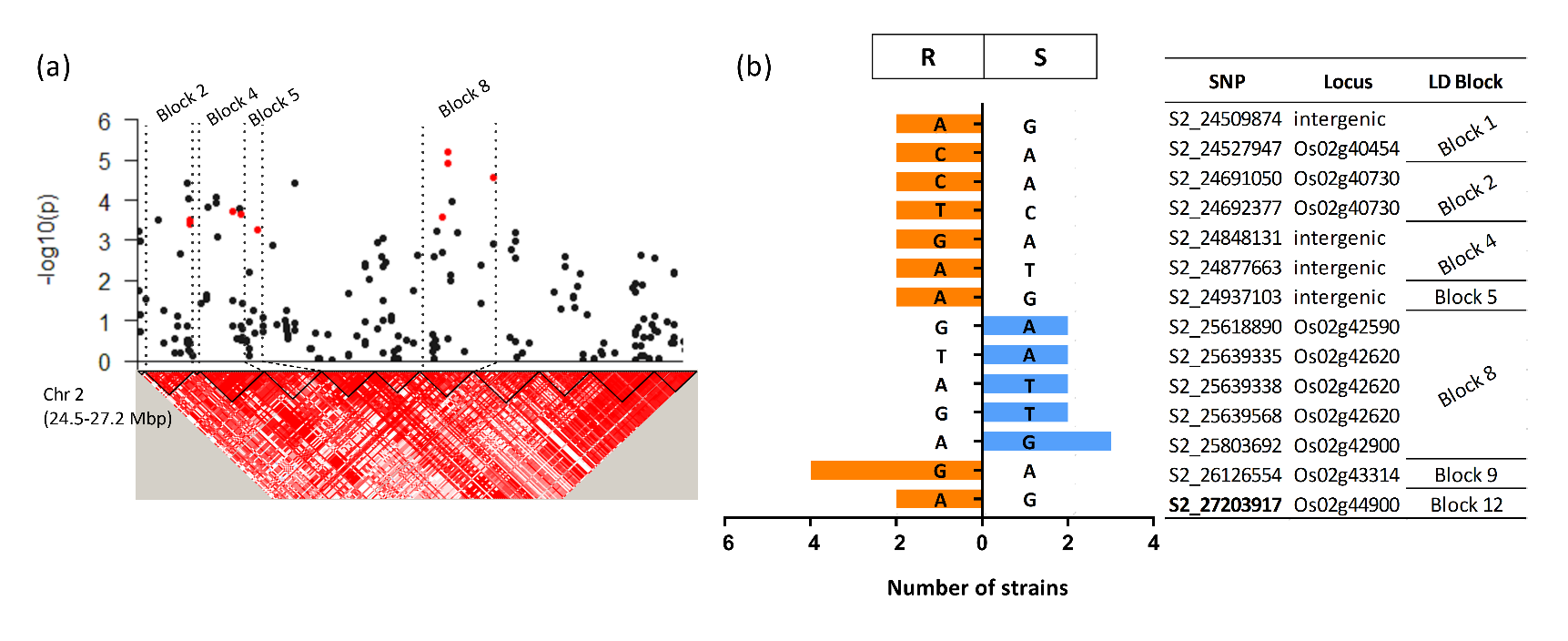
**

**Figure S8.** Complete haplotype block analyses and SNP effects in a hotspot region for *X. oryzae* resistance on chromosome 4. (a) Local Manhattan plot of *Xoc* BAI5 (top) and linkage disequilibrium (LD) with haplotype block analysis (bottom) of 29.5 – 32.6 Mbp region on chromosome 4. Red filled circles indicate SNPs that are significant to multiple *X. oryzae* strains. LD heatmap shows the standard Haploview color scheme to display LD with bright red for strong LD (LOD = 2 D' = 1), pink (LOD = 2 D'<1), and blue (LOD <2 D' = 1) for intermediate LD, and white for no LD (LOD<2, D'<1). (b) Summary of SNP effects to *X. oryzae* strains on chromosome 4. Size of the bars denote the number of strains for which a SNP was significant (*P*-value < 0.001), color and direction of the bars indicate the sign of the estimated effect, either negative (left, orange) or positive (right, blue). A negative effect is associated with a more resistant phenotype (R), and a positive effect with a more susceptible phenotype (S). For each SNP the correspondent allele for the estimated effect is shown. SNPs in bold denote the SNP was significant in both pathovars (*Xoc* and *Xoo*), otherwise the SNP was significant only for *Xoc* strains. The locus ID for each SNP (prefix “LOC_” is omitted), was predicted from the MSU7 rice reference annotation.


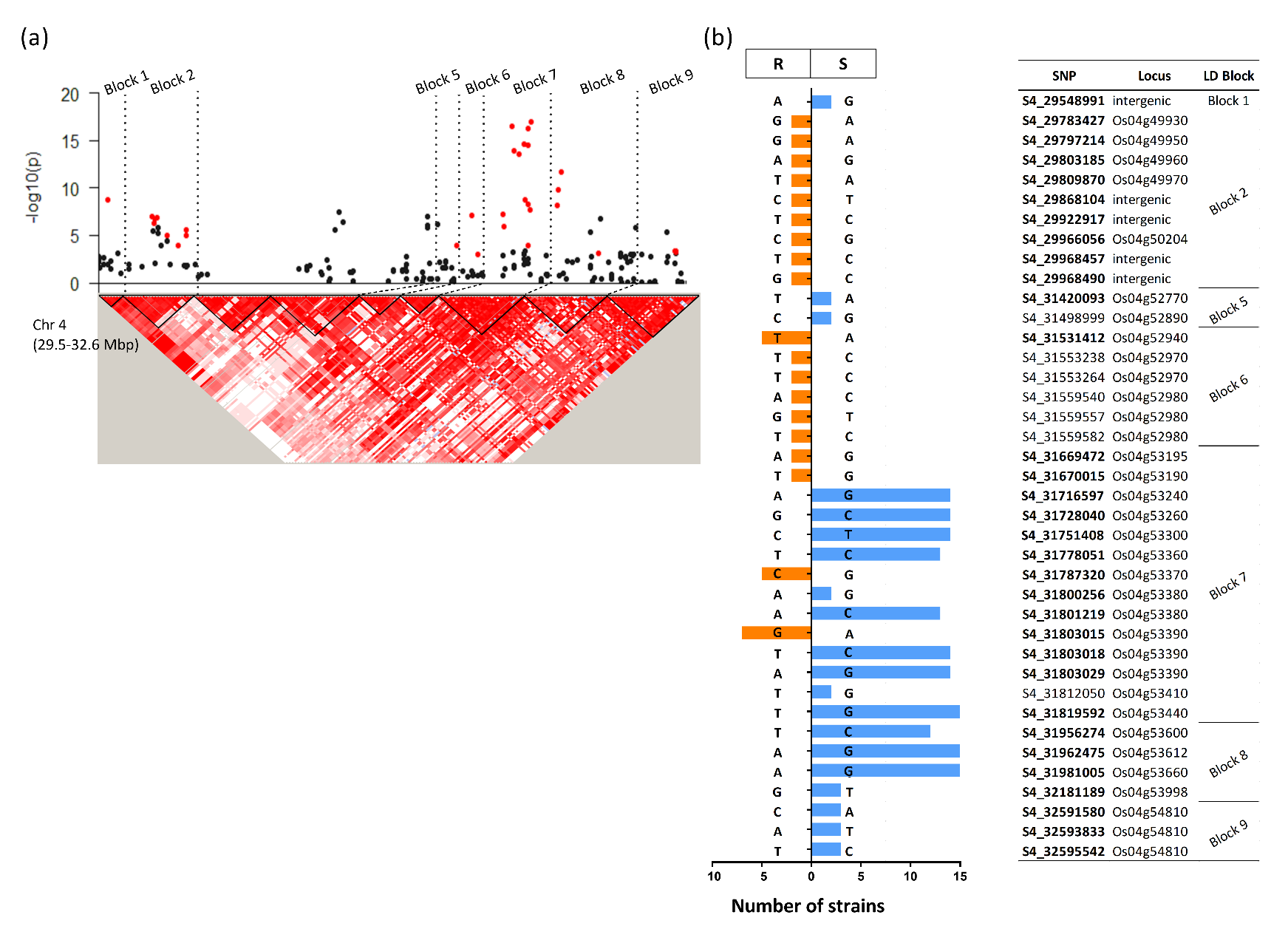


**Figure S9.** Complete haplotype block analyses and SNP effects in a hotspot region for *X. oryzae* resistance on chromosome 11. (a) Local Manhattan plot of *Xoo* BAI3 (top) and linkage disequilibrium (LD) with haplotype block analysis (bottom) of 25.8 – 28.9 Mbp region on chromosome 11. Red filled circles indicate SNPs that are significant to multiple for *X. oryzae* strains. LD heatmap shows the standard Haploview color scheme to display LD with bright red for strong LD (LOD = 2 D' = 1), pink (LOD = 2 D'<1), and blue (LOD <2 D' = 1) for intermediate LD, and white for no LD (LOD<2, D'<1). (b) Summary of SNP effects to *X. oryzae* strains on chromosome 11. Size of the bars denote the number of strains for which a SNP was significant (*P*-value < 0.001), color and direction of the bars indicate the sign of the estimated effect, either negative (left, orange) or positive (right, blue). A negative effect is associated with a more resistant phenotype (R), and a positive effect with a more susceptible phenotype (S). For each SNP the correspondent allele for the estimated effect is shown. SNPs in bold denote the SNP was significant in both pathovars (*Xoc* and *Xoo*), otherwise the SNP was significant only for *Xoo* strains. The locus ID for each SNP (prefix “LOC_” is omitted), was predicted from the MSU7 rice reference annotation.

**
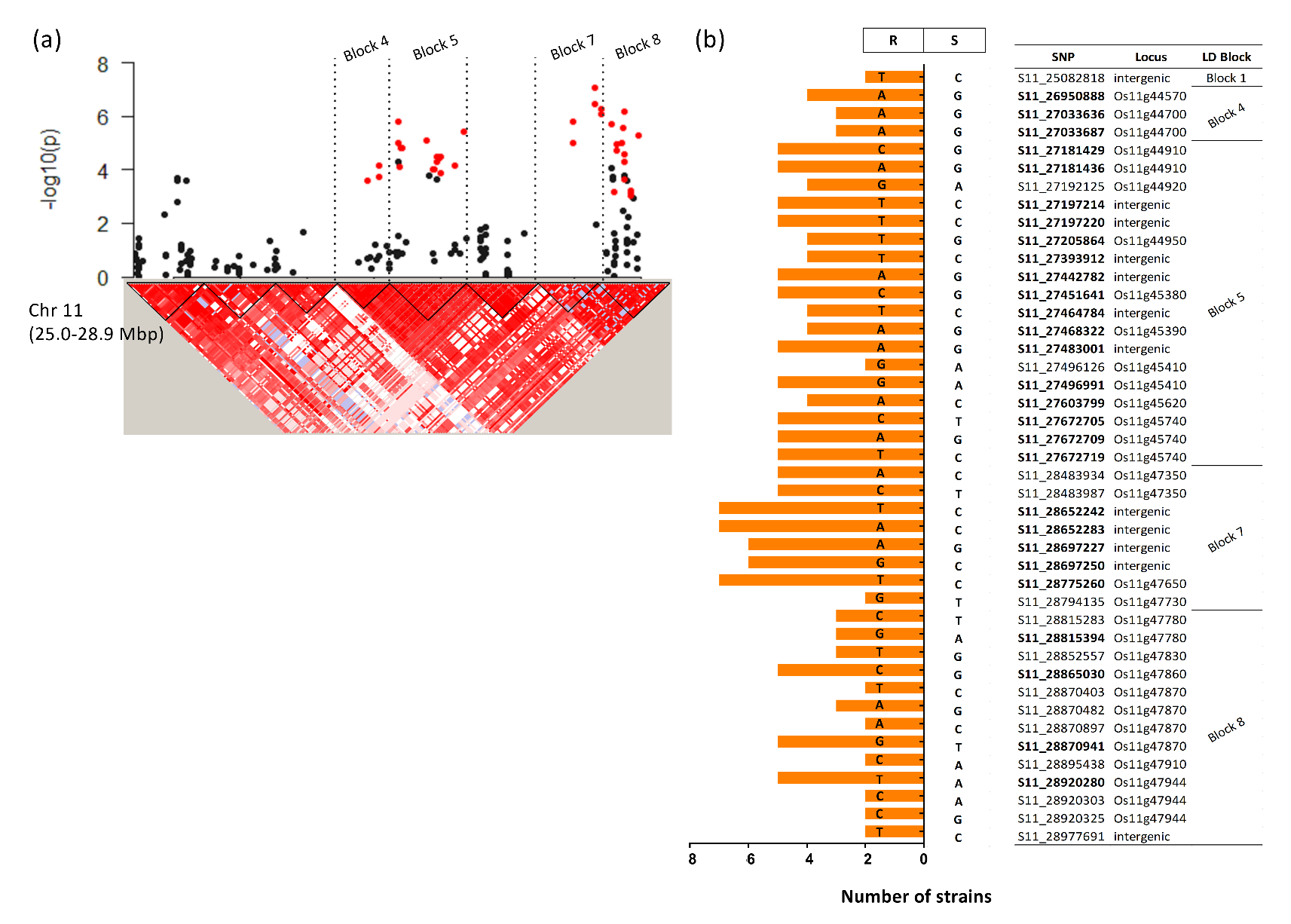
**

**Supporting information, Tables**

**Table S1**. Agronomic traits of MAGIC indica founders (modified from (Bandillo et al., 2013))

| **Designation** | **Germplasm/Variety** | **Origin** | **IRIS ID** | **GID†** | **Known**  ***R* genes** | **Agronomic relevance** |
| --- | --- | --- | --- | --- | --- | --- |
| A | IR4630-22-2-5-1-3 | IRRI | IRIS 313-15898 | 56023 | *Xa4* | Salt tolerance |
| B | Fedearroz 50 | Colombia | IRIS 313-15896 | 1846419 | *Xa4* | Delayed senescence, quality traits, disease tolerance, breeding progenitor |
| C | IR77298-14-1-2-10 | IRRI | IRIS 313-15901 | 2154106 | *Xa4* | Drought tolerance, tungro resistance, IR64 background |
| D | Shan-Huang Zhan-2 (SHZ-2) | China | IRIS 313-15897 | 402862 | *Xa4* | Blast resistance, high yielding, breeding progenitor in China |
| E | PSBRc82 (IRRI123 or IR64633-87-2-2-3-3) | IRRI | CX358 | 94801 | *Xa4, xa5* | High yielding, most popular variety of Philippines |
| F | Sambha Mahsuri + Sub1 (IR 07F287) | IRRI | IRIS 313-15900 | 2254836 |  | Mega variety, good grain quality, submergence tolerance |
| G | PSBRc 158 (IRRI146 or IR77186-122-2-2-3) | IRRI | IRIS 313-15902 | 1111266 | *Xa4* | High yielding in new plant type II (NPT) background |
| H | IR45427-2B-2-2B-1-1 | IRRI | IRIS 313-15899 | 1935108 | *Xa4* | Iron toxicity tolerance |

†GID, germplasm identification.

**Table S2**. *Xanthomonas oryzae* strains used for inoculations.

| **Pathovar** | **Strain** | **Origin** | **Region - Site** | **Race** | **XopAJ (avrXo1)** | **Screening** | **Screening site** | **Reference** |
| --- | --- | --- | --- | --- | --- | --- | --- | --- |
| *Xoc* | BAI5 | Burkina Faso | Hauts Bassins - Vallée du Kou |  | - | S4, S8 | Growth chamber, greenhouse | (Wonni et al., 2014) |
| *Xoc* | BLS256 | Philippines | Los Baños |  | + | S8 | Greenhouse | (Bogdanove et al., 2011‡) |
| *Xoc* | MAI3 | Mali | O. Niger - Niono |  | + | S4 | Growth chamber | (Gonzalez et al., 2007) |
| *Xoc* | MAI10 | Mali | O. Niger - Niono |  | - | S8 | Field | (Gonzalez et al., 2007) |
| *Xoc* | MAI46 | Mali | Koulikoro - Baguineda |  | + | S8 | Field | (Wonni et al., 2014) |
| *Xoc* | MAI61 | Mali | O.Niger - Madina |  | + | S8 | Field | unpublished |
| *Xoc* | MAI67 | Mali | O. Niger - Niono |  | + | S8 | Field | unpublished |
| *Xoc* | MAI77 | Mali | Sikasso - Sélingué |  | + | S8 | Field | unpublished |
| *Xoc* | MAI123 | Mali | O.Niger - Bewani |  | + | S8 | Field | unpublished |
| *Xoc* | MAI139 | Mali | O.Niger Kogoni |  | + | S8 | Field | unpublished |
|  |  |  |  |  |  |  |  |  |
| *Xoo* | BAI3 | Burkina Faso | East center - Bagre | A1 |  | S4, S8 | Greenhouse | (Gonzalez et al., 2007) |
| *Xoo* | CFBP1951 | Mali | Kayes | A3 |  | S8 | Field | unpublished |
| *Xoo* | MAI1 | Mali | O. Niger - Niono | A3 |  | S4 | Greenhouse | (Gonzalez et al., 2007) |
| *Xoo* | MAI70 | Mali | O. Niger - Niono | A6 |  | S8 | Field | unpublished |
| *Xoo* | MAI72 | Mali | O. Niger - Niono | A5 |  | S8 | Field | unpublished |
| *Xoo* | MAI93 | Mali | O. Niger - Niono | A7 |  | S8 | Field | unpublished |
| *Xoo* | MAI101 | Mali | O. Niger - Niono | A4 |  | S8 | Field | unpublished |
| *Xoo* | MAI130 | Mali | O. Niger - Bewani 2 | A9 |  | S8 | Field | unpublished |
| *Xoo* | MAI133 | Mali | O. Niger - Bewani 10 | A9 |  | S8 | Field | unpublished |
| *Xoo* | MAI134 | Mali | O. Niger - Kala 3 | A3 |  | S8 | Field | unpublished |
| *Xoo* | MAI136 | Mali | O. Niger - Kala 9 | A8 |  | S8 | Field | unpublished |
| *Xoo* | MAI145 | Mali | O. Niger - Kouroumari | A6 |  | S8 | Field | unpublished |

‡ Bogdanove, A.J., Koebnik, R., Lu, H., Furutani, A., Angiuoli, S.V. *et al*. (2011) Two new complete genome sequences offer insight into host and tissue specificity of plant pathogenic *Xanthomonas* spp*. J Bacteriol* **193**, 5450-5464.

**Table S3.** SNP markers and MAGIC AILs used for GWAS and IM analyses in S4 and S8 subsets.

| **MAGIC subset** | **Pathovar** | **Strain** | **N° AILs screened** | **Initial SNP dataset** | **Final dataset used** |
| --- | --- | --- | --- | --- | --- |
| S4 | *Xoc* | BAI5 | 171 | 634,103 | 7,258 |
|  | *Xoc* | MAI3 | 174 |  | 6,894 |
|  |  |  |  |  |  |
|  | *Xoo* | BAI3 | 152 |  | 7,390 |
|  | *Xoo* | MAI1 | 137 |  | 3,669 |
|  |  |  |  |  |  |
| S8 | *Xoc* | BAI5 | 323 | 396,361 | 14,475 |
|  | *Xoc* | BLS256 | 268 |  |  |
|  | *Xoc* | MAI10 | 290 |  |  |
|  | *Xoc* | MAI46 | 288 |  |  |
|  | *Xoc* | MAI61 | 270 |  |  |
|  | *Xoc* | MAI67 | 281 |  |  |
|  | *Xoc* | MAI77 | 280 |  |  |
|  | *Xoc* | MAI123 | 265 |  |  |
|  | *Xoc* | MAI139 | 252 |  |  |
|  |  |  |  |  |  |
|  | *Xoo* | BAI3 | 325 |  |  |
|  | *Xoo* | CFBP1951 | 281 |  |  |
|  | *Xoo* | MAI70 | 309 |  |  |
|  | *Xoo* | MAI72 | 296 |  |  |
|  | *Xoo* | MAI93 | 295 |  |  |
|  | *Xoo* | MAI101 | 270 |  |  |
|  | *Xoo* | MAI130 | 296 |  |  |
|  | *Xoo* | MAI133 | 276 |  |  |
|  | *Xoo* | MAI134 | 295 |  |  |
|  | *Xoo* | MAI136 | 276 |  |  |
|  | *Xoo* | MAI145 | 286 |  |  |

**Table S4**. Significant SNPs in MAGIC indica S4 subset associated with disease resistance to African *Xoc* and *Xoo*, using MLM (*P*-value < 0.001). Effect estimate corresponds to the phenotypic estimated deviation from the mean of the effect allele at each SNP (a negative effect indicates shorter lesion lengths than the mean and a positive effects indicates the opposite). Effect estimate is calculated in millimeters (mm) for *Xoc* strains and centimeters (cm) for *Xoo* strains. Stars (*) in significance correspondence panel indicate level of significance for GWAS (*P*-value < 0.001 and q-value > 0.05).

| **Pathovar** | **Strain** | **Marker** | **p-value** | **q-value** | **R^2^** | **Effect** | **Effect** | **AILs with Effect** | **Significance** |
| --- | --- | --- | --- | --- | --- | --- | --- | --- | --- |
|  |  | **(Chr_Pos)** |  |  |  | **estimate** | **allele/Null** | **allele/Null** | **correspondence** |
| *Xoc* | BAI5 | S4_31266738 | 8.4E-04 | 6.9E-02 | 6.9 | 2.7 | A/G | 153/13 | * |
| *Xoo* | BAI3 | S4_31716597 | 8.6E-06 | 1.5E-01 | 15.5 | 9.5 | G/A | 131/8 | * |
| *Xoo* | BAI3 | S4_31751408 | 6.1E-06 | 1.6E-01 | 16.3 | 10.0 | T/C | 132/8 | * |
| *Xoo* | BAI3 | S4_31779189 | 8.1E-04 | 8.6E-02 | 8.6 | 5.2 | C/T | 122/15 | * |
| *Xoo* | BAI3 | S4_31803018 | 2.2E-06 | 1.8E-01 | 18.0 | 10.6 | C/T | 128/8 | * |
| *Xoo* | BAI3 | S4_34659203 | 5.6E-04 | 9.0E-02 | 9.0 | 7.4 | G/T | 139/8 | * |
|  |  |  |  |  |  |  |  |  |  |
| *Xoo* | BAI3 | S5_227187 | 2.3E-04 | 1.1E-01 | 10.9 | 7.3 | C/A | 118/12 | * |
| *Xoo* | BAI3 | S5_285834 | 2.3E-04 | 1.1E-01 | 10.7 | 7.3 | G/A | 129/12 | * |
| *Xoo* | BAI3 | S5_353165 | 1.1E-04 | 1.1E-01 | 11.4 | 6.8 | C/T | 126/15 | * |
| *Xoo* | BAI3 | S5_440644 | 1.5E-04 | 1.0E-01 | 10.4 | 6.7 | G/A | 127/15 | * |
| *Xoo* | BAI3 | S5_759048 | 2.7E-04 | 1.0E-01 | 10.2 | -5.3 | C/T | 26/108 | * |
| *Xoo* | BAI3 | S5_849560 | 7.0E-04 | 9.7E-02 | 9.7 | 6.2 | C/T | 105/17 | * |
| *Xoo* | BAI3 | S5_1200961 | 7.5E-04 | 8.4E-02 | 8.4 | 5.7 | G/A | 123/20 | * |
| *Xoc* | BAI5 | S5_1829611 | 1.2E-04 | 1.0E-01 | 10.2 | -2.1 | C/A | 111/33 | * |
| *Xoc* | BAI5 | S5_1833139 | 2.4E-04 | 8.7E-02 | 8.7 | -1.9 | T/A | 119/35 | * |
|  |  |  |  |  |  |  |  |  |  |
| *Xoc* | BAI5 | S8_5250168 | 4.1E-04 | 8.0E-02 | 8.0 | -3.2 | C/T | 152/9 | * |
| *Xoc* | BAI5 | S8_5469156 | 6.4E-04 | 7.5E-02 | 7.5 | -2.0 | C/T | 118/34 | * |
|  |  |  |  |  |  |  |  |  |  |
| *Xoo* | MAI1 | S9_12266970 | 3.9E-04 | 1.1E-01 | 10.9 | -6.2 | G/C | 121/7 | * |
| *Xoo* | BAI3 | S9_21002076 | 6.6E-04 | 9.7E-02 | 9.7 | 4.2 | G/A | 57/68 | * |
| *Xoo* | BAI3 | S9_21176469 | 8.8E-04 | 8.8E-02 | 8.8 | -7.6 | G/T | 123/9 | * |
| *Xoo* | BAI3 | S9_21243301 | 4.2E-04 | 1.1E-01 | 11.0 | 5.7 | T/C | 26/96 | * |
| *Xoo* | BAI3 | S9_21291790 | 3.4E-04 | 1.2E-01 | 12.0 | 5.4 | T/C | 26/102 | * |
| *Xoo* | BAI3 | S9_21291867 | 1.6E-04 | 1.2E-01 | 12.4 | 5.9 | A/C | 27/104 | * |

**Continuation Table S4**.

| **Pathovar** | **Strain** | **Marker** | **p-value** | **q-value** | **R^2^** | **Effect** | **Effect** | **AILs with Effect** | **Significance** |
| --- | --- | --- | --- | --- | --- | --- | --- | --- | --- |
|  |  | **(Chr_Pos)** |  |  |  | **estimate** | **allele/Null** | **allele/Null** | **correspondence** |
| *Xoo* | MAI1 | S11_26195519 | 8.1E-04 | 1.0E-01 | 10.0 | -4.3 | C/A | 114/12 | * |
| *Xoo* | BAI3 | S11_27412501 | 4.8E-04 | 9.0E-02 | 9.0 | 6.1 | C/G | 16/120 | * |
| *Xoo* | BAI3 | S11_27412530 | 6.4E-04 | 8.7E-02 | 8.7 | 6.0 | C/T | 16/119 | * |
| *Xoo* | BAI3 | S11_27437727 | 2.7E-04 | 1.0E-01 | 10.5 | 6.0 | G/A | 19/113 | * |
| *Xoo* | MAI1 | S11_27451641 | 3.8E-04 | 1.2E-01 | 11.9 | -4.0 | C/G | 103/17 | * |
| *Xoo* | BAI3 | S11_27677943 | 3.4E-04 | 1.0E-01 | 10.2 | -7.7 | A/G | 124/11 | * |
| *Xoo* | BAI3 | S11_27677963 | 3.4E-04 | 1.0E-01 | 10.2 | -7.7 | T/C | 124/11 | * |
| *Xoo* | BAI3 | S11_28652283 | 8.1E-04 | 8.9E-02 | 8.9 | -5.1 | A/C | 112/25 | * |
| *Xoo* | BAI3 | S11_28697227 | 1.8E-04 | 1.1E-01 | 11.5 | 5.5 | G/A | 27/104 | * |
| *Xoo* | BAI3 | S11_28733787 | 4.4E-04 | 9.4E-02 | 9.4 | -7.1 | C/A | 130/13 | * |
| *Xoo* | BAI3 | S11_28760878 | 1.8E-04 | 1.3E-01 | 13.5 | -6.0 | C/T | 96/25 | * |
| *Xoo* | BAI3 | S11_28793592 | 2.5E-05 | 1.5E-01 | 15.1 | -9.3 | C/T | 110/11 | * |
| *Xoo* | BAI3 | S11_28807359 | 5.1E-04 | 8.8E-02 | 8.8 | -7.2 | G/A | 137/12 | * |
| *Xoo* | BAI3 | S11_28809570 | 1.4E-05 | 1.6E-01 | 15.8 | -8.7 | C/G | 124/13 | * |
| *Xoo* | BAI3 | S11_28809652 | 4.9E-05 | 1.2E-01 | 11.9 | -7.8 | C/G | 133/14 | * |
| *Xoo* | BAI3 | S11_28809654 | 4.8E-05 | 1.2E-01 | 11.9 | -7.9 | A/G | 132/14 | * |
| *Xoo* | BAI3 | S11_28865030 | 9.2E-05 | 1.2E-01 | 11.8 | -5.9 | C/G | 112/27 | * |
| *Xoo* | BAI3 | S11_28870403 | 1.2E-04 | 1.2E-01 | 12.0 | -5.7 | T/C | 105/26 | * |
| *Xoo* | BAI3 | S11_28870482 | 4.4E-05 | 1.5E-01 | 14.7 | -6.4 | A/G | 99/24 | * |
| *Xoo* | BAI3 | S11_28870897 | 6.0E-04 | 9.1E-02 | 9.1 | -5.2 | A/C | 108/24 | * |
| *Xoo* | BAI3 | S11_28870941 | 6.0E-05 | 1.3E-01 | 13.2 | -6.2 | G/T | 107/25 | * |
| *Xoc* | MAI3 | S11_28871165 | 4.3E-04 | 8.5E-02 | 8.5 | -2.6 | G/C | 147/13 | * |
| *Xoo* | BAI3 | S11_28871228 | 9.1E-04 | 9.4E-02 | 9.4 | 5.0 | C/G | 25/99 | * |
| *Xoo* | BAI3 | S11_28896330 | 1.8E-04 | 1.0E-01 | 10.5 | -8.0 | A/G | 137/11 | * |
| *Xoo* | BAI3 | S11_28896471 | 1.3E-04 | 1.2E-01 | 12.4 | -7.4 | G/A | 110/14 | * |
| *Xoo* | BAI3 | S11_28920280 | 5.3E-04 | 1.0E-01 | 10.3 | 5.6 | A/T | 23/104 | * |
| *Xoc* | MAI3 | S11_28920303 | 3.3E-04 | 9.1E-02 | 9.1 | -2.5 | C/A | 140/14 | * |

**Table S5**. Significant SNPs in MAGIC indica S8 subset associated with disease resistance to African and Asian *Xoc* and *Xoo*, using MLM (*P*-value < 0.001). Effect estimate corresponds to the phenotypic estimated deviation from the mean of the effect allele at each SNP (a negative effect indicates shorter lesion lengths than the mean and a positive effects indicates the opposite). Effect estimate is calculated in millimeters (mm) for *Xoc* strains and centimeters (cm) for *Xoo* strains. Stars in significance correspondence panel indicate level of significance of GWAS markers as follows: (*) *P*-value < 0.001 and q-value > 0.05; (**) *P*-value < 0.001 and q-value < 0.05; (***) *P*-value < 0.0001 and q-value < 0.05. Bold: significant SNP detected by more than one *X. oryzae* strain. The locus ID for each SNP was predicted from the MSU7 rice reference annotation. Intergenic regions were defined as regions without predicted genes or located more than 1 Kb upstream of genes.

| **Pathovar** | **Strain** | **Marker** | **p-value** | **q-value** | **R^2^** | **Effect** | **Effect** | **AILs with Effect** | **Significance** | **Locus** | **Annotation** |
| --- | --- | --- | --- | --- | --- | --- | --- | --- | --- | --- | --- |
|  |  | **(Chr_Pos)** |  |  |  | **estimate** | **allele/Null** | **allele/Null** | **correspondence** |  |  |
| *Xoo* | MAI130 | S1_485081 | 3.5E-04 | 2.2E-01 | 4.9 | 3.0 | C/G | 28/231 | * | LOC_Os01g01890 | expressed protein |
| *Xoo* | MAI130 | S1_498819 | 7.3E-04 | 3.5E-01 | 4.2 | 2.8 | T/C | 29/248 | * | LOC_Os01g01920 | HD domain containing protein 2, putative, expressed |
| *Xoo* | MAI133 | S1_2867144 | 5.9E-04 | 3.4E-01 | 5.0 | -1.7 | C/A | 198/46 | * | LOC_Os01g06000 | PPR repeat containing protein, expressed |
| *Xoo* | MAI133 | S1_2885468 | 4.1E-04 | 3.4E-01 | 5.4 | -1.7 | A/G | 199/50 | * | LOC_Os01g06030 | expressed protein |
| *Xoo* | MAI133 | S1_2885521 | 3.0E-04 | 3.4E-01 | 5.7 | -1.8 | T/C | 198/45 | * | LOC_Os01g06030 | expressed protein |
| *Xoo* | MAI133 | S1_2899652 | 4.4E-04 | 3.4E-01 | 5.4 | -1.7 | G/A | 198/47 | * | LOC_Os01g06070 | expressed protein |
| *Xoc* | MAI123 | S1_5574004 | 3.2E-04 | 5.2E-01 | 5.2 | -2.6 | A/G | 199/36 | * | intergenic |  |
| *Xoc* | MAI123 | S1_5593624 | 7.4E-05 | 5.2E-01 | 7.1 | -2.9 | A/T | 182/36 | * | LOC_Os01g10530 | expressed protein |
| *Xoo* | MAI134 | S1_18200721 | 4.5E-04 | 9.1E-01 | 5.5 | -1.8 | C/G | 236/16 | * | intergenic |  |
| *Xoo* | MAI134 | S1_32124854 | 3.2E-04 | 9.1E-01 | 5.2 | -1.2 | T/C | 234/40 | * | LOC_Os01g55770 | expressed protein |
|  |  |  |  |  |  |  |  |  |  |  |  |
| *Xoo* | MAI133 | S2_2838357 | 2.1E-04 | 3.4E-01 | 5.8 | 1.4 | T/G | 131/112 | * | LOC_Os02g05770 | expressed protein |
| *Xoc* | MAI61 | S2_5164029 | 1.5E-04 | 9.1E-01 | 5.9 | -2.2 | C/T | 141/84 | * | intergenic |  |
| *Xoc* | MAI61 | S2_5254453 | 8.3E-04 | 9.1E-01 | 4.6 | -1.9 | G/A | 166/82 | * | LOC_Os02g10090 | expressed protein |
| *Xoc* | MAI61 | S2_5259884 | 4.6E-04 | 9.1E-01 | 5.1 | -2.0 | G/T | 158/82 | * | LOC_Os02g10100 | leucine-rich repeat receptor protein kinase EXS precursor, putative, expressed |
| *Xoc* | MAI61 | S2_5281537 | 7.3E-04 | 9.1E-01 | 4.6 | -2.0 | C/T | 160/77 | * | LOC_Os02g10120 | lipoxygenase, putative, expressed |
| *Xoc* | MAI10 | S2_10360915 | 2.9E-04 | 3.0E-01 | 5.0 | -2.4 | A/G | 139/119 | * | LOC_Os02g17880 | glycosyl hydrolases family 16, putative, expressed |
| *Xoo* | MAI134 | S2_23098915 | 6.8E-04 | 9.1E-01 | 4.7 | -1.6 | A/C | 252/19 | * | LOC_Os02g38180 | expressed protein |
| *Xoc* | MAI139 | S2_23223948 | 8.5E-04 | 6.8E-01 | 5.7 | -4.2 | G/A | 183/32 | * | intergenic |  |
| *Xoo* | MAI134 | S2_23384851 | 9.9E-04 | 9.1E-01 | 4.3 | -1.5 | A/G | 268/21 | * | LOC_Os02g38690 | protein phosphatase 2C containing protein, expressed |
| *Xoc* | BLS256 | S2_23384882 | 3.7E-04 | 1.1E-01 | 5.5 | -11.7 | T/G | 212/24 | * | LOC_Os02g38690 | protein phosphatase 2C containing protein, expressed |
| *Xoc* | MAI67 | S2_23467758 | 3.7E-04 | 3.7E-01 | 4.9 | -1.7 | T/G | 201/51 | * | LOC_Os02g38820 | expressed protein |
| *Xoc* | BLS256 | S2_23593375 | 7.7E-04 | 1.7E-01 | 4.4 | -11.0 | C/T | 232/24 | * | intergenic |  |
| *Xoc* | BLS256 | S2_23684061 | 7.5E-04 | 1.7E-01 | 4.5 | -11.0 | C/T | 232/24 | * | LOC_Os02g39210 | expressed protein |
| *Xoc* | BLS256 | S2_23819009 | 1.9E-05 | 3.6E-02 | 7.3 | -10.7 | A/T | 204/45 | *** | LOC_Os02g39470 | cyclin, N-terminal domain containing protein, expressed |
| *Xoc* | BLS256 | S2_23826988 | 1.6E-04 | 8.6E-02 | 6.3 | -8.0 | C/A | 150/79 | * | LOC_Os02g39480 | protein phosphatase 2C, putative, expressed |
| *Xoc* | BLS256 | S2_23827020 | 9.0E-04 | 1.8E-01 | 5.1 | -7.1 | A/G | 154/75 | * | LOC_Os02g39480 | protein phosphatase 2C, putative, expressed |

**Continuation Table S5.**

| **Pathovar** | **Strain** | **Marker** | **p-value** | **q-value** | **R^2^** | **Effect** | **Effect** | **AILs with Effect** | **Significance** | **Locus** | **Annotation** |
| --- | --- | --- | --- | --- | --- | --- | --- | --- | --- | --- | --- |
|  |  | **(Chr_Pos)** |  |  |  | **estimate** | **allele/Null** | **allele/Null** | **correspondence** |  |  |
| *Xoc* | BLS256 | S2_23874924 | 5.6E-05 | 5.7E-02 | 6.5 | -11.7 | A/G | 218/31 | * | LOC_Os02g39550 | calcium-binding mitochondrial protein anon-60Da, putative, expressed |
| *Xoc* | BLS256 | S2_24006214 | 3.0E-05 | 4.2E-02 | 7.9 | -12.1 | C/T | 188/33 | *** | LOC_Os02g39750 | inorganic phosphate transporter, putative, expressed |
| *Xoc* | BLS256 | S2_24108025 | 7.6E-04 | 1.7E-01 | 4.7 | -7.3 | A/C | 166/71 | * | intergenic |  |
| *Xoc* | BLS256 | S2_24122049 | 9.6E-04 | 1.9E-01 | 4.7 | -6.7 | T/G | 141/92 | * | intergenic |  |
| *Xoc* | BLS256 | S2_24135986 | 1.8E-04 | 8.6E-02 | 5.6 | -12.5 | C/T | 227/24 | * | LOC_Os02g39910 | B4-BTB1 - Bric-a-Brac, Tramtrack, Broad Complex BTB domain with B4 subfamily conserved sequence, expressed |
| *Xoc* | BLS256 | S2_24234623 | 7.0E-04 | 1.7E-01 | 4.5 | -10.9 | A/G | 229/26 | * | intergenic |  |
| *Xoc* | BLS256 | S2_24273471 | 3.6E-04 | 1.1E-01 | 5.1 | -11.5 | A/G | 224/25 | * | LOC_Os02g40090 | transporter-related, putative, expressed |
| *Xoc* | MAI46 | S2_24278919 | 2.6E-04 | 2.9E-01 | 5.2 | -5.9 | G/C | 255/21 | * | LOC_Os02g40100 | plant protein of unknown function DUF869 domain containing protein, expressed |
| *Xoc* | BLS256 | S2_24405470 | 7.3E-04 | 1.7E-01 | 4.7 | -10.3 | G/A | 223/28 | * | LOC_Os02g40320 | PINHEAD, putative, expressed |
| *Xoc* | BLS256 | S2_24479319 | 3.3E-04 | 1.1E-01 | 5.1 | -11.4 | C/G | 226/26 | * | intergenic |  |
| *Xoc* | BLS256 | S2_24480075 | 4.0E-04 | 1.2E-01 | 4.9 | -11.6 | T/C | 229/25 | * | intergenic |  |
| *Xoc* | BLS256 | S2_24504679 | 6.1E-04 | 1.6E-01 | 4.7 | -8.3 | A/G | 196/51 | * | intergenic |  |
| *Xoc* | MAI10 | **S2_24509874** | 4.6E-04 | 3.9E-01 | 4.5 | -3.9 | A/G | 260/19 | * | intergenic |  |
| *Xoc* | MAI46 | **S2_24509874** | 7.9E-05 | 2.9E-01 | 6.5 | -6.7 | A/G | 258/19 | * | intergenic |  |
| *Xoc* | MAI10 | **S2_24527947** | 3.2E-04 | 3.1E-01 | 4.9 | -4.0 | G/A | 257/19 | * | LOC_Os02g40454 | serine/threonine protein phosphatase 2A 55 kDa regulatory subunit B, putative, expressed |
| *Xoc* | MAI46 | **S2_24527947** | 1.1E-04 | 2.9E-01 | 5.8 | -6.5 | G/A | 256/19 | * | LOC_Os02g40454 | serine/threonine protein phosphatase 2A 55 kDa regulatory subunit B, putative, expressed |
| *Xoc* | BLS256 | S2_24575274 | 3.1E-04 | 1.0E-01 | 5.1 | -5.8 | A/G | 239/20 | * | LOC_Os02g40514 | h/ACA ribonucleoprotein complex subunit 3, putative, expressed |
| *Xoc* | BLS256 | S2_24679759 | 3.7E-05 | 4.2E-02 | 6.8 | -11.3 | C/T | 215/36 | *** | intergenic |  |
| *Xoc* | BLS256 | S2_24688545 | 9.4E-05 | 6.8E-02 | 7.0 | -10.5 | C/G | 190/38 | * | LOC_Os02g40720 | expressed protein |
| *Xoc* | BLS256 | **S2_24691050** | 3.0E-04 | 1.0E-01 | 5.7 | -8.0 | A/G | 165/61 | * | LOC_Os02g40730 | ammonium transporter protein, putative, expressed |
| *Xoc* | MAI77 | **S2_24691050** | 1.0E-03 | 6.4E-01 | 4.6 | -2.0 | A/G | 166/69 | * | LOC_Os02g40730 | ammonium transporter protein, putative, expressed |
| *Xoc* | BLS256 | **S2_24692377** | 3.9E-04 | 1.2E-01 | 5.1 | -9.4 | G/T | 210/37 | * | LOC_Os02g40730 | ammonium transporter protein, putative, expressed |
| *Xoc* | MAI67 | **S2_24692377** | 4.4E-04 | 4.1E-01 | 4.9 | -1.8 | G/T | 216/41 | * | LOC_Os02g40730 | ammonium transporter protein, putative, expressed |
| *Xoc* | BLS256 | S2_24754613 | 1.5E-04 | 8.6E-02 | 5.7 | -10.1 | G/A | 215/38 | * | LOC_Os02g40840 | alcohol oxidase, putative, expressed |
| *Xoc* | BLS256 | S2_24786166 | 1.1E-04 | 7.6E-02 | 5.8 | -10.4 | C/T | 218/37 | * | LOC_Os02g40900 | RNA recognition motif containing protein, putative, expressed |
| *Xoc* | BLS256 | S2_24786718 | 8.3E-05 | 6.7E-02 | 6.1 | -10.8 | A/C | 216/36 | * | LOC_Os02g40900 | RNA recognition motif containing protein, putative, expressed |
| *Xoc* | BLS256 | S2_24791557 | 8.3E-04 | 1.7E-01 | 4.6 | -8.9 | C/G | 208/38 | * | intergenic |  |
| *Xoc* | BLS256 | **S2_24848131** | 1.8E-04 | 8.6E-02 | 5.5 | -9.2 | A/T | 203/49 | * | intergenic |  |
| *Xoc* | MAI67 | **S2_24848131** | 9.9E-04 | 5.7E-01 | 4.7 | -1.5 | A/T | 211/50 | * | intergenic |  |

**Continuation Table S5.**

| **Pathovar** | **Strain** | **Marker** | **p-value** | **q-value** | **R^2^** | **Effect** | **Effect** | **AILs with Effect** | **Significance** | **Locus** | **Annotation** |
| --- | --- | --- | --- | --- | --- | --- | --- | --- | --- | --- | --- |
|  |  | **(Chr_Pos)** |  |  |  | **estimate** | **allele/Null** | **allele/Null** | **correspondence** |  |  |
| *Xoc* | BLS256 | S2_24874086 | 1.6E-04 | 8.6E-02 | 5.6 | -9.5 | G/A | 202/47 | * | LOC_Os02g41500 | OsWAK13 - OsWAK receptor-like protein kinase, expressed |
| *Xoc* | BLS256 | **S2_24877663** | 2.2E-04 | 8.8E-02 | 5.4 | -9.1 | T/A | 203/49 | * | intergenic |  |
| *Xoc* | MAI67 | **S2_24877663** | 9.5E-04 | 5.7E-01 | 4.7 | -1.5 | T/A | 211/50 | * | intergenic |  |
| *Xoc* | BLS256 | **S2_24937103** | 5.3E-04 | 1.4E-01 | 5.2 | -8.4 | G/A | 193/51 | * | intergenic |  |
| *Xoc* | MAI67 | **S2_24937103** | 5.5E-04 | 4.8E-01 | 4.7 | -1.6 | G/A | 199/56 | * | intergenic |  |
| *Xoc* | BLS256 | S2_25075755 | 3.7E-05 | 4.2E-02 | 7.0 | -11.4 | G/A | 200/37 | *** | LOC_Os02g41720 | transposon protein, putative, unclassified, expressed |
| *Xoc* | BLS256 | S2_25400243 | 8.7E-04 | 1.7E-01 | 4.4 | -10.8 | C/T | 230/23 | * | LOC_Os02g42220 | transposon protein, putative, unclassified, expressed |
| *Xoc* | BLS256 | S2_25596670 | 5.7E-04 | 1.5E-01 | 4.7 | 8.0 | T/C | 53/199 | * | LOC_Os02g42585 | AP2 domain containing protein, expressed |
| *Xoc* | BLS256 | **S2_25618890** | 2.6E-04 | 9.2E-02 | 5.8 | 6.7 | G/A | 107/137 | * | LOC_Os02g42590 | WD-40 repeat family protein, putative, expressed |
| *Xoc* | MAI139 | **S2_25618890** | 5.2E-04 | 5.3E-01 | 5.3 | 2.9 | G/A | 104/123 | * | LOC_Os02g42590 | WD-40 repeat family protein, putative, expressed |
| *Xoc* | BLS256 | **S2_25639335** | 6.3E-06 | 3.0E-02 | 8.5 | 8.6 | T/A | 139/108 | *** | LOC_Os02g42620 | protein kinase, putative, expressed |
| *Xoc* | MAI139 | **S2_25639335** | 3.0E-04 | 3.3E-01 | 5.9 | 3.1 | T/A | 134/97 | * | LOC_Os02g42620 | protein kinase, putative, expressed |
| *Xoc* | BLS256 | **S2_25639338** | 6.3E-06 | 3.0E-02 | 8.5 | 8.6 | A/G | 139/108 | *** | LOC_Os02g42620 | protein kinase, putative, expressed |
| *Xoc* | MAI139 | **S2_25639338** | 3.0E-04 | 3.3E-01 | 5.9 | 3.1 | A/G | 134/97 | * | LOC_Os02g42620 | protein kinase, putative, expressed |
| *Xoc* | BLS256 | **S2_25639568** | 1.2E-05 | 3.6E-02 | 7.9 | 8.4 | C/T | 140/107 | *** | LOC_Os02g42620 | protein kinase, putative, expressed |
| *Xoc* | MAI139 | **S2_25639568** | 1.7E-04 | 2.2E-01 | 6.5 | 3.2 | C/T | 135/96 | * | LOC_Os02g42620 | protein kinase, putative, expressed |
| *Xoc* | BLS256 | S2_25654974 | 1.1E-04 | 7.3E-02 | 6.1 | 7.2 | C/A | 115/132 | * | LOC_Os02g42650 | expansin precursor, putative, expressed |
| *Xoc* | BLS256 | S2_25672672 | 6.6E-04 | 1.6E-01 | 5.0 | 7.0 | T/C | 82/143 | * | LOC_Os02g42690 | zinc finger, C3HC4 type domain containing protein, expressed |
| *Xoc* | BLS256 | **S2_25803692** | 2.8E-05 | 4.2E-02 | 7.2 | 7.8 | A/C | 102/140 | *** | LOC_Os02g42900 | expressed protein |
| *Xoc* | MAI46 | **S2_25803692** | 7.1E-04 | 5.4E-01 | 4.5 | 3.0 | A/C | 114/145 | * | LOC_Os02g42900 | expressed protein |
| *Xoc* | MAI61 | **S2_25803692** | 8.6E-04 | 9.1E-01 | 4.5 | 1.8 | A/C | 107/137 | * | LOC_Os02g42900 | expressed protein |
| *Xoc* | BLS256 | S2_25885474 | 6.5E-04 | 1.6E-01 | 4.7 | 6.3 | C/G | 119/124 | * | LOC_Os02g43000 | cysteine-rich repeat secretory protein 15 precursor, putative, expressed |
| *Xoc* | BAI5 | S2_26070743 | 2.8E-04 | 7.5E-02 | 4.6 | -6.1 | A/T | 216/79 | * | intergenic |  |
| *Xoc* | BAI5 | **S2_26126554** | 4.8E-04 | 1.2E-01 | 4.7 | -6.1 | C/A | 201/68 | * | LOC_Os02g43314 | expressed protein |
| *Xoc* | MAI61 | **S2_26126554** | 9.5E-05 | 9.1E-01 | 6.3 | -2.6 | C/A | 167/60 | * | LOC_Os02g43314 | expressed protein |
| *Xoc* | MAI77 | **S2_26126554** | 3.8E-04 | 3.9E-01 | 5.2 | -2.2 | C/A | 169/62 | * | LOC_Os02g43314 | expressed protein |
| *Xoc* | MAI123 | **S2_26126554** | 5.2E-04 | 5.8E-01 | 5.1 | -2.1 | C/A | 163/58 | * | LOC_Os02g43314 | expressed protein |
| *Xoc* | BAI5 | S2_26329353 | 7.3E-04 | 1.7E-01 | 3.9 | 5.2 | G/T | 117/174 | * | LOC_Os02g43630 | hypothetical protein |
| *Xoc* | BAI5 | S2_26349262 | 8.7E-04 | 1.9E-01 | 3.8 | -5.6 | G/A | 220/76 | * | LOC_Os02g43670 | transferase family protein, putative, expressed |

**Continuation Table S5.**

| **Pathovar** | **Strain** | **Marker** | **p-value** | **q-value** | **R^2^** | **Effect** | **Effect** | **AILs with Effect** | **Significance** | **Locus** | **Annotation** |
| --- | --- | --- | --- | --- | --- | --- | --- | --- | --- | --- | --- |
|  |  | **(Chr_Pos)** |  |  |  | **estimate** | **allele/Null** | **allele/Null** | **correspondence** |  |  |
| *Xoc* | MAI77 | **S2_27203917** | 6.3E-04 | 5.2E-01 | 4.8 | -2.6 | A/G | 204/35 | * | LOC_Os02g44900 | expressed protein |
| *Xoo* | BAI3 | **S2_27203917** | 5.9E-04 | 8.8E-02 | 4.2 | -4.2 | A/G | 237/40 | * | LOC_Os02g44900 | expressed protein |
| *Xoc* | MAI77 | S2_27204005 | 9.8E-04 | 6.4E-01 | 4.1 | -2.6 | G/A | 236/32 | * | LOC_Os02g44900 | expressed protein |
| *Xoo* | MAI136 | S2_29845695 | 8.6E-04 | 3.2E-01 | 5.2 | -3.3 | C/G | 184/37 | * | intergenic |  |
| *Xoo* | MAI145 | S2_34796411 | 7.3E-04 | 2.5E-01 | 4.4 | -2.2 | G/T | 160/101 | * | LOC_Os02g56750 | OsFBX65 - F-box domain containing protein, expressed |
| *Xoo* | MAI130 | S2_35216593 | 1.2E-04 | 1.1E-01 | 5.6 | -2.3 | G/C | 225/53 | * | LOC_Os02g57460 | RING-H2 finger protein ATL5G, putative, expressed |
| *Xoo* | MAI130 | S2_35217191 | 2.9E-04 | 2.0E-01 | 4.9 | -2.2 | T/C | 224/55 | * | LOC_Os02g57460 | RING-H2 finger protein ATL5G, putative, expressed |
|  |  |  |  |  |  |  |  |  |  |  |  |
| *Xoc* | BLS256 | S3_9153681 | 8.5E-04 | 1.7E-01 | 4.6 | 9.1 | A/G | 214/29 | * | intergenic |  |
| *Xoc* | MAI10 | S3_12629690 | 8.4E-04 | 5.8E-01 | 5.4 | 2.2 | C/T | 74/157 | * | LOC_Os03g22040 | expressed protein |
| *Xoc* | MAI10 | S3_12647861 | 8.8E-05 | 1.2E-01 | 5.8 | 2.4 | T/G | 82/189 | * | LOC_Os03g22070 | expressed protein |
| *Xoc* | MAI10 | S3_12647903 | 4.9E-04 | 3.9E-01 | 4.6 | 2.2 | C/G | 78/177 | * | LOC_Os03g22070 | expressed protein |
| *Xoc* | MAI10 | S3_12647983 | 1.5E-04 | 1.8E-01 | 5.3 | 2.3 | T/C | 85/187 | * | LOC_Os03g22070 | expressed protein |
| *Xoo* | CFBP1951 | S3_14528215 | 8.7E-04 | 4.4E-01 | 4.7 | -1.4 | T/G | 214/20 | * | LOC_Os03g25400 | kinase, putative, expressed |
| *Xoo* | MAI133 | S3_34779286 | 8.1E-04 | 3.4E-01 | 4.6 | -2.1 | A/G | 216/26 | * | LOC_Os03g61240 | expressed protein |
| *Xoo* | MAI133 | S3_34793557 | 6.7E-04 | 3.4E-01 | 5.8 | -2.1 | A/G | 206/27 | * | LOC_Os03g61270 | OsMan04 - Endo-Beta-Mannanase, expressed |
| *Xoo* | MAI133 | S3_34793568 | 6.7E-04 | 3.4E-01 | 5.8 | -2.1 | T/C | 206/27 | * | LOC_Os03g61270 | OsMan04 - Endo-Beta-Mannanase, expressed |
| *Xoo* | MAI133 | S3_34826622 | 4.5E-04 | 3.4E-01 | 5.3 | -2.1 | T/G | 204/29 | * | LOC_Os03g61360 | hydrolase, alpha/beta fold family domain containing protein, expressed |
| *Xoo* | MAI133 | S3_34826623 | 4.5E-04 | 3.4E-01 | 5.3 | -2.1 | A/C | 204/29 | * | LOC_Os03g61360 | hydrolase, alpha/beta fold family domain containing protein, expressed |
| *Xoo* | MAI134 | S3_34870865 | 4.4E-04 | 9.1E-01 | 5.4 | -1.6 | G/A | 255/21 | * | LOC_Os03g61470 | uncharacterized Cys-rich domain containing protein, putative, expressed |
|  |  |  |  |  |  |  |  |  |  |  |  |
| *Xoc* | MAI10 | S4_1000522 | 2.9E-04 | 3.0E-01 | 5.2 | -2.3 | C/T | 98/157 | * | LOC_Os04g02650 | expressed protein |
| *Xoc* | MAI10 | S4_3531897 | 3.6E-04 | 3.3E-01 | 5.6 | -2.3 | G/C | 182/79 | * | LOC_Os04g06700 | expressed protein |
| *Xoo* | MAI101 | S4_24924317 | 3.9E-04 | 1.4E-01 | 6.1 | -5.1 | A/G | 198/22 | * | LOC_Os04g42100 | retrotransposon protein, putative, unclassified, expressed |
| *Xoo* | MAI101 | S4_24924363 | 3.9E-04 | 1.4E-01 | 6.1 | -5.1 | G/A | 198/22 | * | LOC_Os04g42100 | retrotransposon protein, putative, unclassified, expressed |
| *Xoc* | BAI5 | S4_29482889 | 3.9E-05 | 1.2E-02 | 5.8 | 6.1 | G/C | 208/86 | *** | intergenic |  |
| *Xoc* | BAI5 | **S4_29548991** | 1.7E-09 | 2.1E-06 | 12.4 | 13.1 | G/A | 273/35 | *** | intergenic |  |
| *Xoo* | BAI3 | **S4_29548991** | 5.8E-04 | 8.8E-02 | 4.0 | 4.6 | G/A | 273/37 | * | intergenic |  |
| *Xoc* | BAI5 | S4_29602194 | 8.0E-04 | 1.8E-01 | 3.7 | 9.6 | G/A | 289/20 | * | intergenic |  |

**Continuation Table S5.**

| **Pathovar** | **Strain** | **Marker** | **p-value** | **q-value** | **R^2^** | **Effect** | **Effect** | **AILs with Effect** | **Significance** | **Locus** | **Annotation** |
| --- | --- | --- | --- | --- | --- | --- | --- | --- | --- | --- | --- |
|  |  | **(Chr_Pos)** |  |  |  | **estimate** | **allele/Null** | **allele/Null** | **correspondence** |  |  |
| *Xoc* | BAI5 | **S4_29783427** | 1.1E-07 | 7.4E-05 | 9.8 | -9.2 | G/A | 69/232 | *** | LOC_Os04g49930 | sensitivity to red light reduced protein 1, putative, expressed |
| *Xoo* | BAI3 | **S4_29783427** | 5.9E-04 | 8.8E-02 | 3.9 | -3.8 | G/A | 72/232 | * | LOC_Os04g49930 | sensitivity to red light reduced protein 1, putative, expressed |
| *Xoc* | BAI5 | S4_29788028 | 3.7E-06 | 1.4E-03 | 8.1 | -8.1 | T/C | 63/206 | *** | LOC_Os04g49940 | expressed protein |
| *Xoc* | BAI5 | **S4_29797214** | 5.7E-07 | 3.0E-04 | 8.8 | -8.7 | G/A | 69/230 | *** | LOC_Os04g49950 | OsFBX150 - F-box domain containing protein, expressed |
| *Xoo* | BAI3 | **S4_29797214** | 6.5E-04 | 9.2E-02 | 4.0 | -3.8 | G/A | 72/230 | * | LOC_Os04g49950 | OsFBX150 - F-box domain containing protein, expressed |
| *Xoc* | BAI5 | **S4_29803185** | 1.6E-07 | 9.4E-05 | 9.6 | -9.2 | A/G | 69/232 | *** | LOC_Os04g49960 | glycosyl transferase, group 1 domain containing protein, expressed |
| *Xoo* | BAI3 | **S4_29803185** | 6.3E-04 | 9.0E-02 | 3.9 | -3.8 | A/G | 72/232 | * | LOC_Os04g49960 | glycosyl transferase, group 1 domain containing protein, expressed |
| *Xoc* | BAI5 | **S4_29809870** | 1.4E-07 | 8.7E-05 | 9.9 | -9.1 | T/A | 70/231 | *** | LOC_Os04g49970 | U-box, putative, expressed |
| *Xoo* | BAI3 | **S4_29809870** | 4.7E-04 | 7.6E-02 | 4.1 | -3.8 | T/A | 73/231 | * | LOC_Os04g49970 | U-box, putative, expressed |
| *Xoc* | BAI5 | S4_29817523 | 1.5E-06 | 6.7E-04 | 9.1 | -8.5 | T/G | 61/210 | *** | LOC_Os04g49990 | AT hook motif domain containing protein, expressed |
| *Xoc* | BAI5 | S4_29817535 | 5.8E-06 | 2.0E-03 | 7.9 | -7.9 | C/T | 63/210 | *** | LOC_Os04g49990 | AT hook motif domain containing protein, expressed |
| *Xoc* | BAI5 | S4_29817550 | 5.8E-06 | 2.0E-03 | 7.9 | -7.9 | G/A | 63/210 | *** | LOC_Os04g49990 | AT hook motif domain containing protein, expressed |
| *Xoc* | BAI5 | S4_29832522 | 1.0E-04 | 3.0E-02 | 5.5 | -6.6 | A/G | 76/200 | ** | LOC_Os04g50030 | DNA binding protein, putative, expressed |
| *Xoc* | BAI5 | S4_29866691 | 3.6E-05 | 1.1E-02 | 6.0 | -6.8 | T/A | 81/219 | *** | LOC_Os04g50080 | expressed protein |
| *Xoc* | BAI5 | **S4_29868104** | 1.1E-05 | 3.6E-03 | 6.8 | -7.4 | C/T | 78/219 | *** | intergenic |  |
| *Xoo* | MAI130 | **S4_29868104** | 6.3E-04 | 3.3E-01 | 4.5 | -2.1 | C/T | 73/198 | * | intergenic |  |
| *Xoc* | BAI5 | **S4_29922917** | 1.1E-04 | 3.2E-02 | 5.6 | -6.5 | T/C | 73/196 | ** | intergenic |  |
| *Xoo* | BAI3 | **S4_29922917** | 2.3E-04 | 4.3E-02 | 4.9 | -3.9 | T/C | 76/196 | ** | intergenic |  |
| *Xoc* | BLS256 | S4_29965442 | 7.2E-04 | 1.7E-01 | 4.7 | 6.6 | C/T | 95/147 | * | LOC_Os04g50204 | protein transport protein-related, putative, expressed |
| *Xoc* | BAI5 | **S4_29966056** | 2.5E-06 | 1.0E-03 | 7.6 | -8.1 | C/G | 67/234 | *** | LOC_Os04g50204 | protein transport protein-related, putative, expressed |
| *Xoo* | BAI3 | **S4_29966056** | 1.8E-04 | 3.7E-02 | 4.7 | -4.1 | C/G | 70/233 | ** | LOC_Os04g50204 | protein transport protein-related, putative, expressed |
| *Xoc* | BAI5 | **S4_29968457** | 1.0E-05 | 3.4E-03 | 7.0 | -7.5 | T/C | 68/227 | *** | intergenic |  |
| *Xoo* | BAI3 | **S4_29968457** | 9.1E-04 | 1.2E-01 | 3.7 | -3.6 | T/C | 71/227 | * | intergenic |  |
| *Xoc* | BAI5 | **S4_29968490** | 1.1E-05 | 3.6E-03 | 6.9 | -7.5 | G/C | 67/231 | *** | intergenic |  |
| *Xoo* | BAI3 | **S4_29968490** | 1.7E-04 | 3.5E-02 | 4.9 | -4.1 | G/C | 70/231 | ** | intergenic |  |
| *Xoo* | MAI130 | S4_30019173 | 6.6E-04 | 3.3E-01 | 4.7 | -1.9 | G/A | 101/170 | * | LOC_Os04g50740 | ara54-like RING finger protein, putative, expressed |
| *Xoo* | MAI130 | S4_30047908 | 2.3E-05 | 2.6E-02 | 7.1 | -2.4 | T/A | 84/185 | *** | LOC_Os04g50790 | RNA recognition motif, putative, expressed |

**Continuation Table S5.**

| **Pathovar** | **Strain** | **Marker** | **p-value** | **q-value** | **R^2^** | **Effect** | **Effect** | **AILs with Effect** | **Significance** | **Locus** | **Annotation** |
| --- | --- | --- | --- | --- | --- | --- | --- | --- | --- | --- | --- |
|  |  | **(Chr_Pos)** |  |  |  | **estimate** | **allele/Null** | **allele/Null** | **correspondence** |  |  |
| *Xoo* | MAI130 | S4_30080184 | 4.2E-05 | 4.1E-02 | 6.5 | -2.3 | G/A | 82/178 | *** | LOC_Os04g50860 | expressed protein |
| *Xoc* | BAI5 | S4_30764890 | 2.6E-06 | 1.0E-03 | 8.7 | -7.7 | A/G | 77/194 | *** | LOC_Os04g51880 | GHMP kinases ATP-binding protein, putative, expressed |
| *Xoc* | BAI5 | S4_30790417 | 3.7E-08 | 3.4E-05 | 10.8 | -8.9 | G/T | 79/213 | *** | intergenic |  |
| *Xoc* | BAI5 | S4_30808530 | 4.1E-07 | 2.3E-04 | 9.2 | -8.6 | A/G | 75/219 | *** | LOC_Os04g51920 | protein disulfide isomerase, putative, expressed |
| *Xoc* | BAI5 | S4_31263800 | 1.0E-07 | 6.9E-05 | 9.5 | 15.7 | C/T | 295/16 | *** | LOC_Os04g52590 | protein kinase domain containing protein, expressed |
| *Xoc* | BAI5 | S4_31263806 | 1.0E-07 | 6.9E-05 | 9.5 | 15.7 | T/C | 295/16 | *** | LOC_Os04g52590 | protein kinase domain containing protein, expressed |
| *Xoc* | BAI5 | S4_31263820 | 1.0E-07 | 6.9E-05 | 9.5 | 15.7 | C/T | 295/16 | *** | LOC_Os04g52590 | protein kinase domain containing protein, expressed |
| *Xoc* | BAI5 | S4_31263829 | 9.4E-07 | 4.6E-04 | 8.3 | 13.9 | C/T | 278/17 | *** | LOC_Os04g52590 | protein kinase domain containing protein, expressed |
| *Xoc* | BAI5 | S4_31264880 | 1.7E-06 | 7.1E-04 | 8.1 | 13.9 | A/G | 278/17 | *** | LOC_Os04g52590 | protein kinase domain containing protein, expressed |
| *Xoc* | BAI5 | S4_31264887 | 1.7E-06 | 7.1E-04 | 8.1 | 13.9 | T/C | 278/17 | *** | LOC_Os04g52590 | protein kinase domain containing protein, expressed |
| *Xoc* | BAI5 | S4_31264905 | 8.1E-07 | 4.0E-04 | 8.1 | 14.4 | G/A | 294/17 | *** | LOC_Os04g52590 | protein kinase domain containing protein, expressed |
| *Xoc* | BAI5 | S4_31316473 | 6.6E-07 | 3.4E-04 | 9.6 | 14.0 | C/T | 261/18 | *** | LOC_Os04g52630 | leucine-rich repeat-containing protein kinase family protein, putative, expressed |
| *Xoc* | BAI5 | **S4_31420093** | 1.1E-04 | 3.2E-02 | 5.0 | 9.7 | A/T | 286/21 | ** | LOC_Os04g52770 | helix-loop-helix DNA-binding domain containing protein, expressed |
| *Xoo* | BAI3 | **S4_31420093** | 2.7E-05 | 8.5E-03 | 5.8 | 6.9 | A/T | 289/20 | *** | LOC_Os04g52770 | helix-loop-helix DNA-binding domain containing protein, expressed |
| *Xoc* | BAI5 | **S4_31498999** | 8.0E-08 | 6.4E-05 | 9.8 | 15.1 | G/C | 290/17 | *** | LOC_Os04g52890 | ATBAG1, putative, expressed |
| *Xoc* | MAI139 | **S4_31498999** | 6.2E-04 | 5.6E-01 | 4.9 | 6.2 | G/C | 226/12 | * | LOC_Os04g52890 | ATBAG1, putative, expressed |
| *Xoc* | BAI5 | **S4_31531412** | 8.7E-04 | 1.9E-01 | 3.7 | -4.8 | T/A | 92/209 | * | LOC_Os04g52940 | SIT4 phosphatase-associated protein domain containing protein, expressed |
| *Xoc* | MAI67 | **S4_31531412** | 6.1E-04 | 4.9E-01 | 4.5 | -1.3 | T/A | 84/177 | * | LOC_Os04g52940 | SIT4 phosphatase-associated protein domain containing protein, expressed |
| *Xoo* | BAI3 | **S4_31531412** | 1.8E-05 | 6.3E-03 | 6.1 | -4.0 | T/A | 93/211 | *** | LOC_Os04g52940 | SIT4 phosphatase-associated protein domain containing protein, expressed |
| *Xoo* | MAI70 | **S4_31531412** | 1.9E-04 | 1.7E-01 | 5.1 | -3.3 | T/A | 88/198 | * | LOC_Os04g52940 | SIT4 phosphatase-associated protein domain containing protein, expressed |
| *Xoo* | MAI130 | **S4_31531412** | 4.0E-05 | 4.1E-02 | 6.8 | -2.2 | T/A | 85/190 | *** | LOC_Os04g52940 | SIT4 phosphatase-associated protein domain containing protein, expressed |
| *Xoc* | MAI46 | **S4_31553238** | 1.5E-04 | 2.9E-01 | 6.8 | -4.4 | T/C | 194/45 | * | LOC_Os04g52970 | NBS-LRR disease resistance protein, putative, expressed |
| *Xoc* | MAI123 | **S4_31553238** | 4.3E-04 | 5.2E-01 | 6.2 | -2.4 | T/C | 176/42 | * | LOC_Os04g52970 | NBS-LRR disease resistance protein, putative, expressed |
| *Xoc* | MAI46 | **S4_31553264** | 1.5E-04 | 2.9E-01 | 6.8 | -4.4 | T/C | 194/45 | * | LOC_Os04g52970 | NBS-LRR disease resistance protein, putative, expressed |
| *Xoc* | MAI123 | **S4_31553264** | 4.3E-04 | 5.2E-01 | 6.2 | -2.4 | T/C | 176/42 | * | LOC_Os04g52970 | NBS-LRR disease resistance protein, putative, expressed |
| *Xoc* | MAI46 | **S4_31559540** | 2.5E-04 | 2.9E-01 | 5.9 | -3.7 | A/C | 190/67 | * | LOC_Os04g52980 | expressed protein |
| *Xoc* | MAI67 | **S4_31559540** | 8.7E-04 | 5.7E-01 | 4.9 | -1.3 | A/C | 188/65 | * | LOC_Os04g52980 | expressed protein |

**Continuation Table S5.**

| **Pathovar** | **Strain** | **Marker** | **p-value** | **q-value** | **R^2^** | **Effect** | **Effect** | **AILs with Effect** | **Significance** | **Locus** | **Annotation** |
| --- | --- | --- | --- | --- | --- | --- | --- | --- | --- | --- | --- |
|  |  | **(Chr_Pos)** |  |  |  | **estimate** | **allele/Null** | **allele/Null** | **correspondence** |  |  |
| *Xoc* | MAI46 | **S4_31559557** | 2.3E-04 | 2.9E-01 | 5.9 | -3.7 | G/T | 190/66 | * | LOC_Os04g52980 | expressed protein |
| *Xoc* | MAI67 | **S4_31559557** | 8.3E-04 | 5.7E-01 | 5.0 | -1.4 | G/T | 188/64 | * | LOC_Os04g52980 | expressed protein |
| *Xoc* | MAI46 | **S4_31559582** | 2.5E-04 | 2.9E-01 | 5.9 | -3.7 | T/C | 190/67 | * | LOC_Os04g52980 | expressed protein |
| *Xoc* | MAI67 | **S4_31559582** | 8.7E-04 | 5.7E-01 | 4.9 | -1.3 | T/C | 188/65 | * | LOC_Os04g52980 | expressed protein |
| *Xoc* | BAI5 | **S4_31669472** | 5.7E-08 | 4.9E-05 | 10.2 | -7.7 | A/G | 140/160 | *** | LOC_Os04g53195 | expressed protein |
| *Xoo* | MAI130 | **S4_31669472** | 9.2E-06 | 1.2E-02 | 7.8 | -2.3 | A/G | 130/146 | *** | LOC_Os04g53195 | expressed protein |
| *Xoc* | BAI5 | **S4_31670015** | 1.3E-06 | 6.2E-04 | 8.3 | -7.1 | T/G | 149/146 | *** | LOC_Os04g53190 | CPuORF12 - conserved peptide uORF-containing transcript, expressed |
| *Xoo* | MAI130 | **S4_31670015** | 1.3E-05 | 1.5E-02 | 7.7 | -2.3 | T/G | 136/135 | *** | LOC_Os04g53190 | CPuORF12 - conserved peptide uORF-containing transcript, expressed |
| *Xoc* | BAI5 | **S4_31716597** | 2.9E-17 | 2.1E-13 | 27.7 | 18.2 | G/A | 267/32 | *** | LOC_Os04g53240 | autophagy-related protein, putative, expressed |
| *Xoc* | MAI10 | **S4_31716597** | 3.8E-06 | 2.7E-02 | 8.1 | 4.3 | G/A | 239/26 | *** | LOC_Os04g53240 | autophagy-related protein, putative, expressed |
| *Xoc* | MAI61 | **S4_31716597** | 6.0E-04 | 9.1E-01 | 5.4 | 3.0 | G/A | 223/26 | * | LOC_Os04g53240 | autophagy-related protein, putative, expressed |
| *Xoc* | MAI67 | **S4_31716597** | 3.6E-06 | 5.7E-03 | 8.4 | 2.6 | G/A | 229/29 | *** | LOC_Os04g53240 | autophagy-related protein, putative, expressed |
| *Xoc* | MAI77 | **S4_31716597** | 1.4E-05 | 6.6E-02 | 7.3 | 3.7 | G/A | 232/27 | * | LOC_Os04g53240 | autophagy-related protein, putative, expressed |
| *Xoc* | MAI123 | **S4_31716597** | 2.8E-04 | 5.2E-01 | 5.2 | 3.0 | G/A | 219/25 | * | LOC_Os04g53240 | autophagy-related protein, putative, expressed |
| *Xoc* | MAI139 | **S4_31716597** | 8.3E-05 | 1.7E-01 | 6.8 | 5.1 | G/A | 206/25 | * | LOC_Os04g53240 | autophagy-related protein, putative, expressed |
| *Xoo* | BAI3 | **S4_31716597** | 6.8E-17 | 4.9E-13 | 25.1 | 11.6 | G/A | 270/31 | *** | LOC_Os04g53240 | autophagy-related protein, putative, expressed |
| *Xoo* | CFBP1951 | **S4_31716597** | 8.2E-05 | 7.0E-02 | 6.0 | 1.4 | G/A | 229/29 | * | LOC_Os04g53240 | autophagy-related protein, putative, expressed |
| *Xoo* | MAI70 | **S4_31716597** | 3.4E-09 | 2.1E-05 | 12.9 | 7.9 | G/A | 251/30 | *** | LOC_Os04g53240 | autophagy-related protein, putative, expressed |
| *Xoo* | MAI101 | **S4_31716597** | 2.6E-05 | 2.2E-02 | 7.3 | 5.3 | G/A | 221/26 | *** | LOC_Os04g53240 | autophagy-related protein, putative, expressed |
| *Xoo* | MAI130 | **S4_31716597** | 2.3E-06 | 5.3E-03 | 8.5 | 3.7 | G/A | 242/29 | *** | LOC_Os04g53240 | autophagy-related protein, putative, expressed |
| *Xoo* | MAI136 | **S4_31716597** | 2.2E-05 | 4.7E-02 | 7.1 | 4.3 | G/A | 225/29 | *** | LOC_Os04g53240 | autophagy-related protein, putative, expressed |
| *Xoo* | MAI145 | **S4_31716597** | 1.0E-04 | 6.0E-02 | 5.7 | 3.9 | G/A | 234/29 | * | LOC_Os04g53240 | autophagy-related protein, putative, expressed |
| *Xoc* | BAI5 | S4_31721763 | 6.3E-04 | 1.5E-01 | 3.9 | -5.9 | C/A | 237/63 | * | LOC_Os04g53250 | polyphenol oxidase protein, putative, expressed |

**Continuation Table S5.**

| **Pathovar** | **Strain** | **Marker** | **p-value** | **q-value** | **R^2^** | **Effect** | **Effect** | **AILs with Effect** | **Significance** | **Locus** | **Annotation** |
| --- | --- | --- | --- | --- | --- | --- | --- | --- | --- | --- | --- |
|  |  | **(Chr_Pos)** |  |  |  | **estimate** | **allele/Null** | **allele/Null** | **correspondence** |  |  |
| *Xoc* | BAI5 | **S4_31728040** | 1.1E-14 | 2.3E-11 | 23.2 | 16.4 | C/G | 256/32 | *** | LOC_Os04g53260 | polyphenol oxidase, putative, expressed |
| *Xoc* | MAI10 | **S4_31728040** | 2.7E-05 | 5.6E-02 | 7.6 | 3.9 | C/G | 230/26 | * | LOC_Os04g53260 | polyphenol oxidase, putative, expressed |
| *Xoc* | MAI67 | **S4_31728040** | 4.4E-06 | 6.3E-03 | 9.6 | 2.6 | C/G | 219/29 | *** | LOC_Os04g53260 | polyphenol oxidase, putative, expressed |
| *Xoc* | MAI77 | **S4_31728040** | 1.1E-04 | 1.6E-01 | 5.9 | 3.2 | C/G | 217/28 | * | LOC_Os04g53260 | polyphenol oxidase, putative, expressed |
| *Xoc* | MAI123 | **S4_31728040** | 3.8E-04 | 5.2E-01 | 5.1 | 3.0 | C/G | 210/24 | * | LOC_Os04g53260 | polyphenol oxidase, putative, expressed |
| *Xoc* | MAI139 | **S4_31728040** | 3.2E-05 | 1.7E-01 | 7.8 | 5.2 | C/G | 194/27 | * | LOC_Os04g53260 | polyphenol oxidase, putative, expressed |
| *Xoo* | BAI3 | **S4_31728040** | 1.1E-12 | 1.8E-09 | 19.5 | 9.7 | C/G | 260/31 | *** | LOC_Os04g53260 | polyphenol oxidase, putative, expressed |
| *Xoo* | CFBP1951 | **S4_31728040** | 5.3E-05 | 6.2E-02 | 6.4 | 1.4 | C/G | 219/29 | * | LOC_Os04g53260 | polyphenol oxidase, putative, expressed |
| *Xoo* | MAI70 | **S4_31728040** | 5.6E-08 | 1.0E-04 | 11.4 | 7.1 | C/G | 243/31 | *** | LOC_Os04g53260 | polyphenol oxidase, putative, expressed |
| *Xoo* | MAI101 | **S4_31728040** | 1.7E-05 | 2.1E-02 | 7.9 | 5.4 | C/G | 211/27 | *** | LOC_Os04g53260 | polyphenol oxidase, putative, expressed |
| *Xoo* | MAI130 | **S4_31728040** | 4.7E-06 | 6.7E-03 | 8.0 | 3.6 | C/G | 233/30 | *** | LOC_Os04g53260 | polyphenol oxidase, putative, expressed |
| *Xoo* | MAI133 | **S4_31728040** | 1.9E-04 | 3.4E-01 | 5.8 | 2.2 | C/G | 206/29 | * | LOC_Os04g53260 | polyphenol oxidase, putative, expressed |
| *Xoo* | MAI136 | **S4_31728040** | 2.4E-05 | 4.7E-02 | 8.0 | 4.3 | C/G | 215/29 | *** | LOC_Os04g53260 | polyphenol oxidase, putative, expressed |
| *Xoo* | MAI145 | **S4_31728040** | 5.1E-05 | 3.7E-02 | 6.2 | 4.0 | C/G | 222/30 | *** | LOC_Os04g53260 | polyphenol oxidase, putative, expressed |
| *Xoc* | BAI5 | **S4_31751408** | 2.6E-14 | 4.7E-11 | 22.2 | 16.4 | T/C | 256/33 | *** | LOC_Os04g53300 | polyphenol oxidase, putative, expressed |
| *Xoc* | MAI10 | **S4_31751408** | 4.3E-05 | 6.9E-02 | 7.3 | 3.8 | T/C | 230/27 | * | LOC_Os04g53300 | polyphenol oxidase, putative, expressed |
| *Xoc* | MAI46 | **S4_31751408** | 7.0E-04 | 5.4E-01 | 4.7 | 4.6 | T/C | 226/30 | * | LOC_Os04g53300 | polyphenol oxidase, putative, expressed |
| *Xoc* | MAI67 | **S4_31751408** | 1.3E-06 | 3.5E-03 | 9.4 | 2.6 | T/C | 223/31 | *** | LOC_Os04g53300 | polyphenol oxidase, putative, expressed |
| *Xoc* | MAI77 | **S4_31751408** | 2.2E-05 | 6.6E-02 | 7.4 | 3.5 | T/C | 224/29 | * | LOC_Os04g53300 | polyphenol oxidase, putative, expressed |
| *Xoc* | MAI139 | **S4_31751408** | 7.5E-05 | 1.7E-01 | 7.2 | 4.9 | T/C | 200/28 | * | LOC_Os04g53300 | polyphenol oxidase, putative, expressed |
| *Xoo* | BAI3 | **S4_31751408** | 4.6E-14 | 9.6E-11 | 20.9 | 10.5 | T/C | 260/32 | *** | LOC_Os04g53300 | polyphenol oxidase, putative, expressed |
| *Xoo* | CFBP1951 | **S4_31751408** | 6.1E-05 | 6.6E-02 | 6.3 | 1.4 | T/C | 221/31 | * | LOC_Os04g53300 | polyphenol oxidase, putative, expressed |
| *Xoo* | MAI70 | **S4_31751408** | 2.9E-08 | 6.0E-05 | 12.2 | 7.2 | T/C | 240/32 | *** | LOC_Os04g53300 | polyphenol oxidase, putative, expressed |
| *Xoo* | MAI101 | **S4_31751408** | 2.2E-05 | 2.1E-02 | 7.9 | 5.3 | T/C | 215/28 | *** | LOC_Os04g53300 | polyphenol oxidase, putative, expressed |
| *Xoo* | MAI130 | **S4_31751408** | 3.3E-06 | 5.3E-03 | 9.2 | 3.6 | T/C | 234/31 | *** | LOC_Os04g53300 | polyphenol oxidase, putative, expressed |
| *Xoo* | MAI133 | **S4_31751408** | 7.3E-04 | 3.4E-01 | 4.8 | 1.9 | T/C | 211/31 | * | LOC_Os04g53300 | polyphenol oxidase, putative, expressed |
| *Xoo* | MAI136 | **S4_31751408** | 2.6E-05 | 4.7E-02 | 8.4 | 4.2 | T/C | 219/31 | *** | LOC_Os04g53300 | polyphenol oxidase, putative, expressed |
| *Xoo* | MAI145 | **S4_31751408** | 2.3E-05 | 2.2E-02 | 7.9 | 4.2 | T/C | 227/31 | *** | LOC_Os04g53300 | polyphenol oxidase, putative, expressed |

**Continuation Table S5.**

| **Pathovar** | **Strain** | **Marker** | **p-value** | **q-value** | **R^2^** | **Effect** | **Effect** | **AILs with Effect** | **Significance** | **Locus** | **Annotation** |
| --- | --- | --- | --- | --- | --- | --- | --- | --- | --- | --- | --- |
|  |  | **(Chr_Pos)** |  |  |  | **estimate** | **allele/Null** | **allele/Null** | **correspondence** |  |  |
| *Xoc* | BAI5 | **S4_31778051** | 2.3E-15 | 7.6E-12 | 26.0 | 18.1 | C/T | 253/28 | *** | LOC_Os04g53360 | expressed protein |
| *Xoc* | MAI10 | **S4_31778051** | 9.3E-06 | 2.7E-02 | 7.8 | 4.4 | C/T | 228/23 | *** | LOC_Os04g53360 | expressed protein |
| *Xoc* | MAI67 | **S4_31778051** | 1.1E-06 | 3.5E-03 | 9.7 | 2.9 | C/T | 217/26 | *** | LOC_Os04g53360 | expressed protein |
| *Xoc* | MAI77 | **S4_31778051** | 5.4E-04 | 5.2E-01 | 4.6 | 3.0 | C/T | 218/25 | * | LOC_Os04g53360 | expressed protein |
| *Xoc* | MAI123 | **S4_31778051** | 6.6E-04 | 6.3E-01 | 4.8 | 3.0 | C/T | 208/22 | * | LOC_Os04g53360 | expressed protein |
| *Xoc* | MAI139 | **S4_31778051** | 1.1E-04 | 1.9E-01 | 7.8 | 5.2 | C/T | 196/23 | * | LOC_Os04g53360 | expressed protein |
| *Xoo* | BAI3 | **S4_31778051** | 6.9E-15 | 2.5E-11 | 24.7 | 11.5 | C/T | 256/27 | *** | LOC_Os04g53360 | expressed protein |
| *Xoo* | CFBP1951 | **S4_31778051** | 8.5E-05 | 7.0E-02 | 6.2 | 1.5 | C/T | 218/26 | * | LOC_Os04g53360 | expressed protein |
| *Xoo* | MAI70 | **S4_31778051** | 7.3E-09 | 2.1E-05 | 13.8 | 7.9 | C/T | 241/28 | *** | LOC_Os04g53360 | expressed protein |
| *Xoo* | MAI101 | **S4_31778051** | 2.1E-05 | 2.1E-02 | 7.7 | 5.4 | C/T | 211/25 | *** | LOC_Os04g53360 | expressed protein |
| *Xoo* | MAI130 | **S4_31778051** | 3.0E-06 | 5.3E-03 | 8.8 | 3.8 | C/T | 232/27 | *** | LOC_Os04g53360 | expressed protein |
| *Xoo* | MAI136 | **S4_31778051** | 7.0E-05 | 6.3E-02 | 6.8 | 4.3 | C/T | 217/26 | * | LOC_Os04g53360 | expressed protein |
| *Xoo* | MAI145 | **S4_31778051** | 4.4E-05 | 3.5E-02 | 7.5 | 4.4 | C/T | 222/26 | *** | LOC_Os04g53360 | expressed protein |
| *Xoc* | BAI5 | S4_31781274 | 9.0E-04 | 1.9E-01 | 3.6 | -5.6 | T/C | 240/64 | * | intergenic |  |
| *Xoc* | BAI5 | S4_31782187 | 8.7E-04 | 1.9E-01 | 3.9 | -5.6 | A/G | 228/62 | * | intergenic |  |
| *Xoc* | BAI5 | S4_31786637 | 4.6E-04 | 1.2E-01 | 4.1 | 5.7 | A/G | 61/240 | * | LOC_Os04g53370 | acyltransferase, putative, expressed |
| *Xoc* | BAI5 | **S4_31787320** | 1.7E-09 | 2.1E-06 | 12.9 | -9.0 | C/G | 113/183 | *** | LOC_Os04g53370 | acyltransferase, putative, expressed |
| *Xoc* | MAI46 | **S4_31787320** | 3.3E-04 | 3.4E-01 | 5.2 | -3.2 | C/G | 97/166 | * | LOC_Os04g53370 | acyltransferase, putative, expressed |
| *Xoc* | MAI67 | **S4_31787320** | 1.0E-04 | 1.2E-01 | 6.0 | -1.4 | C/G | 97/160 | * | LOC_Os04g53370 | acyltransferase, putative, expressed |
| *Xoo* | BAI3 | **S4_31787320** | 4.1E-04 | 6.6E-02 | 4.2 | -3.3 | C/G | 113/185 | * | LOC_Os04g53370 | acyltransferase, putative, expressed |
| *Xoo* | MAI130 | **S4_31787320** | 2.5E-04 | 1.9E-01 | 5.5 | -2.0 | C/G | 99/171 | * | LOC_Os04g53370 | acyltransferase, putative, expressed |
| *Xoc* | BAI5 | **S4_31800256** | 1.2E-04 | 3.2E-02 | 5.1 | 5.7 | G/A | 202/95 | ** | LOC_Os04g53380 | expressed protein |
| *Xoo* | BAI3 | **S4_31800256** | 1.1E-05 | 4.4E-03 | 6.6 | 4.2 | G/A | 204/96 | *** | LOC_Os04g53380 | expressed protein |

**Continuation Table S5.**

| **Pathovar** | **Strain** | **Marker** | **p-value** | **q-value** | **R^2^** | **Effect** | **Effect** | **AILs with Effect** | **Significance** | **Locus** | **Annotation** |
| --- | --- | --- | --- | --- | --- | --- | --- | --- | --- | --- | --- |
|  |  | **(Chr_Pos)** |  |  |  | **estimate** | **allele/Null** | **allele/Null** | **correspondence** |  |  |
| *Xoc* | BAI5 | **S4_31801219** | 4.9E-17 | 2.3E-13 | 26.0 | 18.7 | C/A | 281/30 | *** | LOC_Os04g53380 | expressed protein |
| *Xoc* | MAI10 | **S4_31801219** | 1.3E-05 | 3.2E-02 | 7.0 | 4.2 | C/A | 253/24 | *** | LOC_Os04g53380 | expressed protein |
| *Xoc* | MAI67 | **S4_31801219** | 2.1E-06 | 3.7E-03 | 8.6 | 2.7 | C/A | 240/28 | *** | LOC_Os04g53380 | expressed protein |
| *Xoc* | MAI77 | **S4_31801219** | 2.6E-05 | 6.6E-02 | 6.7 | 3.6 | C/A | 241/26 | * | LOC_Os04g53380 | expressed protein |
| *Xoc* | MAI123 | **S4_31801219** | 4.2E-04 | 5.2E-01 | 4.9 | 3.0 | C/A | 231/24 | * | LOC_Os04g53380 | expressed protein |
| *Xoc* | MAI139 | **S4_31801219** | 6.9E-05 | 1.7E-01 | 6.8 | 5.1 | C/A | 218/25 | * | LOC_Os04g53380 | expressed protein |
| *Xoo* | BAI3 | **S4_31801219** | 1.8E-16 | 8.4E-13 | 24.4 | 11.9 | C/A | 285/29 | *** | LOC_Os04g53380 | expressed protein |
| *Xoo* | CFBP1951 | **S4_31801219** | 4.6E-05 | 5.9E-02 | 6.3 | 1.5 | C/A | 241/28 | * | LOC_Os04g53380 | expressed protein |
| *Xoo* | MAI70 | **S4_31801219** | 5.6E-09 | 2.1E-05 | 12.2 | 7.9 | C/A | 266/29 | *** | LOC_Os04g53380 | expressed protein |
| *Xoo* | MAI101 | **S4_31801219** | 1.7E-05 | 2.1E-02 | 7.4 | 5.6 | C/A | 232/25 | *** | LOC_Os04g53380 | expressed protein |
| *Xoo* | MAI130 | **S4_31801219** | 1.7E-06 | 5.3E-03 | 8.4 | 3.9 | C/A | 255/28 | *** | LOC_Os04g53380 | expressed protein |
| *Xoo* | MAI136 | **S4_31801219** | 1.4E-05 | 4.7E-02 | 7.3 | 4.5 | C/A | 237/28 | *** | LOC_Os04g53380 | expressed protein |
| *Xoo* | MAI145 | **S4_31801219** | 3.3E-05 | 3.0E-02 | 6.6 | 4.3 | C/A | 246/28 | *** | LOC_Os04g53380 | expressed protein |
| *Xoc* | BAI5 | **S4_31803015** | 4.7E-09 | 5.2E-06 | 14.4 | -8.9 | G/A | 93/167 | *** | LOC_Os04g53390 | MrBTB2 - Bric-a-Brac, Tramtrack, Broad Complex BTB domain with Meprin and TRAF Homology MATH-related domain, expressed |
| *Xoc* | MAI46 | **S4_31803015** | 2.4E-04 | 2.9E-01 | 5.9 | -3.5 | G/A | 82/150 | * | LOC_Os04g53390 | MrBTB2 - Bric-a-Brac, Tramtrack, Broad Complex BTB domain with Meprin and TRAF Homology MATH-related domain, expressed |
| *Xoc* | MAI67 | **S4_31803015** | 6.3E-04 | 4.9E-01 | 5.3 | -1.3 | G/A | 82/144 | * | LOC_Os04g53390 | MrBTB2 - Bric-a-Brac, Tramtrack, Broad Complex BTB domain with Meprin and TRAF Homology MATH-related domain, expressed |
| *Xoc* | MAI77 | **S4_31803015** | 3.3E-04 | 3.7E-01 | 5.3 | -2.1 | G/A | 83/145 | * | LOC_Os04g53390 | MrBTB2 - Bric-a-Brac, Tramtrack, Broad Complex BTB domain with Meprin and TRAF Homology MATH-related domain, expressed |
| *Xoo* | BAI3 | **S4_31803015** | 6.1E-04 | 8.9E-02 | 4.7 | -3.3 | G/A | 92/170 | * | LOC_Os04g53390 | MrBTB2 - Bric-a-Brac, Tramtrack, Broad Complex BTB domain with Meprin and TRAF Homology MATH-related domain, expressed |
| *Xoo* | MAI70 | **S4_31803015** | 9.3E-04 | 5.2E-01 | 4.4 | -3.0 | G/A | 87/160 | * | LOC_Os04g53390 | MrBTB2 - Bric-a-Brac, Tramtrack, Broad Complex BTB domain with Meprin and TRAF Homology MATH-related domain, expressed |
| *Xoo* | MAI130 | **S4_31803015** | 6.4E-04 | 3.3E-01 | 4.9 | -1.9 | G/A | 84/153 | * | LOC_Os04g53390 | MrBTB2 - Bric-a-Brac, Tramtrack, Broad Complex BTB domain with Meprin and TRAF Homology MATH-related domain, expressed |

**Continuation Table S5.**

| **Pathovar** | **Strain** | **Marker** | **p-value** | **q-value** | **R^2^** | **Effect** | **Effect** | **AILs with Effect** | **Significance** | **Locus** | **Annotation** |
| --- | --- | --- | --- | --- | --- | --- | --- | --- | --- | --- | --- |
|  |  | **(Chr_Pos)** |  |  |  | **estimate** | **allele/Null** | **allele/Null** | **correspondence** |  |  |
| *Xoc* | BAI5 | **S4_31803018** | 3.2E-15 | 7.6E-12 | 27.6 | 17.6 | C/T | 233/31 | *** | LOC_Os04g53390 | MrBTB2 - Bric-a-Brac, Tramtrack, Broad Complex BTB domain with Meprin and TRAF Homology MATH-related domain, expressed |
| *Xoc* | MAI10 | **S4_31803018** | 8.6E-06 | 2.7E-02 | 8.3 | 4.3 | C/T | 211/25 | *** | LOC_Os04g53390 | MrBTB2 - Bric-a-Brac, Tramtrack, Broad Complex BTB domain with Meprin and TRAF Homology MATH-related domain, expressed |
| *Xoc* | MAI46 | **S4_31803018** | 8.2E-04 | 5.7E-01 | 4.9 | 4.7 | C/T | 206/28 | * | LOC_Os04g53390 | MrBTB2 - Bric-a-Brac, Tramtrack, Broad Complex BTB domain with Meprin and TRAF Homology MATH-related domain, expressed |
| *Xoc* | MAI67 | **S4_31803018** | 1.4E-06 | 3.5E-03 | 10.5 | 2.7 | C/T | 199/29 | *** | LOC_Os04g53390 | MrBTB2 - Bric-a-Brac, Tramtrack, Broad Complex BTB domain with Meprin and TRAF Homology MATH-related domain, expressed |
| *Xoc* | MAI77 | **S4_31803018** | 2.5E-05 | 6.6E-02 | 7.2 | 3.6 | C/T | 204/27 | * | LOC_Os04g53390 | MrBTB2 - Bric-a-Brac, Tramtrack, Broad Complex BTB domain with Meprin and TRAF Homology MATH-related domain, expressed |
| *Xoc* | MAI123 | **S4_31803018** | 1.8E-04 | 5.2E-01 | 6.1 | 3.2 | C/T | 191/25 | * | LOC_Os04g53390 | MrBTB2 - Bric-a-Brac, Tramtrack, Broad Complex BTB domain with Meprin and TRAF Homology MATH-related domain, expressed |
| *Xoc* | MAI139 | **S4_31803018** | 1.2E-04 | 1.9E-01 | 7.3 | 5.0 | C/T | 184/25 | * | LOC_Os04g53390 | MrBTB2 - Bric-a-Brac, Tramtrack, Broad Complex BTB domain with Meprin and TRAF Homology MATH-related domain, expressed |
| *Xoo* | BAI3 | **S4_31803018** | 3.9E-14 | 9.6E-11 | 24.8 | 10.9 | C/T | 236/30 | *** | LOC_Os04g53390 | MrBTB2 - Bric-a-Brac, Tramtrack, Broad Complex BTB domain with Meprin and TRAF Homology MATH-related domain, expressed |
| *Xoo* | CFBP1951 | **S4_31803018** | 3.3E-04 | 2.3E-01 | 7.1 | 1.3 | C/T | 202/29 | * | LOC_Os04g53390 | MrBTB2 - Bric-a-Brac, Tramtrack, Broad Complex BTB domain with Meprin and TRAF Homology MATH-related domain, expressed |
| *Xoo* | MAI70 | **S4_31803018** | 8.5E-09 | 2.1E-05 | 13.5 | 7.8 | C/T | 220/30 | *** | LOC_Os04g53390 | MrBTB2 - Bric-a-Brac, Tramtrack, Broad Complex BTB domain with Meprin and TRAF Homology MATH-related domain, expressed |
| *Xoo* | MAI101 | **S4_31803018** | 5.1E-05 | 3.3E-02 | 7.5 | 5.2 | C/T | 193/26 | *** | LOC_Os04g53390 | MrBTB2 - Bric-a-Brac, Tramtrack, Broad Complex BTB domain with Meprin and TRAF Homology MATH-related domain, expressed |
| *Xoo* | MAI130 | **S4_31803018** | 6.1E-07 | 4.8E-03 | 10.3 | 4.0 | C/T | 212/29 | *** | LOC_Os04g53390 | MrBTB2 - Bric-a-Brac, Tramtrack, Broad Complex BTB domain with Meprin and TRAF Homology MATH-related domain, expressed |
| *Xoo* | MAI136 | **S4_31803018** | 1.6E-04 | 1.1E-01 | 8.1 | 3.9 | C/T | 199/29 | * | LOC_Os04g53390 | MrBTB2 - Bric-a-Brac, Tramtrack, Broad Complex BTB domain with Meprin and TRAF Homology MATH-related domain, expressed |
| *Xoo* | MAI145 | **S4_31803018** | 4.4E-04 | 2.0E-01 | 5.8 | 3.6 | C/T | 207/29 | * | LOC_Os04g53390 | MrBTB2 - Bric-a-Brac, Tramtrack, Broad Complex BTB domain with Meprin and TRAF Homology MATH-related domain, expressed |

**Continuation Table S5.**

| **Pathovar** | **Strain** | **Marker** | **p-value** | **q-value** | **R^2^** | **Effect** | **Effect** | **AILs with Effect** | **Significance** | **Locus** | **Annotation** |
| --- | --- | --- | --- | --- | --- | --- | --- | --- | --- | --- | --- |
|  |  | **(Chr_Pos)** |  |  |  | **estimate** | **allele/Null** | **allele/Null** | **correspondence** |  |  |
| *Xoc* | BAI5 | **S4_31803029** | 3.0E-15 | 7.6E-12 | 27.7 | 17.6 | G/A | 231/31 | *** | LOC_Os04g53390 | MrBTB2 - Bric-a-Brac, Tramtrack, Broad Complex BTB domain with Meprin and TRAF Homology MATH-related domain, expressed |
| *Xoc* | MAI10 | **S4_31803029** | 8.7E-06 | 2.7E-02 | 8.3 | 4.3 | G/A | 209/25 | *** | LOC_Os04g53390 | MrBTB2 - Bric-a-Brac, Tramtrack, Broad Complex BTB domain with Meprin and TRAF Homology MATH-related domain, expressed |
| *Xoc* | MAI46 | **S4_31803029** | 9.0E-04 | 5.7E-01 | 4.8 | 4.7 | G/A | 204/28 | * | LOC_Os04g53390 | MrBTB2 - Bric-a-Brac, Tramtrack, Broad Complex BTB domain with Meprin and TRAF Homology MATH-related domain, expressed |
| *Xoc* | MAI67 | **S4_31803029** | 1.5E-06 | 3.5E-03 | 10.5 | 2.7 | G/A | 197/29 | *** | LOC_Os04g53390 | MrBTB2 - Bric-a-Brac, Tramtrack, Broad Complex BTB domain with Meprin and TRAF Homology MATH-related domain, expressed |
| *Xoc* | MAI77 | **S4_31803029** | 2.9E-05 | 6.6E-02 | 7.1 | 3.6 | G/A | 202/27 | * | LOC_Os04g53390 | MrBTB2 - Bric-a-Brac, Tramtrack, Broad Complex BTB domain with Meprin and TRAF Homology MATH-related domain, expressed |
| *Xoc* | MAI123 | **S4_31803029** | 1.9E-04 | 5.2E-01 | 6.0 | 3.2 | G/A | 189/25 | * | LOC_Os04g53390 | MrBTB2 - Bric-a-Brac, Tramtrack, Broad Complex BTB domain with Meprin and TRAF Homology MATH-related domain, expressed |
| *Xoc* | MAI139 | **S4_31803029** | 1.3E-04 | 1.9E-01 | 7.2 | 5.0 | G/A | 182/25 | * | LOC_Os04g53390 | MrBTB2 - Bric-a-Brac, Tramtrack, Broad Complex BTB domain with Meprin and TRAF Homology MATH-related domain, expressed |
| *Xoo* | BAI3 | **S4_31803029** | 4.0E-14 | 9.6E-11 | 24.9 | 10.9 | G/A | 234/30 | *** | LOC_Os04g53390 | MrBTB2 - Bric-a-Brac, Tramtrack, Broad Complex BTB domain with Meprin and TRAF Homology MATH-related domain, expressed |
| *Xoo* | CFBP1951 | **S4_31803029** | 3.2E-04 | 2.3E-01 | 7.2 | 1.3 | G/A | 200/29 | * | LOC_Os04g53390 | MrBTB2 - Bric-a-Brac, Tramtrack, Broad Complex BTB domain with Meprin and TRAF Homology MATH-related domain, expressed |
| *Xoo* | MAI70 | **S4_31803029** | 8.1E-09 | 2.1E-05 | 13.6 | 7.8 | G/A | 218/30 | *** | LOC_Os04g53390 | MrBTB2 - Bric-a-Brac, Tramtrack, Broad Complex BTB domain with Meprin and TRAF Homology MATH-related domain, expressed |
| *Xoo* | MAI101 | **S4_31803029** | 4.8E-05 | 3.3E-02 | 7.5 | 5.3 | G/A | 191/26 | *** | LOC_Os04g53390 | MrBTB2 - Bric-a-Brac, Tramtrack, Broad Complex BTB domain with Meprin and TRAF Homology MATH-related domain, expressed |
| *Xoo* | MAI130 | **S4_31803029** | 6.6E-07 | 4.8E-03 | 10.3 | 4.0 | G/A | 210/29 | *** | LOC_Os04g53390 | MrBTB2 - Bric-a-Brac, Tramtrack, Broad Complex BTB domain with Meprin and TRAF Homology MATH-related domain, expressed |
| *Xoo* | MAI136 | **S4_31803029** | 1.7E-04 | 1.1E-01 | 8.2 | 3.9 | G/A | 197/29 | * | LOC_Os04g53390 | MrBTB2 - Bric-a-Brac, Tramtrack, Broad Complex BTB domain with Meprin and TRAF Homology MATH-related domain, expressed |
| *Xoo* | MAI145 | **S4_31803029** | 4.8E-04 | 2.1E-01 | 5.7 | 3.6 | G/A | 205/29 | * | LOC_Os04g53390 | MrBTB2 - Bric-a-Brac, Tramtrack, Broad Complex BTB domain with Meprin and TRAF Homology MATH-related domain, expressed |

**Continuation Table S5.**

| **Pathovar** | **Strain** | **Marker** | **p-value** | **q-value** | **R^2^** | **Effect** | **Effect** | **AILs with Effect** | **Significance** | **Locus** | **Annotation** |
| --- | --- | --- | --- | --- | --- | --- | --- | --- | --- | --- | --- |
|  |  | **(Chr_Pos)** |  |  |  | **estimate** | **allele/Null** | **allele/Null** | **correspondence** |  |  |
| *Xoc* | BAI5 | **S4_31812050** | 2.1E-08 | 2.1E-05 | 10.6 | 16.0 | G/T | 296/17 | *** | LOC_Os04g53410 | MBTB7 - Bric-a-Brac, Tramtrack, Broad Complex BTB domain with Meprin and TRAF Homology MATH domain, expressed |
| *Xoc* | MAI139 | **S4_31812050** | 5.9E-04 | 5.6E-01 | 5.0 | 6.2 | G/T | 232/12 | * | LOC_Os04g53410 | MBTB7 - Bric-a-Brac, Tramtrack, Broad Complex BTB domain with Meprin and TRAF Homology MATH domain, expressed |
| *Xoc* | BAI5 | **S4_31819592** | 1.2E-17 | 1.7E-13 | 27.5 | 18.9 | G/T | 276/31 | *** | LOC_Os04g53440 | RNA recognition motif containing protein, putative, expressed |
| *Xoc* | MAI10 | **S4_31819592** | 5.2E-06 | 2.7E-02 | 7.7 | 4.4 | G/T | 249/25 | *** | LOC_Os04g53440 | RNA recognition motif containing protein, putative, expressed |
| *Xoc* | MAI46 | **S4_31819592** | 8.1E-04 | 5.7E-01 | 4.3 | 4.7 | G/T | 243/28 | * | LOC_Os04g53440 | RNA recognition motif containing protein, putative, expressed |
| *Xoc* | MAI67 | **S4_31819592** | 9.6E-07 | 3.5E-03 | 9.3 | 2.7 | G/T | 236/29 | *** | LOC_Os04g53440 | RNA recognition motif containing protein, putative, expressed |
| *Xoc* | MAI77 | **S4_31819592** | 1.4E-05 | 6.6E-02 | 7.2 | 3.7 | G/T | 238/27 | * | LOC_Os04g53440 | RNA recognition motif containing protein, putative, expressed |
| *Xoc* | MAI123 | **S4_31819592** | 2.6E-04 | 5.2E-01 | 5.3 | 3.1 | G/T | 227/25 | * | LOC_Os04g53440 | RNA recognition motif containing protein, putative, expressed |
| *Xoc* | MAI139 | **S4_31819592** | 3.4E-05 | 1.7E-01 | 7.4 | 5.3 | G/T | 214/26 | * | LOC_Os04g53440 | RNA recognition motif containing protein, putative, expressed |
| *Xoo* | BAI3 | **S4_31819592** | 4.6E-17 | 4.9E-13 | 25.4 | 12.0 | G/T | 280/30 | *** | LOC_Os04g53440 | RNA recognition motif containing protein, putative, expressed |
| *Xoo* | CFBP1951 | **S4_31819592** | 3.5E-05 | 5.5E-02 | 6.5 | 1.5 | G/T | 237/29 | * | LOC_Os04g53440 | RNA recognition motif containing protein, putative, expressed |
| *Xoo* | MAI70 | **S4_31819592** | 6.0E-09 | 2.1E-05 | 12.4 | 7.8 | G/T | 262/30 | *** | LOC_Os04g53440 | RNA recognition motif containing protein, putative, expressed |
| *Xoo* | MAI101 | **S4_31819592** | 1.3E-05 | 2.1E-02 | 7.6 | 5.6 | G/T | 228/26 | *** | LOC_Os04g53440 | RNA recognition motif containing protein, putative, expressed |
| *Xoo* | MAI130 | **S4_31819592** | 1.6E-06 | 5.3E-03 | 8.8 | 3.8 | G/T | 251/29 | *** | LOC_Os04g53440 | RNA recognition motif containing protein, putative, expressed |
| *Xoo* | MAI133 | **S4_31819592** | 8.2E-04 | 3.4E-01 | 4.5 | 2.0 | G/T | 222/29 | * | LOC_Os04g53440 | RNA recognition motif containing protein, putative, expressed |
| *Xoo* | MAI136 | **S4_31819592** | 1.3E-05 | 4.7E-02 | 7.7 | 4.5 | G/T | 233/29 | *** | LOC_Os04g53440 | RNA recognition motif containing protein, putative, expressed |
| *Xoo* | MAI145 | **S4_31819592** | 1.4E-05 | 1.6E-02 | 7.2 | 4.4 | G/T | 242/29 | *** | LOC_Os04g53440 | RNA recognition motif containing protein, putative, expressed |

**Continuation Table S5.**

| **Pathovar** | **Strain** | **Marker** | **p-value** | **q-value** | **R^2^** | **Effect** | **Effect** | **AILs with Effect** | **Significance** | **Locus** | **Annotation** |
| --- | --- | --- | --- | --- | --- | --- | --- | --- | --- | --- | --- |
|  |  | **(Chr_Pos)** |  |  |  | **estimate** | **allele/Null** | **allele/Null** | **correspondence** |  |  |
| *Xoc* | BAI5 | **S4_31956274** | 5.9E-09 | 6.2E-06 | 13.4 | 11.6 | C/T | 246/35 | *** | LOC_Os04g53600 | expressed protein |
| *Xoc* | MAI10 | **S4_31956274** | 6.4E-04 | 4.9E-01 | 4.7 | 3.0 | C/T | 220/29 | * | LOC_Os04g53600 | expressed protein |
| *Xoc* | MAI67 | **S4_31956274** | 6.3E-06 | 8.0E-03 | 8.2 | 2.4 | C/T | 209/33 | *** | LOC_Os04g53600 | expressed protein |
| *Xoc* | MAI77 | **S4_31956274** | 3.1E-04 | 3.7E-01 | 5.5 | 2.9 | C/T | 211/30 | * | LOC_Os04g53600 | expressed protein |
| *Xoc* | MAI139 | **S4_31956274** | 7.3E-04 | 6.2E-01 | 5.0 | 4.2 | C/T | 192/27 | * | LOC_Os04g53600 | expressed protein |
| *Xoo* | BAI3 | **S4_31956274** | 1.2E-10 | 1.6E-07 | 15.9 | 8.3 | C/T | 248/35 | *** | LOC_Os04g53600 | expressed protein |
| *Xoo* | CFBP1951 | **S4_31956274** | 5.7E-04 | 3.0E-01 | 5.0 | 1.2 | C/T | 210/32 | * | LOC_Os04g53600 | expressed protein |
| *Xoo* | MAI70 | **S4_31956274** | 3.5E-05 | 4.3E-02 | 6.9 | 5.2 | C/T | 234/33 | *** | LOC_Os04g53600 | expressed protein |
| *Xoo* | MAI101 | **S4_31956274** | 1.9E-04 | 8.9E-02 | 5.9 | 4.4 | C/T | 202/31 | * | LOC_Os04g53600 | expressed protein |
| *Xoo* | MAI130 | **S4_31956274** | 8.8E-05 | 8.0E-02 | 6.5 | 3.0 | C/T | 224/32 | * | LOC_Os04g53600 | expressed protein |
| *Xoo* | MAI136 | **S4_31956274** | 3.0E-05 | 4.8E-02 | 7.7 | 4.1 | C/T | 206/33 | *** | LOC_Os04g53600 | expressed protein |
| *Xoo* | MAI145 | **S4_31956274** | 6.8E-05 | 4.3E-02 | 6.6 | 3.8 | C/T | 214/33 | *** | LOC_Os04g53600 | expressed protein |
| *Xoc* | BAI5 | **S4_31962475** | 1.5E-10 | 2.1E-07 | 14.3 | 11.8 | G/A | 270/42 | *** | LOC_Os04g53612 | APO, putative, expressed |
| *Xoc* | MAI10 | **S4_31962475** | 8.0E-05 | 1.2E-01 | 5.6 | 3.3 | G/A | 245/33 | * | LOC_Os04g53612 | APO, putative, expressed |
| *Xoc* | MAI46 | **S4_31962475** | 5.8E-04 | 5.3E-01 | 4.6 | 4.3 | G/A | 240/35 | * | LOC_Os04g53612 | APO, putative, expressed |
| *Xoc* | MAI67 | **S4_31962475** | 1.5E-06 | 3.5E-03 | 8.8 | 2.4 | G/A | 232/37 | *** | LOC_Os04g53612 | APO, putative, expressed |
| *Xoc* | MAI77 | **S4_31962475** | 7.7E-05 | 1.3E-01 | 5.9 | 3.0 | G/A | 234/35 | * | LOC_Os04g53612 | APO, putative, expressed |
| *Xoc* | MAI123 | **S4_31962475** | 2.5E-04 | 5.2E-01 | 5.3 | 2.7 | G/A | 224/32 | * | LOC_Os04g53612 | APO, putative, expressed |
| *Xoc* | MAI139 | **S4_31962475** | 7.3E-05 | 1.7E-01 | 6.7 | 4.7 | G/A | 213/30 | * | LOC_Os04g53612 | APO, putative, expressed |
| *Xoo* | BAI3 | **S4_31962475** | 2.1E-11 | 3.1E-08 | 15.3 | 8.1 | G/A | 273/41 | *** | LOC_Os04g53612 | APO, putative, expressed |
| *Xoo* | CFBP1951 | **S4_31962475** | 8.3E-05 | 7.0E-02 | 5.9 | 1.3 | G/A | 234/36 | * | LOC_Os04g53612 | APO, putative, expressed |
| *Xoo* | MAI70 | **S4_31962475** | 4.3E-07 | 6.3E-04 | 9.2 | 6.0 | G/A | 258/38 | *** | LOC_Os04g53612 | APO, putative, expressed |
| *Xoo* | MAI101 | **S4_31962475** | 2.5E-05 | 2.2E-02 | 7.0 | 4.8 | G/A | 225/34 | *** | LOC_Os04g53612 | APO, putative, expressed |
| *Xoo* | MAI130 | **S4_31962475** | 1.8E-06 | 5.3E-03 | 8.6 | 3.4 | G/A | 248/36 | *** | LOC_Os04g53612 | APO, putative, expressed |
| *Xoo* | MAI133 | **S4_31962475** | 3.2E-04 | 3.4E-01 | 5.1 | 1.9 | G/A | 219/37 | * | LOC_Os04g53612 | APO, putative, expressed |
| *Xoo* | MAI136 | **S4_31962475** | 5.8E-06 | 4.2E-02 | 7.9 | 4.2 | G/A | 229/36 | *** | LOC_Os04g53612 | APO, putative, expressed |
| *Xoo* | MAI145 | **S4_31962475** | 4.5E-05 | 3.5E-02 | 6.3 | 3.7 | G/A | 239/37 | *** | LOC_Os04g53612 | APO, putative, expressed |

**Continuation Table S5.**

| **Pathovar** | **Strain** | **Marker** | **p-value** | **q-value** | **R^2^** | **Effect** | **Effect** | **AILs with Effect** | **Significance** | **Locus** | **Annotation** |
| --- | --- | --- | --- | --- | --- | --- | --- | --- | --- | --- | --- |
|  |  | **(Chr_Pos)** |  |  |  | **estimate** | **allele/Null** | **allele/Null** | **correspondence** |  |  |
| *Xoc* | BAI5 | **S4_31981005** | 2.3E-12 | 3.7E-09 | 17.7 | 13.6 | G/A | 268/38 | *** | LOC_Os04g53660 | transposon protein, putative, unclassified, expressed |
| *Xoc* | MAI10 | **S4_31981005** | 3.7E-05 | 6.6E-02 | 6.2 | 3.6 | G/A | 243/31 | * | LOC_Os04g53660 | transposon protein, putative, unclassified, expressed |
| *Xoc* | MAI46 | **S4_31981005** | 4.5E-04 | 4.4E-01 | 4.8 | 4.5 | G/A | 236/34 | * | LOC_Os04g53660 | transposon protein, putative, unclassified, expressed |
| *Xoc* | MAI67 | **S4_31981005** | 1.8E-06 | 3.6E-03 | 8.8 | 2.4 | G/A | 228/36 | *** | LOC_Os04g53660 | transposon protein, putative, unclassified, expressed |
| *Xoc* | MAI77 | **S4_31981005** | 6.1E-05 | 1.2E-01 | 6.3 | 3.1 | G/A | 232/33 | * | LOC_Os04g53660 | transposon protein, putative, unclassified, expressed |
| *Xoc* | MAI123 | **S4_31981005** | 2.4E-04 | 5.2E-01 | 5.4 | 2.8 | G/A | 221/32 | * | LOC_Os04g53660 | transposon protein, putative, unclassified, expressed |
| *Xoc* | MAI139 | **S4_31981005** | 5.4E-05 | 1.7E-01 | 7.0 | 4.8 | G/A | 210/30 | * | LOC_Os04g53660 | transposon protein, putative, unclassified, expressed |
| *Xoo* | BAI3 | **S4_31981005** | 1.2E-13 | 2.2E-10 | 19.2 | 9.3 | G/A | 271/38 | *** | LOC_Os04g53660 | transposon protein, putative, unclassified, expressed |
| *Xoo* | CFBP1951 | **S4_31981005** | 6.5E-05 | 6.6E-02 | 6.1 | 1.3 | G/A | 231/35 | * | LOC_Os04g53660 | transposon protein, putative, unclassified, expressed |
| *Xoo* | MAI70 | **S4_31981005** | 2.5E-07 | 4.0E-04 | 9.6 | 6.3 | G/A | 255/36 | *** | LOC_Os04g53660 | transposon protein, putative, unclassified, expressed |
| *Xoo* | MAI101 | **S4_31981005** | 1.3E-05 | 2.1E-02 | 7.6 | 5.0 | G/A | 222/33 | *** | LOC_Os04g53660 | transposon protein, putative, unclassified, expressed |
| *Xoo* | MAI130 | **S4_31981005** | 2.6E-06 | 5.3E-03 | 8.6 | 3.4 | G/A | 244/35 | *** | LOC_Os04g53660 | transposon protein, putative, unclassified, expressed |
| *Xoo* | MAI133 | **S4_31981005** | 1.6E-04 | 3.4E-01 | 5.6 | 2.0 | G/A | 216/36 | * | LOC_Os04g53660 | transposon protein, putative, unclassified, expressed |
| *Xoo* | MAI136 | **S4_31981005** | 1.2E-06 | 1.8E-02 | 9.2 | 4.5 | G/A | 226/36 | *** | LOC_Os04g53660 | transposon protein, putative, unclassified, expressed |
| *Xoo* | MAI145 | **S4_31981005** | 6.2E-05 | 4.1E-02 | 6.2 | 3.7 | G/A | 236/36 | *** | LOC_Os04g53660 | transposon protein, putative, unclassified, expressed |
| *Xoc* | MAI46 | S4_31992079 | 6.7E-04 | 5.4E-01 | 5.1 | -4.2 | T/C | 200/38 | * | LOC_Os04g53670 | expressed protein |
| *Xoo* | MAI130 | S4_32007187 | 3.0E-04 | 2.0E-01 | 4.6 | 2.9 | A/G | 28/261 | * | LOC_Os04g53700 | zinc finger protein, putative, expressed |
| *Xoo* | BAI3 | S4_32134600 | 5.5E-04 | 8.7E-02 | 4.0 | 3.7 | C/A | 239/64 | * | LOC_Os04g53920 | leucoanthocyanidin reductase, putative, expressed |
| *Xoc* | BAI5 | S4_32136574 | 4.8E-06 | 1.7E-03 | 7.6 | 13.7 | T/C | 260/15 | *** | intergenic |  |
| *Xoo* | BAI3 | S4_32142089 | 9.8E-04 | 1.3E-01 | 3.5 | 5.2 | G/T | 293/21 | * | LOC_Os04g53940 | hypothetical protein |
| *Xoc* | BAI5 | **S4_32181189** | 8.0E-04 | 1.8E-01 | 3.7 | 7.7 | T/G | 290/24 | * | LOC_Os04g53998 | kinase, putative, expressed |
| *Xoo* | BAI3 | **S4_32181189** | 5.7E-04 | 8.7E-02 | 3.8 | 5.2 | T/G | 293/23 | * | LOC_Os04g53998 | kinase, putative, expressed |
| *Xoo* | MAI70 | **S4_32181189** | 9.2E-04 | 5.2E-01 | 3.8 | 4.9 | T/G | 278/23 | * | LOC_Os04g53998 | kinase, putative, expressed |
| *Xoc* | BAI5 | S4_32189350 | 1.7E-07 | 9.7E-05 | 10.0 | 14.5 | C/T | 268/17 | *** | intergenic |  |

**Continuation Table S5.**

| **Pathovar** | **Strain** | **Marker** | **p-value** | **q-value** | **R^2^** | **Effect** | **Effect** | **AILs with Effect** | **Significance** | **Locus** | **Annotation** |
| --- | --- | --- | --- | --- | --- | --- | --- | --- | --- | --- | --- |
|  |  | **(Chr_Pos)** |  |  |  | **estimate** | **allele/Null** | **allele/Null** | **correspondence** |  |  |
| *Xoo* | MAI130 | S4_32193486 | 6.6E-04 | 3.3E-01 | 4.1 | 2.7 | A/G | 28/260 | * | LOC_Os04g54002 | serine/threonine-protein kinase receptor precursor, putative, expressed |
| *Xoo* | MAI136 | S4_32234992 | 6.3E-04 | 2.6E-01 | 4.9 | 3.7 | A/G | 230/25 | * | LOC_Os04g54090 | RALFL18 - Rapid ALkalinization Factor RALF family protein precursor, expressed |
| *Xoc* | BAI5 | S4_32297823 | 7.5E-04 | 1.7E-01 | 3.7 | -5.7 | T/C | 238/67 | * | LOC_Os04g54230 | wound induced protein, putative, expressed |
| *Xoc* | BAI5 | S4_32367097 | 9.3E-04 | 1.9E-01 | 3.5 | -5.6 | C/G | 241/67 | * | LOC_Os04g54400 | BTBN12 - Bric-a-Brac, Tramtrack, Broad Complex BTB domain with non-phototropic hypocotyl 3 NPH3 and coiled-coil domains, expressed |
| *Xoc* | BAI5 | S4_32379663 | 1.5E-06 | 6.7E-04 | 8.7 | 13.4 | T/G | 264/17 | *** | LOC_Os04g54420 | protein of unknown function, DUF618 domain containing protein, expressed |
| *Xoc* | BAI5 | S4_32546106 | 4.3E-06 | 1.6E-03 | 7.2 | 12.2 | C/G | 285/18 | *** | LOC_Os04g54720 | expressed protein |
| *Xoc* | BAI5 | S4_32546110 | 4.3E-06 | 1.6E-03 | 7.2 | 12.2 | C/A | 285/18 | *** | LOC_Os04g54720 | expressed protein |
| *Xoc* | BAI5 | **S4_32591580** | 4.8E-04 | 1.2E-01 | 4.1 | 8.3 | A/C | 280/23 | * | LOC_Os04g54810 | beta-D-xylosidase, putative, expressed |
| *Xoo* | BAI3 | **S4_32591580** | 7.5E-04 | 1.0E-01 | 3.8 | 5.2 | A/C | 283/22 | * | LOC_Os04g54810 | beta-D-xylosidase, putative, expressed |
| *Xoo* | MAI70 | **S4_32591580** | 9.9E-04 | 5.3E-01 | 4.3 | 5.0 | A/C | 268/22 | * | LOC_Os04g54810 | beta-D-xylosidase, putative, expressed |
| *Xoc* | BAI5 | S4_32593050 | 7.6E-04 | 1.7E-01 | 3.7 | 7.8 | C/T | 290/24 | * | LOC_Os04g54810 | beta-D-xylosidase, putative, expressed |
| *Xoc* | BAI5 | **S4_32593833** | 5.6E-04 | 1.4E-01 | 3.9 | 8.2 | T/A | 288/23 | * | LOC_Os04g54810 | beta-D-xylosidase, putative, expressed |
| *Xoo* | BAI3 | **S4_32593833** | 6.9E-04 | 9.7E-02 | 3.7 | 5.2 | T/A | 291/22 | * | LOC_Os04g54810 | beta-D-xylosidase, putative, expressed |
| *Xoo* | MAI70 | **S4_32593833** | 6.8E-04 | 4.3E-01 | 4.0 | 5.2 | T/A | 276/22 | * | LOC_Os04g54810 | beta-D-xylosidase, putative, expressed |
| *Xoc* | BAI5 | **S4_32595542** | 4.7E-04 | 1.2E-01 | 4.0 | 8.3 | C/T | 290/23 | * | LOC_Os04g54810 | beta-D-xylosidase, putative, expressed |
| *Xoo* | BAI3 | **S4_32595542** | 2.8E-04 | 4.7E-02 | 4.2 | 5.6 | C/T | 293/22 | ** | LOC_Os04g54810 | beta-D-xylosidase, putative, expressed |
| *Xoo* | MAI70 | **S4_32595542** | 9.4E-04 | 5.2E-01 | 3.7 | 5.0 | C/T | 278/22 | * | LOC_Os04g54810 | beta-D-xylosidase, putative, expressed |
| *Xoo* | MAI130 | S4_32627084 | 2.9E-04 | 2.0E-01 | 5.0 | 2.1 | A/G | 217/66 | * | LOC_Os04g54850 | pectinesterase, putative, expressed |
| *Xoo* | MAI130 | S4_33325361 | 5.1E-04 | 3.1E-01 | 4.2 | 2.0 | T/C | 73/208 | * | LOC_Os04g55950 | expressed protein |
| *Xoo* | MAI130 | S4_33336957 | 8.8E-04 | 4.0E-01 | 4.2 | 1.9 | T/G | 74/201 | * | intergenic |  |
| *Xoc* | MAI77 | S4_33555508 | 3.1E-04 | 3.7E-01 | 5.9 | 2.3 | G/T | 54/180 | * | intergenic |  |
| *Xoc* | MAI77 | S4_33600912 | 6.1E-04 | 5.2E-01 | 4.5 | 2.1 | G/A | 58/204 | * | intergenic |  |
|  |  |  |  |  |  |  |  |  |  |  |  |
| *Xoc* | MAI61 | S5_69377 | 6.6E-04 | 9.1E-01 | 4.7 | -3.4 | T/C | 221/20 | * | LOC_Os05g01040 | serine/threonine-protein kinase, putative, expressed |
| *Xoc* | MAI61 | **S5_103237** | 6.8E-04 | 9.1E-01 | 4.6 | 2.2 | C/T | 53/200 | * | LOC_Os05g01120 | cytochrome P450, putative, expressed |
| *Xoo* | BAI3 | **S5_103237** | 4.3E-05 | 1.2E-02 | 5.7 | -4.4 | C/T | 60/244 | *** | LOC_Os05g01120 | cytochrome P450, putative, expressed |
| *Xoo* | BAI3 | S5_227187 | 5.5E-09 | 6.1E-06 | 11.9 | -8.1 | A/C | 34/276 | *** | LOC_Os05g01370 | polygalacturonase inhibitor precursor, putative, expressed |
| *Xoc* | MAI61 | S5_231622 | 3.0E-04 | 9.1E-01 | 5.1 | -3.4 | C/T | 225/22 | * | LOC_Os05g01380 | polygalacturonase inhibitor precursor, putative, expressed |

**Continuation Table S5.**

| **Pathovar** | **Strain** | **Marker** | **p-value** | **q-value** | **R^2^** | **Effect** | **Effect** | **AILs with Effect** | **Significance** | **Locus** | **Annotation** |
| --- | --- | --- | --- | --- | --- | --- | --- | --- | --- | --- | --- |
|  |  | **(Chr_Pos)** |  |  |  | **estimate** | **allele/Null** | **allele/Null** | **correspondence** |  |  |
| *Xoc* | MAI61 | S5_251710 | 8.4E-04 | 9.1E-01 | 4.4 | -3.3 | G/A | 228/20 | * | LOC_Os05g01440 | phosphoribosylformylglycinamidine synthase, putative, expressed |
| *Xoo* | BAI3 | S5_285834 | 4.0E-08 | 4.2E-05 | 10.3 | -7.9 | A/G | 31/283 | *** | LOC_Os05g01480 | ras-related protein, putative, expressed |
| *Xoo* | BAI3 | S5_347328 | 1.5E-04 | 3.3E-02 | 4.9 | -4.1 | G/A | 60/245 | ** | LOC_Os05g01580 | integral membrane protein DUF6 containing protein, expressed |
| *Xoo* | BAI3 | **S5_353165** | 1.6E-09 | 1.9E-06 | 12.3 | -8.2 | T/C | 36/275 | *** | LOC_Os05g01590 | heat shock protein DnaJ, putative, expressed |
| *Xoo* | MAI133 | **S5_353165** | 9.8E-04 | 3.7E-01 | 4.3 | -1.9 | T/C | 32/224 | * | LOC_Os05g01590 | heat shock protein DnaJ, putative, expressed |
| *Xoo* | BAI3 | S5_440644 | 2.1E-06 | 1.2E-03 | 7.8 | -6.6 | A/G | 34/266 | *** | LOC_Os05g01710 | transcription initiation factor IIA gamma chain, putative, expressed |
| *Xoo* | BAI3 | S5_453169 | 8.4E-08 | 8.0E-05 | 9.6 | 7.6 | G/A | 284/33 | *** | LOC_Os05g01750 | TruB family pseudouridylate synthase, putative, expressed |
| *Xoo* | BAI3 | S5_574926 | 2.1E-06 | 1.2E-03 | 7.6 | -6.4 | T/C | 36/269 | *** | LOC_Os05g02010 | expressed protein |
| *Xoo* | BAI3 | S5_849335 | 2.5E-05 | 8.4E-03 | 5.9 | -6.4 | T/C | 25/287 | *** | LOC_Os05g02480 | expressed protein |
| *Xoo* | BAI3 | **S5_850180** | 1.1E-06 | 7.3E-04 | 8.0 | -6.8 | C/G | 33/280 | *** | LOC_Os05g02480 | expressed protein |
| *Xoo* | MAI133 | **S5_850180** | 7.9E-04 | 3.4E-01 | 4.4 | -2.0 | C/G | 28/228 | * | LOC_Os05g02480 | expressed protein |
| *Xoo* | BAI3 | S5_904372 | 1.9E-04 | 3.7E-02 | 4.6 | -4.2 | T/G | 51/247 | ** | LOC_Os05g02590 | transferase family protein, putative, expressed |
| *Xoo* | BAI3 | S5_934093 | 1.3E-05 | 5.1E-03 | 7.5 | -6.6 | A/T | 27/233 | *** | LOC_Os05g02650 | expressed protein |
| *Xoo* | BAI3 | S5_1200961 | 6.5E-05 | 1.7E-02 | 5.2 | -5.4 | A/G | 35/280 | *** | LOC_Os05g03070 | transporter, putative, expressed |
| *Xoo* | BAI3 | S5_1200964 | 6.5E-05 | 1.7E-02 | 5.2 | -5.4 | C/G | 35/282 | *** | LOC_Os05g03070 | transporter, putative, expressed |
| *Xoo* | BAI3 | S5_1200971 | 6.5E-05 | 1.7E-02 | 5.2 | -5.4 | T/G | 35/282 | *** | LOC_Os05g03070 | transporter, putative, expressed |
| *Xoo* | BAI3 | S5_1224178 | 7.7E-05 | 1.9E-02 | 5.1 | -5.3 | A/G | 34/282 | *** | LOC_Os05g03100 | HECT-domain domain containing protein, expressed |
| *Xoo* | BAI3 | S5_1258051 | 6.3E-04 | 9.0E-02 | 3.9 | -3.3 | G/T | 69/242 | * | LOC_Os05g03140 | tetraspanin family protein, putative, expressed |
| *Xoo* | BAI3 | S5_1320280 | 5.6E-04 | 8.7E-02 | 3.9 | 3.4 | G/A | 241/67 | * | intergenic |  |
| *Xoc* | MAI46 | S5_1475022 | 1.2E-04 | 2.9E-01 | 5.8 | -3.5 | C/A | 168/96 | * | LOC_Os05g03480 | acyl-coenzyme A dehydrogenase, mitochondrial precursor, putative, expressed |
| *Xoc* | MAI46 | S5_1475065 | 9.0E-04 | 5.7E-01 | 4.6 | -2.9 | C/T | 127/122 | * | LOC_Os05g03480 | acyl-coenzyme A dehydrogenase, mitochondrial precursor, putative, expressed |
| *Xoo* | CFBP1951 | S5_3057869 | 5.1E-04 | 3.0E-01 | 5.3 | -1.0 | G/A | 56/171 | * | LOC_Os05g06120 | ubiquitin conjugating enzyme protein, putative, expressed |
| *Xoo* | CFBP1951 | S5_7731832 | 2.3E-04 | 1.8E-01 | 5.2 | -1.6 | C/T | 242/18 | * | LOC_Os05g13904 | expressed protein |
| *Xoo* | MAI134 | S5_21351387 | 1.5E-06 | 2.1E-02 | 10.3 | -2.7 | C/T | 260/14 | *** | intergenic |  |
| *Xoo* | MAI72 | S5_29597255 | 6.3E-04 | 2.7E-01 | 4.5 | 1.3 | G/T | 171/83 | * | LOC_Os05g51620 | expressed protein |

**Continuation Table S5.**

| **Pathovar** | **Strain** | **Marker** | **p-value** | **q-value** | **R^2^** | **Effect** | **Effect** | **AILs with Effect** | **Significance** | **Locus** | **Annotation** |
| --- | --- | --- | --- | --- | --- | --- | --- | --- | --- | --- | --- |
|  |  | **(Chr_Pos)** |  |  |  | **estimate** | **allele/Null** | **allele/Null** | **correspondence** |  |  |
| *Xoo* | MAI101 | S6_5631365 | 6.2E-04 | 1.7E-01 | 4.6 | -4.6 | T/G | 234/24 | * | LOC_Os06g10780 | AP2 domain containing protein, expressed |
| *Xoo* | MAI101 | S6_5631366 | 6.2E-04 | 1.7E-01 | 4.6 | -4.6 | T/C | 234/24 | * | LOC_Os06g10780 | AP2 domain containing protein, expressed |
| *Xoo* | MAI101 | S6_5631369 | 5.9E-04 | 1.7E-01 | 4.6 | -4.6 | C/G | 235/24 | * | LOC_Os06g10780 | AP2 domain containing protein, expressed |
| *Xoo* | MAI101 | S6_5631372 | 5.9E-04 | 1.7E-01 | 4.6 | -4.6 | G/T | 235/24 | * | LOC_Os06g10780 | AP2 domain containing protein, expressed |
| *Xoo* | MAI101 | S6_5631375 | 5.9E-04 | 1.7E-01 | 4.6 | -4.6 | T/A | 235/24 | * | LOC_Os06g10780 | AP2 domain containing protein, expressed |
| *Xoo* | MAI101 | S6_5658039 | 2.5E-04 | 1.1E-01 | 5.2 | -4.9 | T/G | 239/24 | * | LOC_Os06g10850 | lipase, putative, expressed |
| *Xoc* | MAI123 | S6_28685790 | 7.5E-04 | 6.3E-01 | 5.1 | -1.8 | T/C | 120/98 | * | LOC_Os06g47320 | T-complex protein, putative, expressed |
| *Xoc* | MAI46 | S6_29789433 | 7.5E-05 | 2.9E-01 | 6.1 | -7.1 | A/C | 251/17 | * | LOC_Os06g49160 | thylakoid lumenal 16.5 kDa protein, chloroplast precursor, putative, expressed |
| *Xoc* | MAI77 | S6_29827098 | 7.6E-04 | 6.0E-01 | 4.2 | -3.3 | A/G | 248/19 | * | intergenic |  |
| *Xoc* | MAI77 | S6_29827171 | 8.6E-04 | 6.4E-01 | 4.2 | -3.3 | A/T | 247/19 | * | intergenic |  |
| *Xoc* | MAI46 | S6_29851557 | 2.0E-04 | 2.9E-01 | 5.4 | -4.0 | A/C | 222/54 | * | LOC_Os06g49260 | OsWAK65 - OsWAK receptor-like protein kinase, expressed |
| *Xoc* | MAI46 | S6_29892576 | 2.4E-04 | 2.9E-01 | 5.4 | -4.0 | T/C | 208/53 | * | LOC_Os06g49340 | OsFBDUF35 - F-box and DUF domain containing protein, expressed |
|  |  |  |  |  |  |  |  |  |  |  |  |
| *Xoo* | CFBP1951 | S7_5956894 | 4.9E-12 | 6.8E-08 | 20.6 | -2.0 | G/A | 209/50 | *** | intergenic |  |
| *Xoo* | CFBP1951 | S7_5993972 | 6.6E-11 | 4.6E-07 | 17.9 | -1.8 | C/T | 183/62 | *** | LOC_Os07g10940 | exo70 exocyst complex subunit family protein, expressed |
| *Xoo* | CFBP1951 | S7_5994097 | 7.4E-10 | 3.5E-06 | 15.5 | -1.6 | C/T | 192/65 | *** | LOC_Os07g10940 | exo70 exocyst complex subunit family protein, expressed |
| *Xoo* | CFBP1951 | S7_7043661 | 6.7E-06 | 1.9E-02 | 7.9 | -1.2 | T/C | 191/63 | *** | intergenic |  |
| *Xoo* | CFBP1951 | S7_7043696 | 1.9E-05 | 3.4E-02 | 7.1 | -1.1 | C/T | 202/64 | *** | intergenic |  |
| *Xoo* | CFBP1951 | S7_7047227 | 4.1E-05 | 5.8E-02 | 7.0 | -1.1 | A/G | 173/58 | * | LOC_Os07g12380 | hypothetical protein |
| *Xoo* | CFBP1951 | S7_7047284 | 5.5E-04 | 3.0E-01 | 5.8 | -0.9 | G/A | 175/59 | * | LOC_Os07g12380 | hypothetical protein |
| *Xoo* | CFBP1951 | S7_7047325 | 5.5E-04 | 3.0E-01 | 5.8 | -0.9 | C/T | 175/59 | * | LOC_Os07g12380 | hypothetical protein |
| *Xoo* | CFBP1951 | S7_7047333 | 1.2E-05 | 2.4E-02 | 7.5 | -1.2 | T/C | 201/63 | *** | LOC_Os07g12380 | hypothetical protein |
| *Xoo* | CFBP1951 | S7_7047343 | 1.2E-05 | 2.4E-02 | 7.5 | -1.2 | G/A | 201/63 | *** | LOC_Os07g12380 | hypothetical protein |
| *Xoo* | CFBP1951 | S7_7182262 | 3.9E-04 | 2.4E-01 | 7.1 | -1.2 | G/A | 204/32 | * | LOC_Os07g12580 | expressed protein |
| *Xoo* | CFBP1951 | S7_7182273 | 3.9E-04 | 2.4E-01 | 7.1 | -1.2 | G/A | 204/32 | * | LOC_Os07g12580 | expressed protein |
| *Xoo* | CFBP1951 | S7_7186425 | 1.5E-06 | 5.1E-03 | 9.1 | -1.3 | C/G | 199/64 | *** | LOC_Os07g12590 | OsFBX225 - F-box domain containing protein, expressed |
| *Xoc* | MAI123 | S7_18859878 | 7.3E-04 | 6.3E-01 | 4.9 | -3.4 | G/A | 236/17 | * | intergenic |  |
| *Xoc* | MAI123 | S7_18859880 | 7.3E-04 | 6.3E-01 | 4.9 | -3.4 | C/T | 236/17 | * | intergenic |  |

**Continuation Table S5.**

| **Pathovar** | **Strain** | **Marker** | **p-value** | **q-value** | **R^2^** | **Effect** | **Effect** | **AILs with Effect** | **Significance** | **Locus** | **Annotation** |
| --- | --- | --- | --- | --- | --- | --- | --- | --- | --- | --- | --- |
|  |  | **(Chr_Pos)** |  |  |  | **estimate** | **allele/Null** | **allele/Null** | **correspondence** |  |  |
| *Xoo* | MAI136 | S7_23962559 | 3.0E-04 | 1.7E-01 | 5.4 | -4.0 | A/C | 200/40 | * | intergenic |  |
| *Xoo* | MAI101 | S7_24584432 | 2.6E-04 | 1.1E-01 | 5.9 | -5.0 | G/A | 190/40 | * | LOC_Os07g41080 | hydrolase, alpha/beta fold family domain containing protein, expressed |
| *Xoo* | MAI72 | S7_26755135 | 7.3E-04 | 2.7E-01 | 4.3 | -1.2 | A/T | 179/95 | * | LOC_Os07g44820 | expressed protein |
| *Xoo* | MAI72 | S7_26819778 | 4.7E-04 | 2.7E-01 | 4.7 | -1.3 | A/G | 185/90 | * | LOC_Os07g44970 | XPA-binding protein 2, putative, expressed |
| *Xoo* | MAI72 | S7_26916718 | 9.9E-05 | 1.9E-01 | 5.7 | -1.4 | A/G | 177/91 | * | LOC_Os07g45090 | NADH-ubiquinone oxidoreductase 51 kDa subunit, mitochondrial precursor, putative, expressed |
| *Xoo* | MAI72 | S7_26923436 | 1.2E-04 | 1.9E-01 | 5.6 | -1.4 | C/G | 182/92 | * | LOC_Os07g45100 | endonuclease/exonuclease/phosphatase family domain containing protein, expressed |
| *Xoo* | MAI72 | S7_27060318 | 7.6E-04 | 2.7E-01 | 4.3 | -1.3 | C/G | 186/86 | * | LOC_Os07g45370 | expressed protein |
| *Xoo* | MAI72 | S7_27060573 | 5.3E-04 | 2.7E-01 | 4.5 | -1.3 | A/G | 187/86 | * | LOC_Os07g45370 | expressed protein |
| *Xoo* | MAI72 | S7_27060806 | 6.0E-04 | 2.7E-01 | 4.5 | -1.3 | C/A | 188/83 | * | LOC_Os07g45370 | expressed protein |
| *Xoo* | MAI72 | S7_27135983 | 6.0E-04 | 2.7E-01 | 4.4 | -1.3 | A/T | 185/88 | * | LOC_Os07g45480 | expressed protein |
| *Xoo* | MAI72 | S7_27135987 | 6.0E-04 | 2.7E-01 | 4.4 | -1.3 | G/A | 186/88 | * | LOC_Os07g45480 | expressed protein |
| *Xoo* | MAI72 | S7_27142637 | 3.5E-04 | 2.7E-01 | 4.8 | -1.4 | A/T | 185/78 | * | LOC_Os07g45490 | expressed protein |
| *Xoo* | MAI72 | S7_27142664 | 3.5E-04 | 2.7E-01 | 4.8 | -1.4 | T/G | 185/78 | * | LOC_Os07g45490 | expressed protein |
| *Xoo* | MAI72 | S7_27142675 | 3.5E-04 | 2.7E-01 | 4.8 | -1.4 | T/A | 185/78 | * | LOC_Os07g45490 | expressed protein |
| *Xoo* | MAI72 | S7_27142677 | 3.5E-04 | 2.7E-01 | 4.8 | -1.4 | A/G | 185/78 | * | LOC_Os07g45490 | expressed protein |
| *Xoo* | MAI72 | S7_27479752 | 6.7E-04 | 2.7E-01 | 4.4 | -1.3 | G/A | 186/88 | * | LOC_Os07g46039 | expressed protein |
| *Xoo* | MAI72 | S7_27480009 | 8.2E-04 | 2.7E-01 | 4.2 | -1.3 | A/G | 183/90 | * | LOC_Os07g46039 | expressed protein |
| *Xoo* | MAI72 | S7_27480033 | 8.2E-04 | 2.7E-01 | 4.2 | -1.3 | A/G | 183/90 | * | LOC_Os07g46039 | expressed protein |
| *Xoo* | MAI72 | S7_27480041 | 8.2E-04 | 2.7E-01 | 4.2 | -1.3 | C/G | 183/90 | * | LOC_Os07g46039 | expressed protein |
| *Xoo* | MAI72 | S7_27590866 | 1.0E-03 | 3.0E-01 | 4.1 | -1.2 | C/T | 189/89 | * | LOC_Os07g46240 | choline transporter-related, putative, expressed |
| *Xoo* | MAI72 | S7_27619799 | 6.7E-04 | 2.7E-01 | 4.2 | -1.2 | A/G | 185/97 | * | LOC_Os07g46300 | expressed protein |
| *Xoo* | MAI130 | S7_28070395 | 2.4E-04 | 1.9E-01 | 5.9 | -2.1 | A/G | 171/78 | * | LOC_Os07g46970 | sex determination protein tasselseed-2, putative, expressed |
| *Xoo* | MAI72 | S7_28165250 | 8.6E-04 | 2.7E-01 | 4.3 | -1.3 | C/T | 201/69 | * | LOC_Os07g47100 | transporter, monovalent cation:proton antiporter-2 family, putative, expressed |
| *Xoo* | MAI72 | S7_28165258 | 8.6E-04 | 2.7E-01 | 4.3 | -1.3 | A/G | 201/69 | * | LOC_Os07g47100 | transporter, monovalent cation:proton antiporter-2 family, putative, expressed |
| *Xoo* | MAI72 | S7_28421931 | 8.2E-04 | 2.7E-01 | 4.3 | -1.4 | G/A | 211/64 | * | intergenic |  |
| *Xoo* | MAI72 | S7_28421964 | 8.2E-04 | 2.7E-01 | 4.3 | -1.4 | T/A | 211/64 | * | intergenic |  |
| *Xoo* | MAI72 | S7_28445131 | 5.5E-04 | 2.7E-01 | 4.4 | -1.4 | A/G | 216/62 | * | LOC_Os07g47560 | expressed protein |
| *Xoo* | MAI72 | S7_28829259 | 8.5E-04 | 2.7E-01 | 4.2 | -1.4 | A/C | 214/62 | * | LOC_Os07g48260 | WRKY47, expressed |
| *Xoo* | MAI72 | S7_28849458 | 6.3E-04 | 2.7E-01 | 4.6 | -1.4 | G/T | 214/60 | * | LOC_Os07g48280 | expressed protein |
| *Xoo* | MAI130 | S7_28904931 | 9.3E-04 | 4.1E-01 | 4.4 | -1.7 | A/T | 149/104 | * | LOC_Os07g48360 | helicase conserved C-terminal domain containing protein, expressed |
|  |  |  |  |  |  |  |  |  |  |  |  |

**Continuation Table S5.**

| **Pathovar** | **Strain** | **Marker** | **p-value** | **q-value** | **R^2^** | **Effect** | **Effect** | **AILs with Effect** | **Significance** | **Locus** | **Annotation** |
| --- | --- | --- | --- | --- | --- | --- | --- | --- | --- | --- | --- |
|  |  | **(Chr_Pos)** |  |  |  | **estimate** | **allele/Null** | **allele/Null** | **correspondence** |  |  |
| *Xoc* | BAI5 | S8_2690001 | 3.3E-04 | 8.9E-02 | 4.5 | 5.1 | A/C | 78/210 | * | intergenic |  |
| *Xoo* | MAI101 | S8_8259055 | 6.2E-04 | 1.7E-01 | 5.2 | -4.1 | C/A | 184/45 | * | LOC_Os08g13840 | WRKY25, expressed |
|  |  |  |  |  |  |  |  |  |  |  |  |
| *Xoo* | BAI3 | S9_12319855 | 9.7E-04 | 1.3E-01 | 3.5 | -4.5 | A/G | 268/35 | * | LOC_Os09g20460 | VQ domain containing protein, putative, expressed |
| *Xoo* | BAI3 | S9_19185317 | 3.7E-04 | 6.1E-02 | 4.4 | -3.1 | A/G | 193/102 | * | LOC_Os09g32140 | expressed protein |
| *Xoo* | MAI134 | S9_20394634 | 9.4E-04 | 9.1E-01 | 4.3 | -1.3 | C/G | 251/30 | * | intergenic |  |
| *Xoc* | MAI10 | S9_21084075 | 8.5E-04 | 5.8E-01 | 5.0 | -3.4 | C/T | 211/21 | * | LOC_Os09g36550 | armadillo/beta-catenin repeat family protein, putative, expressed |
|  |  |  |  |  |  |  |  |  |  |  |  |
| *Xoo* | MAI130 | S10_11404753 | 8.2E-04 | 3.8E-01 | 4.7 | 1.8 | T/C | 101/152 | * | LOC_Os10g22080 | expressed protein |
| *Xoo* | MAI72 | S10_19571781 | 7.1E-04 | 2.7E-01 | 4.3 | -1.4 | G/A | 228/61 | * | intergenic |  |
| *Xoo* | MAI72 | S10_19571798 | 4.5E-04 | 2.7E-01 | 4.6 | -1.4 | T/G | 228/62 | * | intergenic |  |
| *Xoo* | MAI72 | S10_19598933 | 8.9E-04 | 2.7E-01 | 4.1 | -1.4 | C/A | 227/59 | * | LOC_Os10g36626 | expressed protein |
| *Xoo* | MAI72 | S10_19599061 | 5.0E-04 | 2.7E-01 | 4.5 | -1.4 | C/G | 223/63 | * | LOC_Os10g36626 | expressed protein |
| *Xoo* | MAI72 | S10_19603128 | 1.5E-04 | 2.2E-01 | 5.5 | -1.6 | C/T | 220/59 | * | intergenic |  |
| *Xoo* | MAI72 | S10_19621750 | 4.5E-04 | 2.7E-01 | 4.6 | -1.4 | G/C | 228/62 | * | LOC_Os10g36690 | dehydration response related protein, putative, expressed |
| *Xoo* | MAI72 | S10_19676741 | 4.3E-05 | 1.5E-01 | 6.3 | -1.6 | G/T | 228/62 | * | LOC_Os10g36750 | mitochondrial transcription termination factor-related, putative, expressed |
| *Xoo* | MAI72 | S10_19676742 | 4.3E-05 | 1.5E-01 | 6.3 | -1.6 | C/T | 228/62 | * | LOC_Os10g36750 | mitochondrial transcription termination factor-related, putative, expressed |
| *Xoo* | MAI72 | S10_19677331 | 7.3E-05 | 1.8E-01 | 5.9 | -1.6 | G/T | 229/61 | * | LOC_Os10g36750 | mitochondrial transcription termination factor-related, putative, expressed |
| *Xoo* | MAI72 | S10_19690604 | 3.0E-05 | 1.5E-01 | 7.2 | -1.8 | C/T | 196/56 | * | LOC_Os10g36780 | OsProCP4 - Putative Lysosomal Pro-x Carboxypeptidase homologue, expressed |
| *Xoo* | MAI72 | S10_19754550 | 5.1E-05 | 1.5E-01 | 6.8 | -1.7 | G/A | 213/55 | * | LOC_Os10g36870 | src homology-3 domain protein 3, putative, expressed |
| *Xoo* | MAI72 | S10_19827002 | 1.2E-04 | 1.9E-01 | 6.0 | -1.7 | A/G | 206/54 | * | LOC_Os10g37020 | transposon protein, putative, unclassified, expressed |
| *Xoo* | MAI72 | S10_19847581 | 2.8E-04 | 2.7E-01 | 4.9 | -1.4 | G/T | 227/63 | * | LOC_Os10g37070 | cytochrome P450, putative, expressed |
| *Xoo* | MAI93 | S10_20158998 | 8.8E-04 | 1.0E+00 | 4.2 | -3.8 | G/A | 185/86 | * | LOC_Os10g37660 | trehalase precursor, putative, expressed |
| *Xoo* | MAI93 | S10_20275330 | 7.5E-04 | 1.0E+00 | 4.3 | -3.8 | C/T | 192/84 | * | intergenic |  |
|  |  |  |  |  |  |  |  |  |  |  |  |
| *Xoc* | MAI123 | S11_3907887 | 7.9E-04 | 6.3E-01 | 4.7 | -1.9 | T/C | 149/70 | * | LOC_Os11g07650 | retrotransposon protein, putative, unclassified, expressed |
| *Xoo* | CFBP1951 | S11_4854344 | 3.4E-04 | 2.3E-01 | 5.3 | -1.5 | C/T | 221/24 | * | intergenic |  |

**Continuation Table S5.**

| **Pathovar** | **Strain** | **Marker** | **p-value** | **q-value** | **R^2^** | **Effect** | **Effect** | **AILs with Effect** | **Significance** | **Locus** | **Annotation** |
| --- | --- | --- | --- | --- | --- | --- | --- | --- | --- | --- | --- |
|  |  | **(Chr_Pos)** |  |  |  | **estimate** | **allele/Null** | **allele/Null** | **correspondence** |  |  |
| *Xoo* | MAI136 | S11_6923297 | 6.3E-05 | 6.3E-02 | 7.2 | -3.0 | T/C | 186/65 | * | intergenic |  |
| *Xoo* | MAI136 | S11_6940850 | 6.2E-04 | 2.6E-01 | 5.1 | -2.8 | G/A | 188/52 | * | LOC_Os11g12420 | serpin domain containing protein, putative, expressed |
| *Xoo* | BAI3 | **S11_6970503** | 7.8E-04 | 1.0E-01 | 3.9 | -3.0 | T/C | 163/134 | * | LOC_Os11g12470 | expressed protein |
| *Xoo* | MAI70 | **S11_6970503** | 3.6E-05 | 4.3E-02 | 6.3 | -3.4 | T/C | 150/133 | *** | LOC_Os11g12470 | expressed protein |
| *Xoo* | MAI70 | **S11_6970755** | 3.5E-04 | 2.5E-01 | 5.3 | -3.5 | C/T | 189/67 | * | LOC_Os11g12470 | expressed protein |
| *Xoo* | MAI130 | **S11_6970755** | 6.0E-04 | 3.3E-01 | 4.8 | -2.1 | C/T | 181/62 | * | LOC_Os11g12470 | expressed protein |
| *Xoo* | MAI136 | **S11_6970755** | 8.1E-05 | 6.9E-02 | 8.4 | -3.2 | C/T | 170/56 | * | LOC_Os11g12470 | expressed protein |
| *Xoo* | MAI70 | **S11_7102305** | 1.7E-04 | 1.7E-01 | 5.2 | 3.2 | C/A | 169/104 | * | intergenic |  |
| *Xoo* | MAI101 | **S11_7102305** | 4.7E-04 | 1.5E-01 | 5.2 | 2.9 | C/A | 150/90 | * | intergenic |  |
| *Xoo* | MAI70 | S11_7104422 | 1.3E-04 | 1.4E-01 | 5.3 | 3.3 | G/A | 173/105 | * | LOC_Os11g12620 | receptor protein kinase CLAVATA1 precursor, putative, expressed |
| *Xoo* | MAI70 | **S11_7104857** | 1.6E-04 | 1.7E-01 | 5.1 | 3.2 | T/G | 173/110 | * | LOC_Os11g12620 | receptor protein kinase CLAVATA1 precursor, putative, expressed |
| *Xoo* | MAI101 | **S11_7104857** | 2.3E-04 | 1.0E-01 | 5.5 | 3.1 | T/G | 153/94 | * | LOC_Os11g12620 | receptor protein kinase CLAVATA1 precursor, putative, expressed |
| *Xoo* | MAI70 | S11_7107785 | 2.1E-04 | 1.7E-01 | 5.0 | 3.2 | T/G | 171/104 | * | LOC_Os11g12620 | receptor protein kinase CLAVATA1 precursor, putative, expressed |
| *Xoo* | MAI72 | S11_9150528 | 5.6E-04 | 2.7E-01 | 4.4 | -1.2 | A/C | 133/138 | * | LOC_Os11g16530 | mal, putative, expressed |
| *Xoo* | MAI72 | S11_9456372 | 4.1E-04 | 2.7E-01 | 4.7 | -1.3 | T/C | 164/108 | * | LOC_Os11g17060 | expressed protein |
| *Xoo* | MAI72 | S11_9456382 | 4.1E-04 | 2.7E-01 | 4.7 | -1.3 | G/A | 164/108 | * | LOC_Os11g17060 | expressed protein |
| *Xoo* | MAI72 | S11_9465629 | 8.0E-04 | 2.7E-01 | 4.2 | -1.2 | A/G | 160/107 | * | LOC_Os11g17070 | expressed protein |
| *Xoo* | MAI72 | S11_9465651 | 6.4E-04 | 2.7E-01 | 4.3 | -1.2 | G/A | 160/108 | * | LOC_Os11g17070 | expressed protein |
| *Xoo* | MAI72 | S11_9465660 | 6.4E-04 | 2.7E-01 | 4.3 | -1.2 | G/A | 160/108 | * | LOC_Os11g17070 | expressed protein |
| *Xoo* | MAI72 | S11_16864795 | 7.7E-04 | 2.7E-01 | 4.4 | -1.2 | C/G | 127/124 | * | intergenic |  |
| *Xoo* | MAI72 | S11_17605358 | 4.9E-05 | 1.5E-01 | 6.8 | -1.6 | C/T | 172/88 | * | LOC_Os11g30320 | expressed protein |
| *Xoc* | MAI67 | S11_18071192 | 1.4E-04 | 1.6E-01 | 5.6 | -1.4 | A/G | 166/88 | * | LOC_Os11g31060 | IQ calmodulin-binding and BAG domain containing protein, putative, expressed |
| *Xoo* | MAI133 | S11_24397797 | 4.7E-04 | 3.4E-01 | 5.2 | 1.8 | C/T | 216/41 | * | LOC_Os11g40790 | expressed protein |
| *Xoo* | MAI70 | **S11_25082818** | 3.2E-04 | 2.4E-01 | 4.9 | -6.5 | T/C | 255/15 | * | intergenic |  |
| *Xoo* | MAI133 | **S11_25082818** | 1.9E-04 | 3.4E-01 | 5.9 | -3.3 | T/C | 222/12 | * | intergenic |  |
| *Xoo* | BAI3 | S11_25522745 | 2.5E-04 | 4.3E-02 | 4.3 | -6.7 | C/T | 302/17 | ** | LOC_Os11g42410 | expressed protein |
| *Xoo* | BAI3 | S11_25522746 | 2.5E-04 | 4.3E-02 | 4.3 | -6.7 | C/G | 302/17 | ** | LOC_Os11g42410 | expressed protein |
| *Xoo* | BAI3 | S11_25522749 | 2.5E-04 | 4.3E-02 | 4.3 | -6.7 | G/C | 302/17 | ** | LOC_Os11g42410 | expressed protein |

**Continuation Table S5.**

| **Pathovar** | **Strain** | **Marker** | **p-value** | **q-value** | **R^2^** | **Effect** | **Effect** | **AILs with Effect** | **Significance** | **Locus** | **Annotation** |
| --- | --- | --- | --- | --- | --- | --- | --- | --- | --- | --- | --- |
|  |  | **(Chr_Pos)** |  |  |  | **estimate** | **allele/Null** | **allele/Null** | **correspondence** |  |  |
| *Xoo* | BAI3 | S11_25522752 | 2.5E-04 | 4.3E-02 | 4.3 | -6.7 | C/G | 302/17 | ** | LOC_Os11g42410 | expressed protein |
| *Xoo* | BAI3 | S11_25522753 | 2.5E-04 | 4.3E-02 | 4.3 | -6.7 | C/A | 302/17 | ** | LOC_Os11g42410 | expressed protein |
| *Xoo* | BAI3 | S11_25522755 | 2.1E-04 | 4.0E-02 | 4.5 | -6.8 | G/C | 299/17 | ** | LOC_Os11g42410 | expressed protein |
| *Xoo* | BAI3 | S11_25591134 | 2.6E-04 | 4.4E-02 | 4.4 | -6.3 | G/T | 290/19 | ** | LOC_Os11g42490 | retrotransposon protein, putative, unclassified, expressed |
| *Xoc* | BLS256 | S11_26879946 | 8.2E-04 | 1.7E-01 | 4.7 | -11.2 | C/T | 237/20 | * | LOC_Os11g44430 | protein kinase, putative, expressed |
| *Xoc* | BLS256 | **S11_26950888** | 3.6E-05 | 4.2E-02 | 7.3 | -12.3 | A/G | 227/27 | *** | LOC_Os11g44570 | expressed protein |
| *Xoo* | BAI3 | **S11_26950888** | 2.5E-04 | 4.3E-02 | 4.6 | -5.4 | A/G | 277/33 | ** | LOC_Os11g44570 | expressed protein |
| *Xoo* | MAI101 | **S11_26950888** | 6.0E-04 | 1.7E-01 | 5.0 | -4.6 | A/G | 229/26 | * | LOC_Os11g44570 | expressed protein |
| *Xoo* | MAI136 | **S11_26950888** | 7.5E-04 | 2.9E-01 | 5.0 | -3.7 | A/G | 233/27 | * | LOC_Os11g44570 | expressed protein |
| *Xoc* | BLS256 | **S11_27033636** | 2.0E-05 | 3.6E-02 | 7.8 | -12.9 | A/G | 217/27 | *** | LOC_Os11g44700 | calmodulin binding protein, putative, expressed |
| *Xoo* | BAI3 | **S11_27033636** | 1.9E-04 | 3.7E-02 | 5.0 | -5.6 | A/G | 263/33 | ** | LOC_Os11g44700 | calmodulin binding protein, putative, expressed |
| *Xoo* | MAI145 | **S11_27033636** | 9.6E-04 | 2.9E-01 | 4.3 | -3.5 | A/G | 232/29 | * | LOC_Os11g44700 | calmodulin binding protein, putative, expressed |
| *Xoc* | BLS256 | **S11_27033687** | 1.7E-05 | 3.6E-02 | 7.9 | -12.8 | A/G | 226/27 | *** | LOC_Os11g44700 | calmodulin binding protein, putative, expressed |
| *Xoo* | BAI3 | **S11_27033687** | 7.3E-05 | 1.8E-02 | 5.4 | -5.8 | A/G | 272/34 | *** | LOC_Os11g44700 | calmodulin binding protein, putative, expressed |
| *Xoo* | MAI101 | **S11_27033687** | 3.9E-04 | 1.4E-01 | 5.5 | -4.6 | A/G | 228/28 | * | LOC_Os11g44700 | calmodulin binding protein, putative, expressed |
| *Xoc* | BLS256 | **S11_27181429** | 1.3E-05 | 3.6E-02 | 7.6 | -12.6 | C/G | 226/30 | *** | LOC_Os11g44910 | DEAD-box ATP-dependent RNA helicase, putative, expressed |
| *Xoo* | BAI3 | **S11_27181429** | 9.4E-06 | 4.1E-03 | 6.6 | -6.4 | C/G | 275/37 | *** | LOC_Os11g44910 | DEAD-box ATP-dependent RNA helicase, putative, expressed |
| *Xoo* | MAI101 | **S11_27181429** | 3.8E-05 | 3.1E-02 | 7.0 | -5.3 | C/G | 228/30 | *** | LOC_Os11g44910 | DEAD-box ATP-dependent RNA helicase, putative, expressed |
| *Xoo* | MAI136 | **S11_27181429** | 4.9E-05 | 6.0E-02 | 6.5 | -4.2 | C/G | 232/31 | * | LOC_Os11g44910 | DEAD-box ATP-dependent RNA helicase, putative, expressed |
| *Xoo* | MAI145 | **S11_27181429** | 2.0E-04 | 1.1E-01 | 5.2 | -3.8 | C/G | 242/32 | * | LOC_Os11g44910 | DEAD-box ATP-dependent RNA helicase, putative, expressed |
| *Xoc* | BLS256 | **S11_27181436** | 8.5E-05 | 6.7E-02 | 6.1 | -11.2 | A/G | 225/31 | * | LOC_Os11g44910 | DEAD-box ATP-dependent RNA helicase, putative, expressed |
| *Xoo* | BAI3 | **S11_27181436** | 1.6E-06 | 1.0E-03 | 7.9 | -6.8 | A/G | 272/38 | *** | LOC_Os11g44910 | DEAD-box ATP-dependent RNA helicase, putative, expressed |
| *Xoo* | MAI101 | **S11_27181436** | 2.0E-05 | 2.1E-02 | 7.7 | -5.4 | A/G | 227/31 | *** | LOC_Os11g44910 | DEAD-box ATP-dependent RNA helicase, putative, expressed |
| *Xoo* | MAI136 | **S11_27181436** | 1.7E-05 | 4.7E-02 | 7.2 | -4.4 | A/G | 231/32 | *** | LOC_Os11g44910 | DEAD-box ATP-dependent RNA helicase, putative, expressed |
| *Xoo* | MAI145 | **S11_27181436** | 1.5E-05 | 1.6E-02 | 7.0 | -4.4 | A/G | 241/33 | *** | LOC_Os11g44910 | DEAD-box ATP-dependent RNA helicase, putative, expressed |
| *Xoo* | BAI3 | S11_27183806 | 5.2E-05 | 1.4E-02 | 5.8 | -5.8 | A/G | 250/37 | *** | LOC_Os11g44910 | DEAD-box ATP-dependent RNA helicase, putative, expressed |

**Continuation Table S5.**

| **Pathovar** | **Strain** | **Marker** | **p-value** | **q-value** | **R^2^** | **Effect** | **Effect** | **AILs with Effect** | **Significance** | **Locus** | **Annotation** |
| --- | --- | --- | --- | --- | --- | --- | --- | --- | --- | --- | --- |
|  |  | **(Chr_Pos)** |  |  |  | **estimate** | **allele/Null** | **allele/Null** | **correspondence** |  |  |
| *Xoo* | BAI3 | **S11_27192125** | 7.7E-05 | 1.9E-02 | 5.9 | -5.8 | G/A | 260/34 | *** | LOC_Os11g44920 | expressed protein |
| *Xoo* | MAI133 | **S11_27192125** | 7.7E-04 | 3.4E-01 | 4.9 | -2.0 | G/A | 213/29 | * | LOC_Os11g44920 | expressed protein |
| *Xoo* | MAI136 | **S11_27192125** | 5.0E-04 | 2.4E-01 | 4.9 | -3.7 | G/A | 222/29 | * | LOC_Os11g44920 | expressed protein |
| *Xoo* | MAI145 | **S11_27192125** | 4.1E-04 | 2.0E-01 | 4.8 | -3.7 | G/A | 232/30 | * | LOC_Os11g44920 | expressed protein |
| *Xoc* | BLS256 | **S11_27197214** | 2.2E-04 | 8.8E-02 | 6.3 | -10.9 | T/C | 192/29 | * | intergenic |  |
| *Xoo* | BAI3 | **S11_27197214** | 1.6E-05 | 5.5E-03 | 7.0 | -6.4 | T/C | 236/36 | *** | intergenic |  |
| *Xoo* | MAI101 | **S11_27197214** | 4.4E-05 | 3.2E-02 | 7.4 | -5.3 | T/C | 194/29 | *** | intergenic |  |
| *Xoo* | MAI136 | **S11_27197214** | 4.1E-04 | 2.2E-01 | 5.6 | -3.7 | T/C | 197/30 | * | intergenic |  |
| *Xoo* | MAI145 | **S11_27197214** | 4.5E-04 | 2.0E-01 | 5.3 | -3.7 | T/C | 205/31 | * | intergenic |  |
| *Xoc* | BLS256 | **S11_27197220** | 2.2E-04 | 8.8E-02 | 6.3 | -10.9 | T/C | 192/29 | * | intergenic |  |
| *Xoo* | BAI3 | **S11_27197220** | 1.6E-05 | 5.5E-03 | 7.0 | -6.4 | T/C | 236/36 | *** | intergenic |  |
| *Xoo* | MAI101 | **S11_27197220** | 4.4E-05 | 3.2E-02 | 7.4 | -5.3 | T/C | 194/29 | *** | intergenic |  |
| *Xoo* | MAI136 | **S11_27197220** | 4.1E-04 | 2.2E-01 | 5.6 | -3.7 | T/C | 197/30 | * | intergenic |  |
| *Xoo* | MAI145 | **S11_27197220** | 4.5E-04 | 2.0E-01 | 5.3 | -3.7 | T/C | 205/31 | * | intergenic |  |
| *Xoc* | BLS256 | **S11_27205864** | 1.6E-04 | 8.6E-02 | 6.0 | -10.8 | T/G | 227/30 | * | LOC_Os11g44950 | glycosyl hydrolase family 3 protein, putative, expressed |
| *Xoo* | BAI3 | **S11_27205864** | 1.6E-05 | 5.5E-03 | 6.3 | -6.1 | T/G | 275/38 | *** | LOC_Os11g44950 | glycosyl hydrolase family 3 protein, putative, expressed |
| *Xoo* | MAI101 | **S11_27205864** | 3.4E-04 | 1.4E-01 | 5.4 | -4.5 | T/G | 228/31 | * | LOC_Os11g44950 | glycosyl hydrolase family 3 protein, putative, expressed |
| *Xoo* | MAI136 | **S11_27205864** | 2.5E-04 | 1.5E-01 | 5.6 | -3.7 | T/G | 232/32 | * | LOC_Os11g44950 | glycosyl hydrolase family 3 protein, putative, expressed |
| *Xoc* | BLS256 | **S11_27393912** | 1.7E-04 | 8.6E-02 | 5.9 | -10.6 | T/C | 226/31 | * | intergenic |  |
| *Xoo* | BAI3 | **S11_27393912** | 8.2E-06 | 3.7E-03 | 6.8 | -6.2 | T/C | 274/39 | *** | intergenic |  |
| *Xoo* | MAI101 | **S11_27393912** | 2.9E-04 | 1.2E-01 | 5.5 | -4.4 | T/C | 227/32 | * | intergenic |  |
| *Xoo* | MAI136 | **S11_27393912** | 6.2E-04 | 2.6E-01 | 4.8 | -3.4 | T/C | 231/33 | * | intergenic |  |
| *Xoo* | BAI3 | S11_27412501 | 1.6E-04 | 3.5E-02 | 5.7 | -5.7 | G/C | 236/31 | ** | LOC_Os11g45295 | retrotransposon protein, putative, unclassified, expressed |
| *Xoo* | BAI3 | S11_27412530 | 1.6E-04 | 3.5E-02 | 5.7 | -5.7 | T/C | 236/31 | ** | LOC_Os11g45295 | retrotransposon protein, putative, unclassified, expressed |
| *Xoc* | BLS256 | S11_27442768 | 2.3E-04 | 9.0E-02 | 6.1 | -12.6 | C/A | 204/20 | * | intergenic |  |

**Continuation Table S5.**

| **Pathovar** | **Strain** | **Marker** | **p-value** | **q-value** | **R^2^** | **Effect** | **Effect** | **AILs with Effect** | **Significance** | **Locus** | **Annotation** |
| --- | --- | --- | --- | --- | --- | --- | --- | --- | --- | --- | --- |
|  |  | **(Chr_Pos)** |  |  |  | **estimate** | **allele/Null** | **allele/Null** | **correspondence** |  |  |
| *Xoc* | BLS256 | **S11_27442782** | 9.7E-07 | 1.4E-02 | 11.1 | -14.4 | A/G | 195/29 | *** | intergenic |  |
| *Xoo* | BAI3 | **S11_27442782** | 1.0E-04 | 2.3E-02 | 6.1 | -5.7 | A/G | 238/35 | ** | intergenic |  |
| *Xoo* | MAI101 | **S11_27442782** | 5.9E-05 | 3.7E-02 | 8.6 | -5.3 | A/G | 193/27 | *** | intergenic |  |
| *Xoo* | MAI133 | **S11_27442782** | 6.7E-04 | 3.4E-01 | 5.4 | -2.0 | A/G | 194/29 | * | intergenic |  |
| *Xoo* | MAI145 | **S11_27442782** | 5.3E-04 | 2.1E-01 | 5.6 | -3.7 | A/G | 208/30 | * | intergenic |  |
| *Xoc* | BLS256 | **S11_27451641** | 8.2E-05 | 6.7E-02 | 6.6 | -11.7 | C/G | 221/28 | * | LOC_Os11g45380 | zinc finger family protein, putative, expressed |
| *Xoo* | BAI3 | **S11_27451641** | 9.6E-05 | 2.2E-02 | 5.4 | -5.8 | C/G | 268/34 | *** | LOC_Os11g45380 | zinc finger family protein, putative, expressed |
| *Xoo* | MAI101 | **S11_27451641** | 8.2E-05 | 4.9E-02 | 6.4 | -5.2 | C/G | 224/27 | *** | LOC_Os11g45380 | zinc finger family protein, putative, expressed |
| *Xoo* | MAI133 | **S11_27451641** | 3.8E-04 | 3.4E-01 | 5.2 | -2.1 | C/G | 220/28 | * | LOC_Os11g45380 | zinc finger family protein, putative, expressed |
| *Xoo* | MAI145 | **S11_27451641** | 5.6E-04 | 2.2E-01 | 4.6 | -3.7 | C/G | 237/29 | * | LOC_Os11g45380 | zinc finger family protein, putative, expressed |
| *Xoo* | BAI3 | S11_27464711 | 2.3E-04 | 4.3E-02 | 4.9 | -5.2 | T/C | 254/36 | ** | intergenic |  |
| *Xoc* | BLS256 | **S11_27464784** | 2.1E-04 | 8.8E-02 | 5.6 | -11.1 | T/C | 216/27 | * | intergenic |  |
| *Xoo* | BAI3 | **S11_27464784** | 3.2E-05 | 9.7E-03 | 6.3 | -6.1 | T/C | 257/34 | *** | intergenic |  |
| *Xoo* | MAI101 | **S11_27464784** | 1.4E-04 | 7.7E-02 | 6.2 | -5.0 | T/C | 218/27 | * | intergenic |  |
| *Xoo* | MAI136 | **S11_27464784** | 4.8E-04 | 2.4E-01 | 5.2 | -3.7 | T/C | 218/28 | * | intergenic |  |
| *Xoc* | BLS256 | **S11_27468322** | 1.8E-04 | 8.6E-02 | 6.0 | -10.7 | A/G | 222/30 | * | LOC_Os11g45390 | von Willebrand factor type A domain containing protein, putative, expressed |
| *Xoo* | BAI3 | **S11_27468322** | 5.2E-05 | 1.4E-02 | 5.8 | -5.8 | A/G | 269/37 | *** | LOC_Os11g45390 | von Willebrand factor type A domain containing protein, putative, expressed |
| *Xoo* | MAI101 | **S11_27468322** | 8.9E-05 | 5.2E-02 | 6.3 | -4.9 | A/G | 224/31 | * | LOC_Os11g45390 | von Willebrand factor type A domain containing protein, putative, expressed |
| *Xoo* | MAI145 | **S11_27468322** | 8.3E-04 | 2.7E-01 | 4.3 | -3.4 | A/G | 240/32 | * | LOC_Os11g45390 | von Willebrand factor type A domain containing protein, putative, expressed |
| *Xoc* | BLS256 | **S11_27483001** | 8.9E-05 | 6.7E-02 | 6.4 | -11.4 | A/G | 227/29 | * | intergenic |  |
| *Xoo* | BAI3 | **S11_27483001** | 3.6E-05 | 1.1E-02 | 5.8 | -6.0 | A/G | 276/36 | *** | intergenic |  |
| *Xoo* | MAI101 | **S11_27483001** | 1.7E-04 | 8.7E-02 | 5.8 | -4.9 | A/G | 229/29 | * | intergenic |  |
| *Xoo* | MAI136 | **S11_27483001** | 5.4E-04 | 2.5E-01 | 5.1 | -3.6 | A/G | 233/30 | * | intergenic |  |
| *Xoo* | MAI145 | **S11_27483001** | 8.6E-04 | 2.7E-01 | 4.4 | -3.5 | A/G | 243/31 | * | intergenic |  |
| *Xoc* | BLS256 | **S11_27496126** | 2.0E-04 | 8.8E-02 | 5.5 | -10.8 | G/A | 225/29 | * | LOC_Os11g45410 | expressed protein |
| *Xoo* | BAI3 | **S11_27496126** | 1.3E-04 | 2.9E-02 | 5.1 | -5.4 | G/A | 270/37 | ** | LOC_Os11g45410 | expressed protein |

**Continuation Table S5.**

| **Pathovar** | **Strain** | **Marker** | **p-value** | **q-value** | **R^2^** | **Effect** | **Effect** | **AILs with Effect** | **Significance** | **Locus** | **Annotation** |
| --- | --- | --- | --- | --- | --- | --- | --- | --- | --- | --- | --- |
|  |  | **(Chr_Pos)** |  |  |  | **estimate** | **allele/Null** | **allele/Null** | **correspondence** |  |  |
| *Xoc* | BLS256 | **S11_27496991** | 1.3E-04 | 8.1E-02 | 6.2 | -11.2 | G/A | 226/29 | * | LOC_Os11g45410 | expressed protein |
| *Xoo* | BAI3 | **S11_27496991** | 3.4E-05 | 1.0E-02 | 5.9 | -6.1 | G/A | 275/36 | *** | LOC_Os11g45410 | expressed protein |
| *Xoo* | MAI101 | **S11_27496991** | 1.4E-04 | 7.7E-02 | 6.1 | -4.9 | G/A | 228/29 | * | LOC_Os11g45410 | expressed protein |
| *Xoo* | MAI136 | **S11_27496991** | 6.4E-04 | 2.6E-01 | 4.8 | -3.6 | G/A | 232/30 | * | LOC_Os11g45410 | expressed protein |
| *Xoo* | MAI145 | **S11_27496991** | 8.0E-04 | 2.6E-01 | 4.4 | -3.5 | G/A | 242/31 | * | LOC_Os11g45410 | expressed protein |
| *Xoc* | BLS256 | S11_27574157 | 6.9E-04 | 1.7E-01 | 4.7 | -10.7 | A/T | 232/23 | * | LOC_Os11g45540 | TKL_IRAK_DUF26-lh.11 - DUF26 kinases have homology to DUF26 containing loci, expressed |
| *Xoc* | BLS256 | **S11_27603799** | 8.5E-04 | 1.7E-01 | 4.6 | -9.1 | A/C | 222/32 | * | LOC_Os11g45620 | rust-resistance protein Lr21, putative, expressed |
| *Xoo* | BAI3 | **S11_27603799** | 6.7E-05 | 1.7E-02 | 5.5 | -5.4 | A/C | 270/40 | *** | LOC_Os11g45620 | rust-resistance protein Lr21, putative, expressed |
| *Xoo* | MAI136 | **S11_27603799** | 7.9E-04 | 3.0E-01 | 4.8 | -3.3 | A/C | 227/34 | * | LOC_Os11g45620 | rust-resistance protein Lr21, putative, expressed |
| *Xoo* | MAI145 | **S11_27603799** | 5.8E-04 | 2.2E-01 | 4.6 | -3.4 | A/C | 238/35 | * | LOC_Os11g45620 | rust-resistance protein Lr21, putative, expressed |
| *Xoc* | BLS256 | **S11_27672705** | 5.3E-04 | 1.4E-01 | 5.3 | -9.8 | C/T | 208/32 | * | LOC_Os11g45740 | MYB family transcription factor, putative, expressed |
| *Xoo* | BAI3 | **S11_27672705** | 3.8E-06 | 1.8E-03 | 7.9 | -6.6 | C/T | 253/40 | *** | LOC_Os11g45740 | MYB family transcription factor, putative, expressed |
| *Xoo* | MAI101 | **S11_27672705** | 2.0E-05 | 2.1E-02 | 8.1 | -5.3 | C/T | 208/32 | *** | LOC_Os11g45740 | MYB family transcription factor, putative, expressed |
| *Xoo* | MAI133 | **S11_27672705** | 6.2E-04 | 3.4E-01 | 5.1 | -1.9 | C/T | 206/33 | * | LOC_Os11g45740 | MYB family transcription factor, putative, expressed |
| *Xoo* | MAI145 | **S11_27672705** | 1.6E-05 | 1.6E-02 | 7.6 | -4.3 | C/T | 221/35 | *** | LOC_Os11g45740 | MYB family transcription factor, putative, expressed |
| *Xoc* | BLS256 | **S11_27672709** | 5.3E-04 | 1.4E-01 | 5.3 | -9.8 | A/G | 208/32 | * | LOC_Os11g45740 | MYB family transcription factor, putative, expressed |
| *Xoo* | BAI3 | **S11_27672709** | 3.8E-06 | 1.8E-03 | 7.9 | -6.6 | A/G | 253/40 | *** | LOC_Os11g45740 | MYB family transcription factor, putative, expressed |
| *Xoo* | MAI101 | **S11_27672709** | 2.0E-05 | 2.1E-02 | 8.1 | -5.3 | A/G | 208/32 | *** | LOC_Os11g45740 | MYB family transcription factor, putative, expressed |
| *Xoo* | MAI133 | **S11_27672709** | 6.2E-04 | 3.4E-01 | 5.1 | -1.9 | A/G | 206/33 | * | LOC_Os11g45740 | MYB family transcription factor, putative, expressed |
| *Xoo* | MAI145 | **S11_27672709** | 1.6E-05 | 1.6E-02 | 7.6 | -4.3 | A/G | 221/35 | *** | LOC_Os11g45740 | MYB family transcription factor, putative, expressed |
| *Xoc* | BLS256 | **S11_27672719** | 5.3E-04 | 1.4E-01 | 5.3 | -9.8 | T/C | 208/32 | * | LOC_Os11g45740 | MYB family transcription factor, putative, expressed |
| *Xoo* | BAI3 | **S11_27672719** | 3.8E-06 | 1.8E-03 | 7.9 | -6.6 | T/C | 253/40 | *** | LOC_Os11g45740 | MYB family transcription factor, putative, expressed |
| *Xoo* | MAI101 | **S11_27672719** | 2.0E-05 | 2.1E-02 | 8.1 | -5.3 | T/C | 208/32 | *** | LOC_Os11g45740 | MYB family transcription factor, putative, expressed |
| *Xoo* | MAI133 | **S11_27672719** | 6.2E-04 | 3.4E-01 | 5.1 | -1.9 | T/C | 206/33 | * | LOC_Os11g45740 | MYB family transcription factor, putative, expressed |
| *Xoo* | MAI145 | **S11_27672719** | 1.6E-05 | 1.6E-02 | 7.6 | -4.3 | T/C | 221/35 | *** | LOC_Os11g45740 | MYB family transcription factor, putative, expressed |

**Continuation Table S5.**

| **Pathovar** | **Strain** | **Marker** | **p-value** | **q-value** | **R^2^** | **Effect** | **Effect** | **AILs with Effect** | **Significance** | **Locus** | **Annotation** |
| --- | --- | --- | --- | --- | --- | --- | --- | --- | --- | --- | --- |
|  |  | **(Chr_Pos)** |  |  |  | **estimate** | **allele/Null** | **allele/Null** | **correspondence** |  |  |
| *Xoo* | BAI3 | **S11_28483934** | 1.0E-05 | 4.2E-03 | 7.0 | -8.2 | A/C | 264/16 | *** | LOC_Os11g47350 | beta-D-xylosidase, putative, expressed |
| *Xoo* | MAI101 | **S11_28483934** | 2.0E-04 | 9.4E-02 | 5.8 | -6.1 | A/C | 219/15 | * | LOC_Os11g47350 | beta-D-xylosidase, putative, expressed |
| *Xoo* | MAI133 | **S11_28483934** | 5.1E-04 | 3.4E-01 | 5.3 | -3.0 | A/C | 218/13 | * | LOC_Os11g47350 | beta-D-xylosidase, putative, expressed |
| *Xoo* | MAI136 | **S11_28483934** | 4.4E-05 | 6.0E-02 | 8.1 | -5.8 | A/C | 224/14 | * | LOC_Os11g47350 | beta-D-xylosidase, putative, expressed |
| *Xoo* | MAI145 | **S11_28483934** | 1.6E-05 | 1.6E-02 | 7.9 | -5.9 | A/C | 232/15 | *** | LOC_Os11g47350 | beta-D-xylosidase, putative, expressed |
| *Xoo* | BAI3 | **S11_28483987** | 1.5E-06 | 9.9E-04 | 8.5 | -8.5 | C/T | 274/17 | *** | LOC_Os11g47350 | beta-D-xylosidase, putative, expressed |
| *Xoo* | MAI101 | **S11_28483987** | 4.0E-04 | 1.4E-01 | 5.1 | -5.6 | C/T | 229/16 | * | LOC_Os11g47350 | beta-D-xylosidase, putative, expressed |
| *Xoo* | MAI133 | **S11_28483987** | 8.8E-04 | 3.5E-01 | 4.7 | -2.7 | C/T | 230/14 | * | LOC_Os11g47350 | beta-D-xylosidase, putative, expressed |
| *Xoo* | MAI136 | **S11_28483987** | 1.1E-04 | 8.6E-02 | 6.2 | -5.3 | C/T | 236/15 | * | LOC_Os11g47350 | beta-D-xylosidase, putative, expressed |
| *Xoo* | MAI145 | **S11_28483987** | 6.2E-05 | 4.1E-02 | 6.2 | -5.3 | C/T | 243/16 | *** | LOC_Os11g47350 | beta-D-xylosidase, putative, expressed |
| *Xoc* | BLS256 | **S11_28652242** | 2.3E-04 | 9.0E-02 | 5.6 | -10.7 | T/C | 224/29 | * | intergenic |  |
| *Xoo* | BAI3 | **S11_28652242** | 3.4E-07 | 2.9E-04 | 9.1 | -7.5 | T/C | 272/36 | *** | intergenic |  |
| *Xoo* | MAI70 | **S11_28652242** | 5.5E-04 | 3.8E-01 | 4.2 | -4.6 | T/C | 260/32 | * | intergenic |  |
| *Xoo* | MAI101 | **S11_28652242** | 5.6E-06 | 2.1E-02 | 8.8 | -5.9 | T/C | 227/29 | *** | intergenic |  |
| *Xoo* | MAI133 | **S11_28652242** | 6.5E-05 | 3.4E-01 | 6.5 | -2.3 | T/C | 222/30 | * | intergenic |  |
| *Xoo* | MAI136 | **S11_28652242** | 5.0E-05 | 6.0E-02 | 7.0 | -4.3 | T/C | 230/30 | * | intergenic |  |
| *Xoo* | MAI145 | **S11_28652242** | 1.8E-06 | 8.4E-03 | 8.9 | -4.9 | T/C | 240/32 | *** | intergenic |  |
| *Xoc* | BLS256 | **S11_28652283** | 9.9E-04 | 1.9E-01 | 4.5 | -9.3 | A/C | 225/30 | * | intergenic |  |
| *Xoo* | BAI3 | **S11_28652283** | 8.9E-08 | 8.0E-05 | 9.9 | -7.6 | A/C | 274/37 | *** | intergenic |  |
| *Xoo* | MAI70 | **S11_28652283** | 2.1E-04 | 1.7E-01 | 4.7 | -4.8 | A/C | 261/33 | * | intergenic |  |
| *Xoo* | MAI101 | **S11_28652283** | 7.9E-06 | 2.1E-02 | 8.4 | -5.7 | A/C | 227/30 | *** | intergenic |  |
| *Xoo* | MAI133 | **S11_28652283** | 1.7E-04 | 3.4E-01 | 5.8 | -2.2 | A/C | 222/31 | * | intergenic |  |
| *Xoo* | MAI136 | **S11_28652283** | 1.1E-04 | 8.6E-02 | 6.3 | -4.0 | A/C | 232/31 | * | intergenic |  |
| *Xoo* | MAI145 | **S11_28652283** | 5.7E-07 | 8.3E-03 | 9.7 | -5.1 | A/C | 240/33 | *** | intergenic |  |
| *Xoo* | MAI145 | S11_28656770 | 1.5E-05 | 1.6E-02 | 7.7 | -5.8 | G/A | 238/16 | *** | intergenic |  |

**Continuation Table S5.**

| **Pathovar** | **Strain** | **Marker** | **p-value** | **q-value** | **R^2^** | **Effect** | **Effect** | **AILs with Effect** | **Significance** | **Locus** | **Annotation** |
| --- | --- | --- | --- | --- | --- | --- | --- | --- | --- | --- | --- |
|  |  | **(Chr_Pos)** |  |  |  | **estimate** | **allele/Null** | **allele/Null** | **correspondence** |  |  |
| *Xoc* | BLS256 | **S11_28697227** | 4.4E-04 | 1.3E-01 | 5.1 | -9.9 | A/G | 229/31 | * | intergenic |  |
| *Xoo* | BAI3 | **S11_28697227** | 8.0E-07 | 5.8E-04 | 8.3 | -7.1 | A/G | 277/38 | *** | intergenic |  |
| *Xoo* | MAI101 | **S11_28697227** | 1.2E-05 | 2.1E-02 | 8.0 | -5.5 | A/G | 230/31 | *** | intergenic |  |
| *Xoo* | MAI133 | **S11_28697227** | 4.4E-04 | 3.4E-01 | 4.9 | -2.0 | A/G | 227/32 | * | intergenic |  |
| *Xoo* | MAI136 | **S11_28697227** | 5.3E-04 | 2.5E-01 | 4.9 | -3.5 | A/G | 234/32 | * | intergenic |  |
| *Xoo* | MAI145 | **S11_28697227** | 4.9E-06 | 1.4E-02 | 8.0 | -4.6 | A/G | 243/34 | *** | intergenic |  |
| *Xoc* | BLS256 | **S11_28697250** | 3.0E-04 | 1.0E-01 | 5.4 | -10.5 | G/C | 228/29 | * | intergenic |  |
| *Xoo* | BAI3 | **S11_28697250** | 5.1E-07 | 4.1E-04 | 8.8 | -7.3 | G/C | 277/36 | *** | intergenic |  |
| *Xoo* | MAI101 | **S11_28697250** | 1.7E-05 | 2.1E-02 | 7.9 | -5.5 | G/C | 230/29 | *** | intergenic |  |
| *Xoo* | MAI133 | **S11_28697250** | 7.6E-04 | 3.4E-01 | 4.6 | -2.0 | G/C | 226/30 | * | intergenic |  |
| *Xoo* | MAI136 | **S11_28697250** | 1.5E-04 | 1.1E-01 | 5.9 | -4.0 | G/C | 234/30 | * | intergenic |  |
| *Xoo* | MAI145 | **S11_28697250** | 3.7E-06 | 1.4E-02 | 8.4 | -4.8 | G/C | 243/32 | *** | intergenic |  |
| *Xoc* | BLS256 | **S11_28775260** | 7.8E-04 | 1.7E-01 | 4.8 | -9.0 | T/C | 195/35 | * | LOC_Os11g47650 | trp repressor/replication initiator, putative, expressed |
| *Xoo* | BAI3 | **S11_28775260** | 1.9E-06 | 1.1E-03 | 8.3 | -6.5 | T/C | 241/42 | *** | LOC_Os11g47650 | trp repressor/replication initiator, putative, expressed |
| *Xoo* | MAI70 | **S11_28775260** | 6.0E-04 | 3.9E-01 | 5.0 | -4.2 | T/C | 229/38 | * | LOC_Os11g47650 | trp repressor/replication initiator, putative, expressed |
| *Xoo* | MAI101 | **S11_28775260** | 5.9E-06 | 2.1E-02 | 10.1 | -5.4 | T/C | 199/35 | *** | LOC_Os11g47650 | trp repressor/replication initiator, putative, expressed |
| *Xoo* | MAI133 | **S11_28775260** | 3.3E-04 | 3.4E-01 | 5.7 | -2.0 | T/C | 197/35 | * | LOC_Os11g47650 | trp repressor/replication initiator, putative, expressed |
| *Xoo* | MAI136 | **S11_28775260** | 7.0E-05 | 6.3E-02 | 8.5 | -3.9 | T/C | 200/36 | * | LOC_Os11g47650 | trp repressor/replication initiator, putative, expressed |
| *Xoo* | MAI145 | **S11_28775260** | 1.4E-06 | 8.4E-03 | 10.6 | -4.7 | T/C | 211/38 | *** | LOC_Os11g47650 | trp repressor/replication initiator, putative, expressed |
| *Xoo* | BAI3 | S11_28777668 | 8.2E-05 | 1.9E-02 | 6.1 | -7.3 | T/C | 250/16 | *** | LOC_Os11g47650 | trp repressor/replication initiator, putative, expressed |
| *Xoo* | BAI3 | S11_28784287 | 2.3E-04 | 4.3E-02 | 4.5 | -6.6 | C/T | 293/17 | ** | LOC_Os11g47690 | zinc finger, C3HC4 type domain containing protein, expressed |
| *Xoo* | BAI3 | S11_28784296 | 1.9E-04 | 3.7E-02 | 4.7 | -6.7 | G/A | 292/17 | ** | LOC_Os11g47690 | zinc finger, C3HC4 type domain containing protein, expressed |
| *Xoo* | BAI3 | **S11_28794135** | 7.0E-04 | 9.7E-02 | 3.7 | -5.9 | G/T | 302/17 | * | LOC_Os11g47730 | expressed protein |
| *Xoo* | MAI145 | **S11_28794135** | 4.5E-04 | 2.0E-01 | 4.4 | -4.6 | G/T | 265/16 | * | LOC_Os11g47730 | expressed protein |
| *Xoo* | BAI3 | **S11_28815283** | 1.9E-05 | 6.3E-03 | 6.8 | -8.2 | C/T | 278/16 | *** | LOC_Os11g47780 | pollen signalling protein with adenylyl cyclase activity, putative, expressed |
| *Xoo* | MAI136 | **S11_28815283** | 4.8E-04 | 2.4E-01 | 5.3 | -5.0 | C/T | 235/14 | * | LOC_Os11g47780 | pollen signalling protein with adenylyl cyclase activity, putative, expressed |
| *Xoo* | MAI145 | **S11_28815283** | 5.8E-04 | 2.2E-01 | 4.9 | -4.7 | C/T | 244/15 | * | LOC_Os11g47780 | pollen signalling protein with adenylyl cyclase activity, putative, expressed |

**Continuation Table S5.**

| **Pathovar** | **Strain** | **Marker** | **p-value** | **q-value** | **R^2^** | **Effect** | **Effect** | **AILs with Effect** | **Significance** | **Locus** | **Annotation** |
| --- | --- | --- | --- | --- | --- | --- | --- | --- | --- | --- | --- |
|  |  | **(Chr_Pos)** |  |  |  | **estimate** | **allele/Null** | **allele/Null** | **correspondence** |  |  |
| *Xoc* | BLS256 | **S11_28815394** | 8.6E-04 | 1.7E-01 | 5.0 | -13.5 | G/A | 223/12 | * | LOC_Os11g47780 | pollen signalling protein with adenylyl cyclase activity, putative, expressed |
| *Xoo* | BAI3 | **S11_28815394** | 1.1E-05 | 4.4E-03 | 7.3 | -8.1 | G/A | 269/17 | *** | LOC_Os11g47780 | pollen signalling protein with adenylyl cyclase activity, putative, expressed |
| *Xoo* | MAI101 | **S11_28815394** | 3.7E-04 | 1.4E-01 | 5.9 | -5.6 | G/A | 224/16 | * | LOC_Os11g47780 | pollen signalling protein with adenylyl cyclase activity, putative, expressed |
| *Xoo* | BAI3 | **S11_28852557** | 1.0E-05 | 4.3E-03 | 6.3 | -7.7 | T/G | 302/18 | *** | LOC_Os11g47830 | RNA recognition motif containing protein, expressed |
| *Xoo* | MAI101 | **S11_28852557** | 8.4E-04 | 2.3E-01 | 4.3 | -5.1 | T/G | 249/17 | * | LOC_Os11g47830 | RNA recognition motif containing protein, expressed |
| *Xoo* | MAI145 | **S11_28852557** | 9.1E-04 | 2.8E-01 | 4.0 | -4.2 | T/G | 265/17 | * | LOC_Os11g47830 | RNA recognition motif containing protein, expressed |
| *Xoo* | MAI133 | S11_28865011 | 3.8E-04 | 3.4E-01 | 5.2 | -1.5 | A/C | 176/73 | * | LOC_Os11g47860 | expressed protein |
| *Xoc* | BLS256 | **S11_28865030** | 2.5E-04 | 9.0E-02 | 5.3 | -9.8 | C/G | 221/34 | * | LOC_Os11g47860 | expressed protein |
| *Xoo* | BAI3 | **S11_28865030** | 2.8E-06 | 1.5E-03 | 7.5 | -6.4 | C/G | 269/41 | *** | LOC_Os11g47860 | expressed protein |
| *Xoo* | MAI101 | **S11_28865030** | 1.7E-04 | 8.7E-02 | 5.9 | -4.4 | C/G | 222/35 | * | LOC_Os11g47860 | expressed protein |
| *Xoo* | MAI136 | **S11_28865030** | 2.2E-04 | 1.4E-01 | 5.3 | -3.5 | C/G | 227/36 | * | LOC_Os11g47860 | expressed protein |
| *Xoo* | MAI145 | **S11_28865030** | 9.4E-06 | 1.6E-02 | 7.4 | -4.3 | C/G | 236/36 | *** | LOC_Os11g47860 | expressed protein |
| *Xoo* | BAI3 | S11_28870400 | 1.7E-04 | 3.6E-02 | 5.4 | -6.5 | C/T | 246/18 | ** | LOC_Os11g47870 | GRAS family transcription factor domain containing protein, expressed |
| *Xoo* | BAI3 | **S11_28870403** | 2.4E-04 | 4.3E-02 | 5.3 | -4.8 | T/C | 219/41 | ** | LOC_Os11g47870 | GRAS family transcription factor domain containing protein, expressed |
| *Xoo* | MAI145 | **S11_28870403** | 6.4E-04 | 2.3E-01 | 5.2 | -3.3 | T/C | 190/37 | * | LOC_Os11g47870 | GRAS family transcription factor domain containing protein, expressed |
| *Xoo* | BAI3 | **S11_28870482** | 6.6E-07 | 5.0E-04 | 9.7 | -7.0 | A/G | 225/39 | *** | LOC_Os11g47870 | GRAS family transcription factor domain containing protein, expressed |
| *Xoo* | MAI101 | **S11_28870482** | 4.1E-04 | 1.4E-01 | 6.9 | -4.4 | A/G | 182/32 | * | LOC_Os11g47870 | GRAS family transcription factor domain containing protein, expressed |
| *Xoo* | MAI145 | **S11_28870482** | 3.9E-05 | 3.3E-02 | 8.2 | -4.1 | A/G | 194/35 | *** | LOC_Os11g47870 | GRAS family transcription factor domain containing protein, expressed |
| *Xoo* | BAI3 | **S11_28870897** | 5.1E-05 | 1.4E-02 | 5.8 | -5.4 | A/C | 254/41 | *** | LOC_Os11g47870 | GRAS family transcription factor domain containing protein, expressed |
| *Xoo* | MAI145 | **S11_28870897** | 3.2E-04 | 1.6E-01 | 5.0 | -3.5 | A/C | 222/36 | * | LOC_Os11g47870 | GRAS family transcription factor domain containing protein, expressed |
| *Xoc* | BLS256 | **S11_28870941** | 2.5E-04 | 9.0E-02 | 5.7 | -10.1 | G/T | 206/33 | * | LOC_Os11g47870 | GRAS family transcription factor domain containing protein, expressed |
| *Xoo* | BAI3 | **S11_28870941** | 2.7E-05 | 8.5E-03 | 6.3 | -5.8 | G/T | 253/41 | *** | LOC_Os11g47870 | GRAS family transcription factor domain containing protein, expressed |
| *Xoo* | MAI101 | **S11_28870941** | 4.8E-04 | 1.5E-01 | 5.2 | -4.2 | G/T | 210/34 | * | LOC_Os11g47870 | GRAS family transcription factor domain containing protein, expressed |
| *Xoo* | MAI136 | **S11_28870941** | 5.7E-05 | 6.3E-02 | 7.9 | -4.0 | G/T | 213/34 | * | LOC_Os11g47870 | GRAS family transcription factor domain containing protein, expressed |
| *Xoo* | MAI145 | **S11_28870941** | 1.4E-05 | 1.6E-02 | 7.4 | -4.3 | G/T | 222/36 | *** | LOC_Os11g47870 | GRAS family transcription factor domain containing protein, expressed |
| *Xoo* | BAI3 | **S11_28895438** | 2.5E-04 | 4.3E-02 | 4.3 | -6.2 | C/A | 299/18 | ** | LOC_Os11g47910 | SCARECROW, putative, expressed |
| *Xoo* | MAI145 | **S11_28895438** | 7.7E-04 | 2.6E-01 | 4.1 | -4.3 | C/A | 262/17 | * | LOC_Os11g47910 | SCARECROW, putative, expressed |
| *Xoo* | MAI133 | S11_28896289 | 6.8E-04 | 3.4E-01 | 5.8 | -1.5 | A/G | 153/61 | * | LOC_Os11g47910 | SCARECROW, putative, expressed |

**Continuation Table S5.**

| **Pathovar** | **Strain** | **Marker** | **p-value** | **q-value** | **R^2^** | **Effect** | **Effect** | **AILs with Effect** | **Significance** | **Locus** | **Annotation** |
| --- | --- | --- | --- | --- | --- | --- | --- | --- | --- | --- | --- |
|  |  | **(Chr_Pos)** |  |  |  | **estimate** | **allele/Null** | **allele/Null** | **correspondence** |  |  |
| *Xoo* | MAI133 | S11_28904361 | 9.0E-04 | 3.5E-01 | 4.6 | -1.4 | T/C | 179/67 | * | LOC_Os11g47930 | alpha-hemolysin, putative, expressed |
| *Xoc* | BLS256 | **S11_28920280** | 7.5E-05 | 6.7E-02 | 7.2 | -10.8 | T/A | 197/32 | * | LOC_Os11g47944 | thaumatin, putative, expressed |
| *Xoo* | BAI3 | **S11_28920280** | 7.6E-04 | 1.0E-01 | 4.0 | -4.5 | T/A | 244/37 | * | LOC_Os11g47944 | thaumatin, putative, expressed |
| *Xoo* | MAI101 | **S11_28920280** | 3.7E-04 | 1.4E-01 | 6.1 | -4.2 | T/A | 201/33 | * | LOC_Os11g47944 | thaumatin, putative, expressed |
| *Xoo* | MAI133 | **S11_28920280** | 8.2E-04 | 3.4E-01 | 4.7 | -2.0 | T/A | 199/30 | * | LOC_Os11g47944 | thaumatin, putative, expressed |
| *Xoo* | MAI145 | **S11_28920280** | 8.5E-05 | 5.1E-02 | 7.4 | -3.9 | T/A | 212/33 | * | LOC_Os11g47944 | thaumatin, putative, expressed |
| *Xoo* | BAI3 | **S11_28920303** | 8.8E-04 | 1.2E-01 | 3.8 | -5.9 | C/A | 267/17 | * | LOC_Os11g47944 | thaumatin, putative, expressed |
| *Xoo* | MAI145 | **S11_28920303** | 6.6E-04 | 2.3E-01 | 5.4 | -4.4 | C/A | 231/17 | * | LOC_Os11g47944 | thaumatin, putative, expressed |
| *Xoo* | BAI3 | **S11_28920325** | 6.0E-04 | 8.9E-02 | 3.8 | -6.0 | C/G | 295/17 | * | LOC_Os11g47944 | thaumatin, putative, expressed |
| *Xoo* | MAI145 | **S11_28920325** | 5.0E-04 | 2.1E-01 | 4.5 | -4.6 | C/G | 259/16 | * | LOC_Os11g47944 | thaumatin, putative, expressed |
| *Xoo* | MAI133 | S11_28938622 | 3.0E-04 | 3.4E-01 | 6.4 | -3.1 | G/A | 212/13 | * | LOC_Os11g47980 | expressed protein |
| *Xoo* | BAI3 | **S11_28977691** | 5.4E-06 | 2.5E-03 | 7.3 | -6.0 | T/C | 264/42 | *** | intergenic |  |
| *Xoo* | MAI145 | **S11_28977691** | 1.2E-04 | 6.5E-02 | 5.7 | -3.7 | T/C | 232/37 | * | intergenic |  |
|  |  |  |  |  |  |  |  |  |  |  |  |
| *Xoo* | BAI3 | S12_2195904 | 8.2E-04 | 1.1E-01 | 4.0 | 2.9 | T/C | 137/156 | * | LOC_Os12g05040 | heavy-metal-associated domain-containing protein, putative, expressed |
| *Xoo* | BAI3 | S12_2203648 | 5.2E-04 | 8.3E-02 | 4.1 | 2.8 | G/A | 172/115 | * | intergenic |  |
|  |  |  |  |  |  |  |  |  |  |  |  |

| **Pathovar** | **Strain** | **Chr** | **QTL** | **QTL position** | | | **Supporting interval** | | **Interval size** | | **Wald** | **p-value** | **Predicted parental source** | **Corroboration** |
| --- | --- | --- | --- | --- | --- | --- | --- | --- | --- | --- | --- | --- | --- | --- |
|  |  |  | **name** | **(cM)** | **Left Mrk** | **Right Mrk** | **Left Mrk** | **Right Mrk** | **(cM)** | **(kb)** |  |  | **of resistance** | **GWAS** |
| *Xoo* | BAI3 | 1 |  | 124.4 | S1_31010757 | S1_31182879 | S1_28664444 | S1_32112121 | 13.8 | 3,448 | 27.8 | 2.E-04 | FED, IR45, IR46 |  |
|  |  |  |  |  |  |  |  |  |  |  |  |  |  |  |
| *Xoc* | MAI3 | 2 | qXO-2-2 | 141.6 | S2_35477326 | S2_35580048 | S2_35342151 | S2_35891034 | 2.2 | 549 | 25.3 | 7.E-04 | FED, PSBRc158 |  |
|  |  |  |  |  |  |  |  |  |  |  |  |  |  |  |
| *Xoo* | BAI3 | 3 |  | 55.0 | S3_13969997 | S3_14144630 | S3_12659394 | S3_14658353 | 8.0 | 1,999 | 32.3 | 4.E-05 | FED, SHZ-2 |  |
| *Xoc* | MAI3 | 3 |  | 133.0 | S3_33208259 | S3_33647311 | S3_32013647 | S3_34825996 | 11.2 | 2,812 | 24.3 | 1.E-03 | FED, IR45, IR46, PSBRc82, SHZ-2 | |
|  |  |  |  |  |  |  |  |  |  |  |  |  |  |  |
| *Xoc* | MAI3 | 4 | qXO-4-1 | 122.2 | S4_30383295 | S4_30738888 | S4_30030316 | S4_31803018 | 7.1 | 1,773 | 30.0 | 1.E-04 | IR45, IR46, SHZ-2 |  |
| *Xoc* | BAI5 | 4 |  | 123.6 | S4_30738922 | S4_31079998 | S4_30383295 | S4_31882603 | 6.0 | 1,499 | 48.4 | 3.E-08 | FED, IR46, SHZ-2 | * |
| *Xoo* | BAI3 | 4 |  | 126.1 | S4_31666827 | S4_31716597 | S4_30383295 | S4_31992079 | 6.4 | 1,609 | 56.1 | 9.E-10 | IR46, PSBRc82 | * |
|  |  |  |  |  |  |  |  |  |  |  |  |  |  |  |
| *Xoo* | BAI3 | 5 | qXO-5-2 | 4.0 | S5_1065077 | S5_1073143 | S5_761076 | S5_1258099 | 2.0 | 497 | 47.4 | 5.E-08 | PSBRc82 | * |
| *Xoc* | BAI5 | 5 |  | 9.0 | S5_2312513 | S5_2821285 | S5_2062346 | S5_4184550 | 8.5 | 2,122 | 27.1 | 3.E-04 | All |  |
|  |  |  |  |  |  |  |  |  |  |  |  |  |  |  |
| *Xoc* | MAI3 | 7 |  | 1.0 | S7_1000810 | S7_1032993 | S7_756131 | S7_1433062 | 2.7 | 677 | 35.1 | 1.E-05 | All |  |
|  |  |  |  |  |  |  |  |  |  |  |  |  |  |  |
| *Xoc* | BAI5 | 8 |  | 20.9 | S8_5373265 | S8_5418149 | S8_4491493 | S8_6277593 | 7.1 | 1,786 | 30.8 | 7.E-05 | All except SHZ-2 | * |
| *Xoo* | BAI3 | 8 |  | 89.8 | S8_21378692 | S8_22606500 | S8_20188025 | S8_24097520 | 15.6 | 3,909 | 27.2 | 3.E-04 | IR45, IR46 |  |
|  |  |  |  |  |  |  |  |  |  |  |  |  |  |  |
| *Xoo* | MAI1 | 9 |  | 48.6 | S9_12636353 | S9_14163560 | S9_12086978 | S9_14163560 | 8.3 | 2,077 | 24.4 | 1.E-03 | IR46 | * |
| *Xoo* | BAI3 | 9 |  | 83.0 | S9_21176469 | S9_21243301 | S9_20977131 | S9_21546286 | 2.3 | 569 | 33.0 | 3.E-05 | IR46 | * |
|  |  |  |  |  |  |  |  |  |  |  |  |  |  |  |
| *Xoc* | BAI5 | 10 | qXO-10-1 | 76.0 | S10_19063585 | S10_19107872 | S10_18851296 | S10_19354685 | 2.0 | 503 | 25.0 | 8.E-04 | IR45 |  |
| *Xoo* | BAI3 | 10 |  | 89.7 | S10_21573347 | S10_22502006 | S10_20993968 | S10_22911489 | 7.7 | 1,918 | 30.0 | 9.E-05 | FED, IR77, PSBRc158, PSBRc82 | |
|  |  |  |  |  |  |  |  |  |  |  |  |  |  |  |
| *Xoc* | BAI5 | 11 |  | 95.9 | S11_23983620 | S11_24295017 | S11_23558018 | S11_24809810 | 5.0 | 1,252 | 31.1 | 6.E-05 | All except FED |  |
|  |  |  |  |  |  |  |  |  |  |  |  |  |  |  |
| *Xoc* | MAI3 | 11 | qXO-11-2 | 112.5 | S11_28116388 | S11_28483934 | S11_27573854 | S11_28988578 | 5.7 | 1,415 | 37.0 | 5.E-06 | All except IR77 | * |
| *Xoo* | BAI3 | 11 |  | 114.6 | S11_28483987 | S11_28652283 | S11_27573854 | S11_28988578 | 5.7 | 1,415 | 41.8 | 6.E-07 | FED, IR45, IR46, PSBRc158, SHZ-2 | * |

**Table S6**. QTL detection for resistance to African *Xoc* and *Xoo* in MAGIC indica S4 subset (*P*-value < 0.001), organized by chromosome. Wald statistic for test that no parents have effects on trait (Wald, 7 df). Parental source of resistance determined by founder effects estimates relative to Sambha Mahsuri + Sub1. Stars in significance correspondence panel indicate level of significance of GWAS (*) *P* value < 0.001 and q-value > 0.05.

**Table S7**. QTL detection for resistance to African and Asian *Xoc* and *Xoo* in MAGIC indica S8 subset (*P*-value < 0.001), organized by chromosome. Wald statistic for test that no parents have effects on trait (Wald, 7 df). Parental source of resistance determined by founder effects estimates relative to Sambha Mahsuri + Sub1; nd = non-determined. Stars in corroboration GWA panel indicate level of significance of GWAS markers as follows: (*) *P*-value < 0.001 and q-value > 0.05; (***) *P*-value < 0.0001 and q-value < 0.05. Known resistance column refers to previously identified QTL and single resistance genes (denoted by the prefix *Xa* or *Xo*).

| **Pathovar** | **Strain** | **Chr** | **QTL** | **QTL position** | | | **Supporting interval** | | **Interval size** | | **Wald** | **p-value** | **Phenotypic** | **Predicted parental source** | **Corroboration** | **Known resistance loci to** | |
| --- | --- | --- | --- | --- | --- | --- | --- | --- | --- | --- | --- | --- | --- | --- | --- | --- | --- |
|  |  |  |  | **(cM)** | **Left Mrk** | **Right Mrk** | **Left Mrk** | **Right Mrk** | **(cM)** | **(kb)** |  |  | **variance (%)** | **of resistance** | **GWAS** | **African**  ***X. oryzae* strains** | **Asian**  ***X. oryzae* strains** |
| *Xoo* | MAI72 | 1 | qXO-1-1 | 5.2 | S1_1335951 | S1_1336798 | S1_1264553 | S1_1511717 | 1.0 | 247 | 33.9 | 1.8E-05 | 8.4 | nd |  |  |  |
| *Xoo* | MAI130 | 1 |  | 5.4 | S1_1365841 | S1_1375634 | S1_1336959 | S1_1516299 | 0.7 | 179 | 47.8 | 4.0E-08 | 12.1 | nd |  |  |  |
| *Xoc* | MAI123 | 1 |  | 5.9 | S1_1401021 | S1_1500023 | S1_1365711 | S1_1550887 | 0.7 | 185 | 30.7 | 7.1E-05 | 8.2 | nd |  |  |  |
|  |  |  |  |  |  |  |  |  |  |  |  |  |  |  |  |  |  |
| *Xoo* | MAI134 | 1 |  | 44.9 | S1_11258688 | S1_11272858 | S1_11078476 | S1_11531550 | 1.8 | 453 | 30.2 | 8.6E-05 | 7.3 | PSBRc158 |  | qABB-1 |  |
| *Xoo* | MAI134 | 1 |  | 56.3 | S1_14102698 | S1_14174257 | S1_13542393 | S1_14346639 | 3.2 | 804 | 28.8 | 1.6E-04 | 6.9 | PSBRc158 |  | qABB-1 |  |
|  |  |  |  |  |  |  |  |  |  |  |  |  |  |  |  |  |  |
| *Xoc* | BAI5 | 2 |  | 17.6 | S2_4440042 | S2_4553565 | S2_4150417 | S2_4868450 | 2.9 | 718 | 27.1 | 3.2E-04 | 5.9 | FED, IR45, IR46 |  |  |  |
| *Xoc* | MAI46 | 2 |  | 19.9 | S2_5030179 | S2_5044670 | S2_4950345 | S2_5044670 | 0.4 | 94 | 27.8 | 2.4E-04 | 6.8 | IR46, SHZ-2 |  |  |  |
| *Xoo* | MAI134 | 2 |  | 26.6 | S2_6651629 | S2_6706358 | S2_6246858 | S2_6778045 | 2.1 | 531 | 27.8 | 2.4E-04 | 6.6 | IR45 |  |  |  |
| *Xoc* | MAI61 | 2 |  | 92.2 | S2_22897004 | S2_23098527 | S2_22249726 | S2_23384851 | 4.5 | 1135 | 25.5 | 6.1E-04 | 6.5 | IR45, IR46, IR77, PSBRc82, SHZ-2 | |  | Qbr2a |
|  |  |  |  |  |  |  |  |  |  |  |  |  |  |  |  |  |  |
| *Xoc* | MAI77 | 2 | qXO-2-1 | 96.3 | S2_24122049 | S2_24133875 | S2_24021185 | S2_24165677 | 0.6 | 144 | 33.9 | 1.8E-05 | 8.8 | IR45, IR46,IR77 | SNPs near interval (*) |  | Qbr2a |
| *Xoc* | MAI46 | 2 |  | 96.9 | S2_24278085 | S2_24278919 | S2_24210631 | S2_24387205 | 0.7 | 177 | 33.7 | 2.0E-05 | 8.6 | IR45, IR77, PSBRc82, SHZ-2 | * |  | Qbr2a |
| *Xoc* | MAI139 | 2 |  | 100.9 | S2_25272641 | S2_25273930 | S2_25212962 | S2_25305176 | 0.4 | 92 | 41.0 | 8.0E-07 | 12.0 | IR45, IR46, IR77, SHZ-2 | SNPs near interval (*) |  |  |
| *Xoc* | MAI10 | 2 |  | 101.1 | S2_25332318 | S2_25351909 | S2_23819009 | S2_27466604 | 14.6 | 3648 | 30.3 | 8.3E-05 | 7.5 | FED, IR77, SHZ-2 | * |  | Qbr2a |
| *Xoc* | BLS256 | 2 |  | 103.3 | S2_25869421 | S2_25885474 | S2_25698342 | S2_26335825 | 2.5 | 637 | 44.5 | 1.7E-07 | 12.3 | All except PSBRc158 | *** |  |  |
| *Xoc* | MAI61 | 2 |  | 104.2 | S2_26097152 | S2_26101866 | S2_25510717 | S2_26467059 | 3.8 | 956 | 26.9 | 3.5E-04 | 6.9 | FED, IR45, IR46, IR77 | * |  |  |
| *Xoc* | BAI5 | 2 |  | 104.3 | S2_26101866 | S2_26113224 | S2_25698342 | S2_26375084 | 2.7 | 677 | 31.8 | 4.5E-05 | 7.1 | FED,IR77, SHZ-2 | * |  |  |
| *Xoo* | MAI130 | 2 |  | 107.8 | S2_26854556 | S2_26993900 | S2_26854556 | S2_27203917 | 1.4 | 349 | 31.8 | 4.5E-05 | 7.7 | nd |  |  |  |
|  |  |  |  |  |  |  |  |  |  |  |  |  |  |  |  |  |  |
| *Xoo* | MAI101 | 2 |  | 115.0 | S2_28802779 | S2_28808167 | S2_28788926 | S2_28828402 | 0.2 | 39 | 27.7 | 2.5E-04 | 7.1 | IR45, IR46, IR77 |  |  |  |

**Continuation Table S7**.

| **Pathovar** | **Strain** | **Chr** | **QTL** | **QTL position** | | | **Supporting interval** | | **Interval size** | | **Wald** | **p-value** | **Phenotypic** | **Predicted parental source** | **Corroboration** | **Known resistance loci to** | |
| --- | --- | --- | --- | --- | --- | --- | --- | --- | --- | --- | --- | --- | --- | --- | --- | --- | --- |
|  |  |  |  | **(cM)** | **Left Mrk** | **Right Mrk** | **Left Mrk** | **Right Mrk** | **(cM)** | **(kb)** |  |  | **variance (%)** | **of resistance** | **GWAS** | **African**  ***X. oryzae* strains** | **Asian**  ***X. oryzae* strains** |
| *Xoc* | MAI139 | 2 | qXO-2-2 | 141.1 | S2_35289602 | S2_35313442 | S2_35068072 | S2_35589943 | 2.1 | 522 | 26.5 | 4.2E-04 | 7.2 | nd |  |  | AQBT001, qBbr2b |
| *Xoo* | MAI130 | 2 |  | 142.4 | S2_35642187 | S2_35659739 | S2_35140697 | S2_35891362 | 3.0 | 751 | 28.2 | 2.1E-04 | 6.7 | SHZ-2 | * |  | AQBT001, qBbr2b, *Xa24(t)* |
| *Xoo* | MAI101 | 2 |  | 142.9 | S2_35772697 | S2_35781025 | S2_35642187 | S2_35891364 | 1.0 | 249 | 33.6 | 2.1E-05 | 9.0 | nd |  |  | AQBT001, *Xa24(t)* |
|  |  |  |  |  |  |  |  |  |  |  |  |  |  |  |  |  |  |
| *Xoo* | MAI134 | 3 |  | 6.4 | S3_1796192 | S3_1818201 | S3_1270943 | S3_2093081 | 3.3 | 822 | 34.4 | 1.4E-05 | 8.5 | nd |  |  | AQBT002 |
| *Xoo* | MAI93 | 3 |  | 58.1 | S3_14739507 | S3_14760067 | S3_10841565 | S3_15864307 | 20.1 | 5023 | 28.3 | 1.9E-04 | 11.4 | FED |  |  | qBB3-1 (AQW002) |
| *Xoo* | MAI72 | 3 |  | 81.7 | S3_20646866 | S3_20691702 | S3_20568320 | S3_20691702 | 0.5 | 123 | 26.8 | 3.6E-04 | 6.3 | All |  |  |  |
|  |  |  |  |  |  |  |  |  |  |  |  |  |  |  |  |  |  |
| *Xoc* | MAI10 | 4 |  | 52.3 | S4_13164480 | S4_13173499 | S4_13000541 | S4_14723818 | 6.9 | 1723 | 26.6 | 4.0E-04 | 6.3 | All except PSBRc82 |  |  | qBbr4a |
| *Xoc* | MAI61 | 4 |  | 77.0 | S4_19349782 | S4_19375312 | S4_19233155 | S4_19711091 | 1.9 | 478 | 25.7 | 5.6E-04 | 6.5 | nd |  |  |  |
| *Xoo* | MAI145 | 4 |  | 79.7 | S4_20042539 | S4_20065837 | S4_20027337 | S4_20090957 | 0.3 | 64 | 30.9 | 6.5E-05 | 7.7 | IR45, IR46, IR77, PSBRc82 |  |  |  |
| *Xoc* | MAI10 | 4 |  | 80.4 | S4_20195543 | S4_20213857 | S4_20167660 | S4_20246699 | 0.3 | 79 | 49.0 | 2.3E-08 | 12.7 | nd |  |  |  |
| *Xoo* | MAI136 | 4 |  | 81.1 | S4_20382464 | S4_20389318 | S4_20381546 | S4_20394209 | 0.1 | 13 | 32.2 | 3.8E-05 | 8.4 | IR45, IR77, PSBRc82, SHZ-2 | |  |  |
| *Xoo* | CFBP1951 | 4 |  | 83.1 | S4_20892614 | S4_20904803 | S4_20863240 | S4_20927918 | 0.3 | 65 | 28.2 | 2.0E-04 | 7.0 | nd |  |  |  |
| *Xoc* | MAI67 | 4 |  | 85.4 | S4_21464884 | S4_21470998 | S4_21448290 | S4_21498207 | 0.2 | 50 | 35.1 | 1.1E-05 | 9.1 | IR46 |  |  | qBbr4b |
| *Xoo* | MAI72 | 4 |  | 99.0 | S4_24504517 | S4_24863454 | S4_24071697 | S4_26519264 | 9.8 | 2448 | 26.8 | 3.6E-04 | 6.3 | SHZ-2 |  |  | qBbr4b, qBB-4 |
| *Xoo* | CFBP1951 | 4 |  | 117.7 | S4_29505127 | S4_29526391 | S4_29247633 | S4_29883989 | 2.5 | 636 | 29.0 | 1.4E-04 | 7.3 | nd |  |  |  |
| *Xoc* | MAI46 | 4 |  | 123.9 | S4_31080257 | S4_31127321 | S4_30862506 | S4_31205433 | 1.4 | 343 | 29.3 | 1.3E-04 | 7.3 | IR46, SHZ-2 |  |  |  |

**Continuation Table S7**.

| **Pathovar** | **Strain** | **Chr** | **QTL** | **QTL position** | | | **Supporting interval** | | **Interval size** | | **Wald** | **p-value** | **Phenotypic** | **Predicted parental source** | **Corroboration** | **Known resistance loci to** | |
| --- | --- | --- | --- | --- | --- | --- | --- | --- | --- | --- | --- | --- | --- | --- | --- | --- | --- |
|  |  |  |  | **(cM)** | **Left Mrk** | **Right Mrk** | **Left Mrk** | **Right Mrk** | **(cM)** | **(kb)** |  |  | **variance (%)** | **of resistance** | **GWAS** | **African**  ***X. oryzae* strains** | **Asian**  ***X. oryzae***  **strains** |
| *Xoo* | MAI145 | 4 | qXO-4-1 | 125.8 | S4_31553264 | S4_31559540 | S4_30862506 | S4_31803141 | 3.8 | 941 | 28.4 | 1.9E-04 | 7.0 | FED, IR46 | *** | *Xo1* | *Xa1, Xa2, Xa31(t), Xa38* |
| *Xoo* | CFBP1951 | 4 |  | 126.5 | S4_31728040 | S4_31728342 | S4_31721763 | S4_31782187 | 0.2 | 60 | 38.0 | 3.0E-06 | 10.0 | nd | * | *Xo1* | *Xa2* |
| *Xoc* | MAI10 | 4 |  | 126.8 | S4_31803168 | S4_31812015 | S4_31531452 | S4_31872983 | 1.4 | 342 | 25.2 | 7.0E-04 | 7.7 | IR46, SHZ-2 | *** | *Xo1* | AQBT008, *Xa1, Xa2, Xa38* |
| *Xoc* | MAI67 | 4 |  | 126.8 | S4_31803168 | S4_31812015 | S4_31449324 | S4_32064419 | 2.5 | 615 | 27.9 | 2.3E-04 | 8.5 | IR46 | *** | *Xo1* | AQBT008, *Xa1, Xa2, Xa31(t), Xa38* |
| *Xoo* | BAI3 | 4 |  | 126.8 | S4_31803168 | S4_31812015 | S4_31506827 | S4_31900997 | 1.6 | 394 | 90.3 | 1.1E-16 | 20.8 | FED, IR46, SHZ-2 | *** | *Xo1* | AQBT008, *Xa1, Xa2, Xa38* |
| *Xoo* | MAI70 | 4 |  | 126.8 | S4_31803168 | S4_31812015 | S4_31506827 | S4_31872983 | 1.5 | 366 | 45.9 | 9.1E-08 | 11.8 | FED, IR45, IR46, SHZ-2 | *** | *Xo1* | AQBT008, *Xa1, Xa2, Xa38* |
| *Xoc* | MAI139 | 4 |  | 126.8 | S4_31803168 | S4_31812015 | S4_31553238 | S4_32134600 | 2.3 | 581 | 40.2 | 1.2E-06 | 11.7 | IR45, IR46, IR77, PSBRc82, SHZ-2 | * | *Xo1* | AQBT008, *Xa1, Xa2, Xa38* |
| *Xoc* | BAI5 | 4 |  | 126.8 | S4_31819592 | S4_31872983 | S4_31786637 | S4_31872983 | 0.3 | 86 | 216.9 | 3.0E-43 | 39.5 | IR46, SHZ-2 | *** | *Xo1* | AQBT008, *Xa2* |
| *Xoc* | MAI77 | 4 |  | 126.8 | S4_31819592 | S4_31872983 | S4_31803141 | S4_31956274 | 0.6 | 153 | 34.9 | 1.2E-05 | 9.1 | IR46, SHZ-2 | * | *Xo1* | AQBT008, *Xa2* |
| *Xoo* | MAI130 | 4 |  | 127.2 | S4_31873066 | S4_31900997 | S4_31531452 | S4_32134600 | 2.4 | 603 | 47.3 | 5.0E-08 | 12.0 | IR46 | *** | *Xo1* | AQBT008, *Xa1, Xa2, Xa38* |
| *Xoo* | MAI101 | 4 |  | 127.5 | S4_31992079 | S4_32007187 | S4_31559582 | S4_32134600 | 2.3 | 575 | 31.7 | 4.6E-05 | 8.4 | FED, IR46, IR77, SHZ-2 | *** | *Xo1* | AQBT008, *Xa1, Xa2, Xa38* |
| *Xoo* | MAI136 | 4 |  | 127.7 | S4_32037850 | S4_32064419 | S4_31819592 | S4_32134600 | 1.3 | 315 | 31.6 | 4.7E-05 | 8.22 | All except PSBRc158 | *** | *Xo1* | AQBT008, *Xa2* |
|  |  |  |  |  |  |  |  |  |  |  |  |  |  |  |  |  |  |
| *Xoc* | MAI46 | 4 |  | 133.3 | S4_33428983 | S4_33431106 | S4_32681859 | S4_33498618 | 3.3 | 817 | 27.0 | 3.3E-04 | 7.7 | FED, IR45, SHZ-2 |  |  | *Xa2* |
|  |  |  |  |  |  |  |  |  |  |  |  |  |  |  |  |  |  |
| *Xoc* | MAI61 | 5 | qXO-5-1 | 0.1 | S5_69530 | S5_87749 | S5_37878 | S5_162688 | 0.5 | 125 | 25.9 | 5.2E-04 | 6.6 | IR45, IR46, IR77, SHZ-2 | * |  |  |
| *Xoo* | BAI3 | 5 |  | 1.3 | S5_347328 | S5_353165 | S5_37878 | S5_453169 | 1.7 | 415 | 45.2 | 1.3E-07 | 10.5 | IR45,PSBRc82,SHZ-2 | *** |  | qBLSr5a, qBbr5, *xa5* |
|  |  |  |  |  |  |  |  |  |  |  |  |  |  |  |  |  |  |
| *Xoc* | MAI46 | 5 | qXO-5-2 | 5.9 | S5_1494420 | S5_1503830 | S5_1200971 | S5_1729230 | 2.1 | 528 | 25.9 | 5.2E-04 | 6.2 | IR45, IR46, IR77, PSBRc158, SHZ-2 | * |  |  |
| *Xoc* | MAI61 | 5 |  | 8.0 | S5_2027760 | S5_2046183 | S5_1027632 | S5_2167880 | 4.6 | 1140 | 24.8 | 8.2E-04 | 6.2 | All except PSBRc158 |  |  |  |
|  |  |  |  |  |  |  |  |  |  |  |  |  |  |  |  |  |  |
| *Xoo* | CFBP1951 | 5 |  | 26.6 | S5_6637584 | S5_6678610 | S5_6637583 | S5_6722685 | 0.3 | 85 | 27.0 | 3.3E-04 | 6.7 | FED | SNPs near interval (*) |  |  |
|  |  |  |  |  |  |  |  |  |  |  |  |  |  |  |  |  |  |
| *Xoo* | MAI134 | 5 | qXO-5-3 | 84.9 | S5_21255253 | S5_21351387 | S5_20924798 | S5_21471465 | 2.2 | 547 | 36.0 | 7.2E-06 | 9.0 | nd | *** |  | qBB-5-2 |
| *Xoc* | BLS256 | 5 |  | 90.7 | S5_22720353 | S5_22750867 | S5_21141624 | S5_24383716 | 13.0 | 3242 | 25.8 | 5.6E-04 | 6.6 | FED, IR46, PSBRc82,SHZ-2 | |  | qBB5, qBB-5-2, AQW004, qBBR5 |

**Continuation Table S7**

| **Pathovar** | **Strain** | **Chr** | **QTL** | **QTL position** | | | **Supporting interval** | | **Interval size** | | **Wald** | **p-value** | **Phenotypic** | **Predicted parental source** | **Corroboration** | **Known resistance loci to** | |
| --- | --- | --- | --- | --- | --- | --- | --- | --- | --- | --- | --- | --- | --- | --- | --- | --- | --- |
|  |  |  |  | **(cM)** | **Left Mrk** | **Right Mrk** | **Left Mrk** | **Right Mrk** | **(cM)** | **(kb)** |  |  | **variance (%)** | **of resistance** | **GWAS** | **African**  ***X. oryzae* strains** | **Asian**  ***X. oryzae* strains** |
| *Xoo* | MAI101 | 6 |  | 100.0 | S6_25124153 | S6_25144615 | S6_24427437 | S6_25422894 | 4.0 | 995 | 28.4 | 1.9E-04 | 7.4 | IR45, IR46, PSBRc158, SHZ-2 | |  |  |
|  |  |  |  |  |  |  |  |  |  |  |  |  |  |  |  |  |  |
| *Xoo* | MAI136 | 7 | qBB-7-1 | 20.11 | S7_5097414 | S7_5129087 | S7_3622979 | S7_6502570 | 11.5 | 2880 | 31.9 | 4.2E-05 | 8.31 | FED |  | qABB-7 | *xa8* |
| *Xoo* | CFBP1951 | 7 |  | 23.57 | S7_5956894 | S7_5993972 | S7_5956894 | S7_6502570 | 2.2 | 546 | 65.5 | 1.2E-11 | 17.3 | nd | *** | qABB-7 |  |
|  |  |  |  |  |  |  |  |  |  |  |  |  |  |  |  |  |  |
| *Xoo* | CFBP1951 | 7 |  | 81.62 | S7_20506279 | S7_20525028 | S7_20393784 | S7_20525704 | 0.5 | 132 | 27.9 | 2.3E-04 | 7.0 | FED |  |  |  |
|  |  |  |  |  |  |  |  |  |  |  |  |  |  |  |  |  |  |
| *Xoo* | MAI72 | 7 | qXO-7-2 | 109.9 | S7_27578266 | S7_27585272 | S7_27479742 | S7_27619799 | 0.6 | 140 | 31.2 | 5.7E-05 | 7.6 | nd | * |  |  |
| *Xoc* | MAI61 | 7 |  | 112.3 | S7_28165258 | S7_28179129 | S7_27614620 | S7_28469553 | 3.4 | 855 | 36.0 | 7.3E-06 | 9.7 | FED, IR77 |  |  |  |
|  |  |  |  |  |  |  |  |  |  |  |  |  |  |  |  |  |  |
| *Xoo* | MAI101 | 8 |  | 4.2 | S8_1074851 | S8_1090318 | S8_760745 | S8_1758774 | 4.0 | 998 | 25.4 | 6.5E-04 | 6.4 | IR45, IR46, PSBRc82 |  |  |  |
| *Xoc* | MAI139 | 8 |  | 20.7 | S8_5140943 | S8_5217372 | S8_4153941 | S8_5318021 | 4.7 | 1164 | 29.2 | 1.3E-04 | 8.1 | IR45, IR46, IR77, PSBRc82, SHZ-2 | |  |  |
| *Xoo* | CFBP1951 | 8 |  | 98.7 | S8_24733521 | S8_24833706 | S8_24640146 | S8_24947837 | 1.2 | 308 | 37.8 | 3.3E-06 | 9.9 | IR77 |  |  | qBbr8b |
|  |  |  |  |  |  |  |  |  |  |  |  |  |  |  |  |  |  |
| *Xoc* | MAI123 | 8 | qBLS-8-1 | 102.6 | S8_25638183 | S8_25700349 | S8_25638183 | S8_25937369 | 1.2 | 299 | 26.2 | 4.6E-04 | 6.8 | IR45 |  |  | qBbr8b |
| *Xoc* | MAI77 | 8 |  | 102.7 | S8_25700349 | S8_25729831 | S8_25638183 | S8_26014015 | 1.5 | 376 | 30.0 | 9.4E-05 | 7.6 | IR45 |  |  | qBbr8b |
|  |  |  |  |  |  |  |  |  |  |  |  |  |  |  |  |  |  |
| *Xoo* | MAI70 | 9 |  | 34.1 | S9_9220532 | S9_9340981 | S9_9220532 | S9_9344787 | 0.5 | 124 | 35.4 | 9.5E-06 | 9.4 | IR46, PSBRc82, PSBRc158, SHZ-2 | |  |  |
| *Xoo* | CFBP1951 | 9 |  | 41.9 | S9_11299373 | S9_11318792 | S9_11230720 | S9_11318792 | 0.4 | 88 | 53.4 | 3.0E-09 | 14.2 | nd |  |  |  |
| *Xoc* | MAI123 | 9 |  | 47.3 | S9_12629477 | S9_12636353 | S9_12628300 | S9_12699426 | 0.3 | 71 | 29.6 | 1.1E-04 | 7.9 | IR46 |  |  |  |
| *Xoo* | MAI145 | 9 |  | 82.4 | S9_21390442 | S9_21418696 | S9_21317041 | S9_21501670 | 0.7 | 185 | 28.0 | 2.2E-04 | 6.9 | IR45 |  |  |  |

**Continuation Table S7**

| **Pathovar** | **Strain** | **Chr** | **QTL** | **QTL position** | | | **Supporting interval** | | **Interval size** | | **Wald** | **p-value** | **Phenotypic** | **Predicted parental source** | **Corroboration** | **Known resistance loci to** | |
| --- | --- | --- | --- | --- | --- | --- | --- | --- | --- | --- | --- | --- | --- | --- | --- | --- | --- |
|  |  |  |  | **(cM)** | **Left Mrk** | **Right Mrk** | **Left Mrk** | **Right Mrk** | **(cM)** | **(kb)** |  |  | **variance (%)** | **of resistance** | **GWAS** | **African**  ***X. oryzae* strains** | **Asian**  ***X. oryzae***  **strains** |
| *Xoo* | MAI136 | 10 |  | 36.4 | S10_8918195 | S10_9170996 | S10_7551300 | S10_10628934 | 12.3 | 3078 | 25.1 | 7.2E-04 | 6.2 | FED, SHZ-2 |  |  | qBbr10, QBbr10, qBBR10 |
| *Xoc* | BAI5 | 10 |  | 46.2 | S10_11626385 | S10_11634014 | S10_11575914 | S10_11743048 | 0.7 | 167 | 28.4 | 1.9E-04 | 6.2 | IR45, IR46, SHZ-2 |  |  |  |
| *Xoo* | CFBP1951 | 10 |  | 59.6 | S10_14985398 | S10_15006373 | S10_14926494 | S10_15168059 | 1.0 | 242 | 35.2 | 1.0E-05 | 9.1 | nd |  |  |  |
|  |  |  |  |  |  |  |  |  |  |  |  |  |  |  |  |  |  |
| *Xoc* | MAI10 | 10 | qXO-10-1 | 79.6 | S10_19975243 | S10_20082337 | S10_19903199 | S10_20082337 | 0.7 | 179 | 28.8 | 1.6E-04 | 7.0 | IR45, IR77 |  | qABB-10 |  |
| *Xoc* | MAI46 | 10 |  | 79.6 | S10_19975243 | S10_20082337 | S10_19916740 | S10_20082337 | 0.7 | 166 | 40.9 | 8.6E-07 | 10.6 | IR45 |  | qABB-10 |  |
| *Xoo* | CFBP1951 | 10 |  | 80.8 | S10_20275339 | S10_20983368 | S10_19975243 | S10_20983368 | 4.0 | 1008 | 56.0 | 9.4E-10 | 14.9 | nd |  | qABB-10 |  |
|  |  |  |  |  |  |  |  |  |  |  |  |  |  |  |  |  |  |
| *Xoo* | MAI101 | 10 |  | 88.1 | S10_21966227 | S10_22098921 | S10_21786349 | S10_22502019 | 2.9 | 716 | 36.7 | 5.4E-06 | 9.9 | IR45, IR46, IR77, PSBRc82 |  | qABB-10 |  |
|  |  |  |  |  |  |  |  |  |  |  |  |  |  |  |  |  |  |
| *Xoo* | BAI3 | 11 | qBB-11-1 | 28.0 | S11_7012013 | S11_7013171 | S11_6647060 | S11_7438223 | 3.2 | 791 | 27.0 | 3.4E-04 | 5.8 | FED, IR77, PSBRc158, SHZ-2 | * |  |  |
| *Xoo* | MAI70 | 11 |  | 28.9 | S11_7221827 | S11_7244498 | S11_6647060 | S11_7616946 | 3.9 | 970 | 26.2 | 4.7E-04 | 5.9 | FED, IR77 | *** |  |  |
|  |  |  |  |  |  |  |  |  |  |  |  |  |  |  |  |  |  |
| *Xoc* | BLS256 | 11 | qXO-11-2 | 107.5 | S11_26879946 | S11_26950070 | S11_26393474 | S11_27412501 | 4.1 | 1019 | 36.4 | 6.2E-06 | 9.9 | All | *** | qABB-11 | QBbr11, *Xa22, Xa35(t)* |
| *Xoo* | MAI72 | 11 |  | 108.1 | S11_27015438 | S11_27033636 | S11_26981276 | S11_27205864 | 0.9 | 225 | 33.3 | 2.3E-05 | 8.2 | All |  | qABB-11 | QBbr11, *Xa22, Xa35(t)* |
| *Xoo* | MAI101 | 11 |  | 108.7 | S11_27169283 | S11_27176255 | S11_26981276 | S11_27205864 | 0.9 | 225 | 39.1 | 1.9E-06 | 10.7 | All | *** | qABB-11 | QBbr11, *Xa22, Xa35(t)* |
| *Xoo* | CFBP1951 | 11 |  | 109.6 | S11_27393912 | S11_27412501 | S11_27192125 | S11_27574157 | 1.5 | 382 | 25.8 | 5.4E-04 | 6.3 | All except IR46 |  | qABB-11 | QBbr11, *Xa22, Xa35(t)* |
| *Xoo* | MAI145 | 11 |  | 112.5 | S11_28121355 | S11_28483934 | S11_27831677 | S11_28697250 | 3.5 | 866 | 52.0 | 5.9E-09 | 13.6 | All | *** | qABB-11 | *Xa3/Xa26, Xa4, Xa22, Xa32(t), Xa35(t), Xa36(t), Xa40* |
| *Xoo* | BAI3 | 11 |  | 114.6 | S11_28656770 | S11_28697227 | S11_27831677 | S11_28733787 | 3.6 | 902 | 56.1 | 9.1E-10 | 13.2 | All | *** | qABB-11 | *Xa3/Xa26, Xa4, Xa22, Xa32(t), Xa35(t), Xa36(t), Xa40* |
| *Xoo* | MAI70 | 11 |  | 114.6 | S11_28656770 | S11_28697227 | S11_27817978 | S11_28733787 | 3.7 | 916 | 28.4 | 1.9E-04 | 6.5 | All except SHZ-2 | * | qABB-11 | *Xa3/Xa26, Xa4, Xa22, Xa32(t), Xa35(t), Xa36(t), Xa40* |
| *Xoo* | MAI133 | 11 |  | 114.6 | S11_28656770 | S11_28697227 | S11_27831677 | S11_28977691 | 4.6 | 1146 | 25.1 | 7.3E-04 | 6.4 | All except SHZ-2 | * | qABB-11 | AQBT023, *Xa3/Xa26, Xa4, Xa22, Xa32(t), Xa35(t), Xa36(t), Xa40* |
| *Xoo* | MAI136 | 11 |  | 114.6 | S11_28656770 | S11_28697227 | S11_28010037 | S11_28697250 | 2.7 | 687 | 32.1 | 3.8E-05 | 8.4 | All | *** | qABB-11 | *Xa3/Xa26, Xa4, Xa22, Xa32(t), Xa35(t), Xa36(t), Xa40* |

**Continuation Table S7**

| **Pathovar** | **Strain** | **Chr** | **QTL** | **QTL position** | | | **Supporting interval** | | **Interval size** | | **Wald** | **p-value** | **Phenotypic** | **Predicted parental source** | **Corroboration** | **Known resistance loci to** | |
| --- | --- | --- | --- | --- | --- | --- | --- | --- | --- | --- | --- | --- | --- | --- | --- | --- | --- |
|  |  |  |  | **(cM)** | **Left Mrk** | **Right Mrk** | **Left Mrk** | **Right Mrk** | **(cM)** | **(kb)** |  |  | **variance (%)** | **of resistance** | **GWAS** | **African**  ***X. oryzae* strains** | **Asian**  ***X .oryzae* strains** |
| *Xoc* | MAI77 | 12 |  | 0.9 | S12_356349 | S12_358464 | S12_290036 | S12_413400 | 0.5 | 123 | 25.0 | 7.5E-04 | 6.1 | IR46, SHZ-2 |  |  |  |
| *Xoc* | MAI46 | 12 |  | 28.2 | S12_7181057 | S12_7185266 | S12_7181057 | S12_7186890 | 0.02 | 6 | 54.4 | 2.0E-09 | 14.2 | All except FED |  |  | qBB-12 |
|  |  |  |  |  |  |  |  |  |  |  |  |  |  |  |  |  |  |
| *Xoo* | MAI70 | 12 | qXO-12-1 | 70.7 | S12_17786177 | S12_17902596 | S12_17780296 | S12_17902596 | 0.5 | 122 | 24.8 | 8.1E-04 | 5.5 | IR77, SHZ-2 |  |  | AQBT029 |
| *Xoc* | MAI67 | 12 |  | 71.7 | S12_18039016 | S12_18060761 | S12_18021980 | S12_18084463 | 0.2 | 62 | 26.2 | 4.6E-04 | 6.4 | IR77 |  |  | AQBT029 |
| *Xoc* | MAI10 | 12 |  | 71.8 | S12_18060777 | S12_18084420 | S12_18021980 | S12_18274373 | 1.0 | 252 | 30.8 | 6.8E-05 | 7.6 | nd |  |  | AQBT029 |
| *Xoc* | MAI46 | 12 |  | 71.8 | S12_18060777 | S12_18084420 | S12_18021980 | S12_18084463 | 0.2 | 62 | 30.7 | 7.2E-05 | 7.6 | IR46, PSBRc82, SHZ-2 |  |  | AQBT029 |
|  |  |  |  |  |  |  |  |  |  |  |  |  |  |  |  |  |  |
| *Xoc* | MAI61 | 12 |  | 100.1 | S12_24462484 | S12_25156802 | S12_24462484 | S12_25613282 | 4.6 | 1151 | 28.5 | 1.8E-04 | 7.4 | nd |  |  |  |

**Table S8**. Comparison of QTL estimates for MAGIC indica S4 and S8 subsets for *Xoc* BAI5 and *Xoo* BAI3. Stars in corroboration GWA panel indicate level of significance of GWAS markers as follows: (*) *P*-value < 0.001; (***) *P*-value < 0.0001 and q-value < 0.05.

| **MAGIC** | **Strain** | **Chr** | **QTL position** | | | **Supporting interval** | | **Interval size** | | **Wald** | **p-value** | **N° predicted** | **Corroboration** |
| --- | --- | --- | --- | --- | --- | --- | --- | --- | --- | --- | --- | --- | --- |
| **subset** |  |  | **(cM)** | **Left Mrk** | **Right Mrk** | **Left Mrk** | **Right Mrk** | **(cM)** | **(kb)** |  |  | **genes** | **GWAS** |
| S4 | *Xoc* BAI5 | 4 | 123.6 | S4_30738922 | S4_31079998 | S4_30383295 | S4_31882603 | 6.0 | 1,499 | 48.4 | 2.9E-08 | 223 | * |
| S8 | *Xoc* BAI5 | 4 | 126.8 | S4_31819592 | S4_31872983 | S4_31786637 | S4_31872983 | 0.3 | 86 | 216.9 | 3.0E-43 | 14 | *** |
|  |  |  |  |  |  |  |  |  |  |  |  |  |  |
| S4 | *Xoo* BAI3 | 4 | 126.1 | S4_31666827 | S4_31716597 | S4_30383295 | S4_31992079 | 6.4 | 1,609 | 56.1 | 8.9E-10 | 239 | * |
| S8 | *Xoo* BAI3 | 4 | 126.8 | S4_31803168 | S4_31812015 | S4_31506827 | S4_31900997 | 1.6 | 394 | 90 | 1.1E-16 | 58 | *** |
|  |  |  |  |  |  |  |  |  |  |  |  |  |  |
| S4 | *Xoo* BAI3 | 5 | 4.0 | S5_1065077 | S5_1073143 | S5_761076 | S5_1258099 | 2.0 | 497 | 47.4 | 4.8E-08 | 77 | * |
| S8 | *Xoo* BAI3 | 5 | 1.3 | S5_347328 | S5_353165 | S5_37878 | S5_453169 | 1.7 | 415 | 45 | 1.3E-07 | 74 | *** |
|  |  |  |  |  |  |  |  |  |  |  |  |  |  |
| S4 | *Xoc* BAI5 | 10 | 76.0 | S10_19063585 | S10_19107872 | S10_18851296 | S10_19354685 | 2.0 | 503 | 25.0 | 7.7E-04 | 86 |  |
| S8 | *Xoc* BAI5 | 10 | 46.23 | S10_11626385 | S10_11634014 | S10_11575914 | S10_11743048 | 0.7 | 167 | 28.4 | 1.9E-04 | 17 |  |
|  |  |  |  |  |  |  |  |  |  |  |  |  |  |
| S4 | *Xoo* BAI3 | 11 | 114.6 | S11_28483987 | S11_28652283 | S11_27573854 | S11_28988578 | 5.7 | 1,415 | 41.8 | 5.8E-07 | 214 | * |
| S8 | *Xoo* BAI3 | 11 | 114.6 | S11_28656770 | S11_28697227 | S11_27831677 | S11_28733787 | 3.6 | 902 | 56 | 9.1E-10 | 118 | *** |

**Table S9.** QTL effective to multiple *X. oryzae* strains found in this study. Individual IM results (Table S7) were combined using the widest interval shared by the different *X. oryzae* strains to show common regions associated with resistance. Wald statistic for test that no parents have effects on trait (Wald, 7 df). Stars next to strains name indicate level of significance of GWAS markers as follows: (*) *P*-value < 0.001, (***) *P*-value < 0.0001 and q-value < 0.05.

| **Chr** | **QTL** | **Pathovar** | **QTL position** | | | **Supporting interval** | | **Interval size** | | **IM strains** | **Additional strains** | **N° strains** | **Known resistance loci to** | |
| --- | --- | --- | --- | --- | --- | --- | --- | --- | --- | --- | --- | --- | --- | --- |
|  |  |  | **(cM)** | **Left Mrk** | **Right Mrk** | **Left Mrk** | **Right Mrk** | **(cM)** | **(kb)** |  | **(only GWAS)** |  | **African**  ***X. oryzae* strains** | **Asian**  ***X. oryzae* strains** |
| 1 | qXO-1-1 | both | 5.2-5.9 | S1_1335951 | S1_1500023 | S1_1264553 | S1_1550887 | 1.1 | 286 | *Xoc* MAI123, | none | 3 |  |  |
|  |  |  |  |  |  |  |  |  |  | *Xoo* MAI72, Xoo MAI130 |  |  |  |  |
|  |  |  |  |  |  |  |  |  |  |  |  |  |  |  |
| 2 | qXO-2-1 | both | 96.3-107.8 | S2_24122049 | S2_26993900 | S2_23819009 | S2_27466604 | 14.6 | 3,648 | *Xoc* BAI5*, BLS256***, MAI10*, MAI46*, MAI61*, MAI77, MAI139 | *Xoc* MAI67*, MAI123* | 12 |  |  |
|  |  |  |  |  |  |  |  |  |  | *Xoo* MAI130 | *Xoo* BAI3*, MAI134* |  |  | Qbr2a |
|  |  |  |  |  |  |  |  |  |  |  |  |  |  |  |
| 2 | qXO-2-2 | both | 141.1-142.9 | S2_35289602 | S2_35781025 | S2_35068072 | S2_35891364 | 3.3 | 823 | *Xoc* MAI139 | none | 3 |  |  |
|  |  |  |  |  |  |  |  |  |  | *Xoo* MAI101, MAI130* |  |  |  | AQBT001, qBbr2b, *Xa24(t)* |
|  |  |  |  |  |  |  |  |  |  |  |  |  |  |  |
| 4 | qXO-4-1 | both | 125.8-127.7 | S4_31553264 | S4_32064419 | S4_30862506 | S4_32134600 | 5.1 | 1,272 | *Xoc* BAI5***, MAI10***, MAI67***, MAI77*, MAI139* | *Xoc* MAI46*, MAI61*, MA123* | 16 | *Xo1* |  |
|  |  |  |  |  |  |  |  |  |  | *Xoo* BAI3***, CFBP1951*, MAI70***, MAI101***, MAI130***, MAI136***, MAI145*** | *Xoo* MAI133* |  | *Xo1* | AQBT008, *Xa1, Xa2, Xa31(t), Xa38* |
|  |  |  |  |  |  |  |  |  |  |  |  |  |  |  |

**Continuation Table S9.**

| **Chr** | **QTL** | **Pathovar** | **QTL position** | | | **Supporting interval** | | **Interval size** | | **IM strains** | **Additional strains** | **N° strains** | **Known Resistance to** | |
| --- | --- | --- | --- | --- | --- | --- | --- | --- | --- | --- | --- | --- | --- | --- |
|  |  |  | **(cM)** | **Left Mrk** | **Right Mrk** | **Left Mrk** | **Right Mrk** | **(cM)** | **(kb)** |  | **(only GWAS)** |  | **African**  ***X. oryzae* strains** | **Asian**  ***X. oryzae* strains** |
| 5 | qXO-5-1 | both | 0.1-1.3 | S5_69530 | S5_353165 | S5_37878 | S5_453169 | 1.7 | 415 | *Xoc* MAI61*, | *Xoo* MAI133* | 3 |  | qBLSr5a, |
|  |  |  |  |  |  |  |  |  |  | *Xoo* BAI3*** |  |  |  | qBbr5, xa5 |
|  |  |  |  |  |  |  |  |  |  |  |  |  |  |  |
| 5 | qXO-5-2 | both | 5.9-8.0 | S5_1494420 | S5_2046183 | S5_1027632 | S5_2167880 | 4.6 | 1,140 | *Xoc* MAI46*, MAI61 |  | 4 |  |  |
|  |  |  |  |  |  |  |  |  |  |  | *Xoo* BAI3*, MAI134*** |  |  |  |
| 5 | qXO-5-3 | both | 84.9-90.7 | S5_21255253 | S5_22750867 | S5_20924798 | S5_24383716 | 5.8 | 3,459 | *Xoc* BLS256, |  | 2 |  |  |
|  |  |  |  |  |  |  |  |  |  | *Xoo* MAI134*** |  |  |  | qBB-5-2, qBB5, qBB-5-2, AQW004, qBBR5 |
|  |  |  |  |  |  |  |  |  |  |  |  |  |  |  |
| 7 | qBB-7-1 | *Xoo* | 20.1-23.6 | S7_5097414 | S7_5993972 | S7_3622979 | S7_6502570 | 11.5 | 2,880 | *Xoo* CFBP1951***, MAI136* | none | 2 | qABB-7 (races A2, A3) | *xa8* |
|  |  |  |  |  |  |  |  |  |  |  |  |  |  |  |
| 7 | qXO-7-2 | both | 109.9-112.3 | S7_27578266 | S7_28179129 | S7_27479742 | S7_28469553 | 4.0 | 990 | *Xoc* MAI61, |  | 3 |  |  |
|  |  |  |  |  |  |  |  |  |  | *Xoo* MAI72* | *Xoo* MAI130* |  |  |  |
|  |  |  |  |  |  |  |  |  |  |  |  |  |  |  |
| 8 | qBLS-8-1 | *Xoc* | 102.6-102.7 | S8_25638183 | S8_25729831 | S8_25638183 | S8_26014015 | 1.5 | 376 | *Xoc* MAI77, MAI123 | none | 2 |  |  |
|  |  |  |  |  |  |  |  |  |  |  |  |  |  |  |

**Continuation Table S9.**

| **Chr** | **QTL** | **Pathovar** | **QTL position** | | | **Supporting interval** | | **Interval size** | | **IM strains** | **Additional strains** | **N° strains** | **Known Resistance to** | |
| --- | --- | --- | --- | --- | --- | --- | --- | --- | --- | --- | --- | --- | --- | --- |
|  |  |  | **(cM)** | **Left Mrk** | **Right Mrk** | **Left Mrk** | **Right Mrk** | **(cM)** | **(kb)** |  | **(only GWAS)** |  | **African**  ***X. oryzae* strains** | **Asian**  ***X. oryzae* strains** |
| 10 | qXO-10-1 | both | 79.6-80.8 | S10_19975243 | S10_20983368 | S10_19903199 | S10_20983368 | 4.3 | 1,080 | *Xoc* MAI10, MAI46 |  | 5 |  |  |
|  |  |  |  |  |  |  |  |  |  | *Xoo* CFBP1951 | *Xoo* MAI72*, MAI93* |  | qABB-10 (race A1) |  |
|  |  |  |  |  |  |  |  |  |  |  |  |  |  |  |
| 11 | qBB-11-1 | *Xoo* | 28.0-28.9 | S11_7012013 | S11_7244498 | S11_6647060 | S11_7616946 | 3.9 | 970 | *Xoo* BAI3*, MAI70*** | *Xoo* MAI101*, MAI130*, MAI136* | 5 |  |  |
|  |  |  |  |  |  |  |  |  |  |  |  |  |  |  |
| 11 | qXO-11-2 | both | 107.5-114.6 | S11_26879946 | S11_28697227 | S11_26393474 | S11_28977691 | 10.3 | 2,584 | *Xoc* BLS256*** | none |  |  |  |
|  |  |  |  |  |  |  |  |  |  | *Xoo* BAI3***, CFBP1951, MAI70*, MAI72, MAI101***, MAI133*, MAI136***, MAI145*** |  | 9 | qABB-11 (races A1, A2, A3) | QBbr11, AQBT023, *Xa3/Xa26, Xa4, Xa22, Xa32(t), Xa35(t), Xa36(t), Xa40* |
|  |  |  |  |  |  |  |  |  |  |  |  |  |  |  |
| 12 | qXO-12-1 | both | 70.7-71.8 | S12_17786177 | S12_18084420 | S12_17780296 | S12_18274373 | 2.0 | 494 | *Xoc* MAI10, MAI46, MAI67 | none | 4 |  | AQBT029 |
|  |  |  |  |  |  |  |  |  |  | *Xoo* MAI70 |  |  |  |  |

**Table S10**. Significantly associated SNP for resistance to multiple *X. oryzae* strains on chromosome 2, detected by GWAS in the MAGIC indica S8 subset. The locus ID for each SNP was predicted from the MSU7 rice reference annotation. Intergenic regions were defined as regions without predicted genes or located more than 1 Kb upstream of genes. Founders are indicated with letters as follows: A: IR4630-22-2-5-1-3; B: Fedearroz 50; C: IR77298-14-1-2-10; D: Shan-Huang Zhan-2; E: PSBRc82; F: Sambha Mahsuri + Sub1; G: PSBRc158; H: IR45427-2B-2-2B-1-1. A colored box on the donor column indicates that founder carries the R allele. A colored box on the *X. oryzae* strains columns indicates that the SNP was found to be significantly associated with resistance to that particular strain (*P*-value < 0.001).

**Table S11**. Significantly associated SNP for resistance to multiple *X. oryzae* strains on chromosome 4, detected by GWAS in the MAGIC indica S8 subset. The locus ID for each SNP was predicted from the MSU7 rice reference annotation. Intergenic regions were defined as regions without predicted genes or located more than 1 Kb upstream of genes. Founders are indicated with letters as follows: A: IR4630-22-2-5-1-3; B: Fedearroz 50; C: IR77298-14-1-2-10; D: Shan-Huang Zhan-2; F: Sambha Mahsuri + Sub1; G: PSBRc158; H: IR45427-2B-2-2B-1-1. A colored box on the donor column indicates that founder carries the R allele. A colored box on the *X. oryzae* strains columns indicates that the SNP was found to be significantly associated with resistance to that particular strain (*P*-value < 0.001).

**Table S12**. Significantly associated SNP for resistance to multiple *X. oryzae* strains on chromosome 11, detected by GWAS in the MAGIC indica S8 subset. The locus ID for each SNP was predicted from the MSU7 rice reference annotation. Intergenic regions were defined as regions without predicted genes or located more than 1 Kb upstream of genes. Founders are indicated with letters as follows: A: IR4630-22-2-5-1-3; B: Fedearroz 50; C: IR77298-14-1-2-10; D: Shan-Huang Zhan-2; E: PSBRc82; G: PSBRc158; H: IR45427-2B-2-2B-1-1. A colored box on the donor column indicates that founder carries the R allele. A colored box on the *X. oryzae* strains columns indicates that the SNP was found to be significantly associated with resistance to that particular strain (*P*-value < 0.001).
